# Supplementary material for: Digital Registrar: A Schema-First Framework for Multi-Cancer Privacy-Preserving Pathology Abstraction via Local LLMs
Source: Diagnostics (Basel). 2026 May 27;16(11):1644. doi: 10.3390/diagnostics16111644 (PMC13257040; doi:10.3390/diagnostics16111644)
Supplement: Supplementary file 1 [file diagnostics-16-01644-s001.zip › diagnostics-4271493-supplementary.pdf]

Supplementary Materials

Digital Registrar: A Schema-First Framework for Multi-Cancer Privacy-Preserving Pathology Abstraction via Local LLMs

This document compiles every supplementary artefact referenced by the revised manuscript: the Section S1 Extended Methods sub-archive (Sections S1.1–S1.4), Supplementary Tables S1–S64, Supplementary Figures S1–S16 (rendered inline), and Supplementary Items S1–S10. Cross-references in the main text and in the response-to-reviewers letters resolve here. All numerical content derives from the canonical CSV outputs at paper-revision/code/derived/*.csv, regenerated by paper-revision/code/analysis.py from the evaluation results released on 2026-05-14 (git SHA 3818a004).

Index

Extended Methods (Section S1) — Section S1.1 Annotation Protocol; Section S1.2 Baseline Implementations; Section S1.3 Statistical Framework; Section S1.4 Decoding and Hardware

Supplementary Tables S1–S10 — Per-organ schemas

Supplementary Tables S11–S13 — Organ-classification confusion and stability (30-run aggregate)

Supplementary Tables S14–S23 — Per-field accuracy by organ (30-run aggregate)

Supplementary Table S24 — Lymph-node concordance (v1 carryover)

Supplementary Tables S25–S26 — Section 3.2 Triage and organ-classification 30-run aggregates

Supplementary Table S27 — Section 3.3 Per-organ extraction headline (Table 5 / Figure 3 backing)

Supplementary Table S28 — Section 3.3.1 Anatomic vs pathologic stage_group (Figure 4 backing)

Supplementary Tables S29, S64 — Section 3.3.2 Bottom-decile field error attribution

Supplementary Tables S30–S31 — Multi-primary triage

Supplementary Tables S32–S34 — Section 3.4 Margins and lymph nodes

Supplementary Table S35 — Section 3.8 Multi-run reliability

Supplementary Tables S36–S38 — Section 3.9 TCGA external validation

Supplementary Tables S39–S43 — Section 3.10 Baseline cascade comparison

Supplementary Tables S44–S54 — Section 3.6 Component ablations

Supplementary Tables S55–S63 — Section 3.7 Inter-annotator agreement and pre-annotation effect

Supplementary Figures S1–S16 — Rendered visual companions (PNG embeds)

Supplementary Items S1–S10 — Items S1–S7 case studies; Items S8–S10 Section 4 Discussion deep dives

Section S1. Extended Methods

Supplementary Section S1.1 — Annotation Protocol (Extended Methods)

This file expands main-body Section 2.1 (Data Source and Annotation) with the detailed annotation-committee protocol, the without-LLM-seed bias-control arm design, the senior-pathologist adjudication audit-trail mechanics, and the annotation tooling reference. The main-body Section 2.1 carries the cohort table, the high-level committee structure (annotators + adjudicator), and a one-sentence pointer to this file for the full detail.

Section S1.1.1 Reference Standard Construction (full detail)

The gold standard used throughout Section 3 was produced by a two-pathologist annotation committee — **Kai-Po Chang (KPC)** and **Nan-Haw Chow (NHC)**, both co-authors — with adjudication by a senior third pathologist, **Han Chang**, also a co-author. The senior-pathologist adjudication role has been part of our annotation workflow throughout the project; for this revision the annotation pipeline was re-run from scratch under a dedicated GUI annotation tool that retains the per-annotator paired record (enabling inter-annotator agreement statistics) and records the senior pathologist's adjudication decisions as a persistent audit trail. The committee design, the without-LLM-seed bias-control arm described below, and the audit-trail persistence together address Reviewer 1's same-model-bias concern (R1.b) and Reviewer 2's two-reviewer-panel concern (R2.3-IAA).

Section S1.1.2 Without-LLM-seed bias-control arm

To directly test the same-model-bias concern raised in R1.b — that annotators seeded with the candidate model's output may unconsciously anchor on its predictions, inflating apparent system performance — both pathologists completed an independent **without-LLM-seed re-annotation pass** on a stratified 196-case subset (~22 % of the validation cohort, balanced across organs). In this arm, the source pathology report was presented without the LLM pre-annotated JSON draft; each pathologist produced a labelled record from scratch. The per-pathologist labels in this arm are persisted independently by the annotation tool. The per-organ allocation of the 196-case subset is shown in table below; the stratification preserves the cohort-balance properties used in Section 3.3.

Per-organ allocation of the 196-case without-LLM-seed bias-control subset.

Organ site	Number of paired re-annotations (n)
Esophagus	19
Stomach	21
Colorectum	18
Breast	20
Uterine cervix	20
Pancreas	17
Prostate	17
Lung	19
Liver	20
Thyroid	20
Others (dual or out-of-scope cancers)	5

**Total**	**196**

The Δκ analysis comparing with-seed and without-seed inter-annotator κ (mean Δκ = +0.015, median 0.0 across the 93 (annotator × organ × field) cells with defined Δκ) is reported in Section 3.7. Δκ is computed under a strict **paired-annotator pairing constraint** — for each cell, the same partner annotator is used in both arms (KPC-with-seed × NHC-with-seed vs KPC-without-seed × NHC-without-seed, on the 196-case intersection) — so the κ baseline is held fixed across arms and only the seed-arm marginal varies. Implementation: evaluation_code/docs/eval/preann_effect.md. This confirms that the pre-annotation seed does not systematically anchor the annotators on the LLM's output.

Section S1.1.3 With-LLM-seed primary arm (operational workflow)

Both pathologists also re-annotated all 893 reports independently in the with-LLM-seed arm — the operational workflow the manuscript proposes for downstream clinical deployment, in which the gpt-oss-20b single-pass JSON output is presented as the editable draft and the pathologist edits in place. Each pathologist produced their own labelled record under the new annotation tool. The committee-resolved version of these new paired re-annotations — produced by senior-pathologist adjudication of residual disagreements — forms the `gold` reference used throughout Section 3. The original-submission consensus labels are not used in any Section 3 result.

Section S1.1.4 Why the new annotation pass was necessary

The original-submission protocol described two pathologists independently reviewing and correcting the LLM pre-annotation in a side-by-side view, with discrepancies resolved through blinded consensus. That side-by-side view was an ad-hoc two-pane editor setup, not a dedicated annotation tool, and the workflow was designed to converge on a single committee-resolved gold label per case; a v1 disagreement log was retained for use during adjudication, but a discrepancy log is the wrong artifact for κ at this schema's scale. Cohen's κ is defined on the full paired record — the chance-agreement baseline depends on the marginal distribution of *all* labels each annotator emitted, not just the cells where they disagreed — and at 41 categorical scalar fields per report on the with-seed κ scope, plus three nested variable-length list fields (`margins[*]`, `regional_lymph_node[*]`, and `biomarkers[*]`) carrying their own internal sub-object structure entirely outside that count, even raw per-annotator JSON dumps would still need a substantial schema-aware canonicalization pipeline before any cell-by-cell κ could be computed. The new annotation tool was engineered to persist each annotator's labels directly in canonical form, eliminating that retrospective step and producing the paired record on which Section 3.7's κ is computed.

Section S1.1.5 Adjudication: same human process, newly persisted audit trail

Han Chang's adjudication role is not new to this revision — disagreements between KPC and NHC have always been resolved in adjudication meetings with him as the senior pathologist. What is new is that for this revision, every consensus-resolving interaction during adjudication is recorded by the annotation tool as a persistent, structured artifact: (case, field, KPC value, NHC value, adjudicator's resolution). For each (organ, field) cell with at least one disagreement, the adjudicator's resolutions are tabulated as a 2 × 2 contingency table on the (matches_KPC, matches_NHC) outcomes (where a "matches" outcome means the adjudicator's value equals that annotator's submitted value, and a "matches both" cell is omitted because there is no disagreement to resolve). A per-cell χ^2 **uniformity test against the equal-probability null** (50 % KPC-favoured, 50 % NHC-favoured) is then

computed (df = 1; exact-binomial fallback when n_disagreements < 10); p-values are Holm-corrected across the 230 (organ, field) cells. The null is retained on the great majority of cells — the adjudicator's resolutions are uniform across (matches_KPC, matches_NHC), with no systematic bias toward either annotator. Full table: Supplementary Table S58. The original-submission manuscript wording "final blinded consensus" did not credit Han Chang explicitly; this is corrected here by naming all three pathologists and by persisting the adjudication decisions as auditable data.

Section S1.1.6 Annotation Tooling

The dedicated GUI annotation tool built for this revision is open source at <https://github.com/kblab2024/digital-registrar-research/tree/main/src/digital_registrar_research/annotation>. The tool is structured as two complementary applications. `app_canonical.py` is the per-annotator labelling app: each pathologist runs their own instance; the app presents the source pathology report and an editable JSON view in a single window, supports both with- and without-LLM-seed arms (the JSON view can be initialized empty or pre-populated with the gpt-oss-20b single-pass output), and persists each annotator's labels as an independent record in canonical form keyed by annotator identity. `compare_app.py` is the adjudication and consensus-building app, used by the senior pathologist to resolve disagreements between KPC and NHC; every consensus-resolving interaction is recorded by the app as a persistent log entry, producing the structured adjudication audit trail that backs Supplementary Table S58. Implementation detail (UI layout, hotkeys, persistence schema) is available in the open-source source code.

Cross-references

Cohen's κ is defined and discussed as the gold-standard validity metric in Section 2.4 (Evaluation Protocol and Statistical Methods) and Supplementary Section S1.3. The headline inter-annotator agreement on the committee, the Δκ analysis on the without-LLM-seed arm, and the per-(organ, field) committee-adjudication audit are reported in Section 3.7 . Δκ tables are in Supplementary Tables S55–S63.

Supplementary Section S1.2 — Baseline System Implementations (Extended Methods)

This file expands main-body Section 2.6 (Baseline Systems for Cross-Architecture Comparison) with the detailed implementation specifications for each of the four reference systems benchmarked on the TCGA external cohort. Main-body Section 2.6 retains the high-level rationale (four-system comparison: rule-based, encoder, local LLM, API LLM) and the result-anchor pointer to Section 3.10; this file holds the implementation specifics that reviewers may need for reproduction.

Section S1.2.1 Why TCGA, why these four systems

To contextualise the proposed local-LLM pipeline against established methods, four systems were benchmarked head-to-head on the TCGA external cohort (Section 2.5). TCGA is the only corpus that admits a fair four-way comparison — the closed-frontier API model cannot be sent CMUH data for privacy reasons, and the BERT baseline was fine-tuned on CMUH so predicting on CMUH would leak training cases. All four systems consumed the same plain-text reports and emitted the same per-organ schema; all were scored under the identical Stage A/B/C cascade defined in Section 2.4.

Section S1.2.2 Rule-based system (deterministic floor)

A deterministic regex- and lexicon-driven extractor implemented in `benchmark_code/baselines/rules.py`, spanning ten organ schemas. Per-organ tactics include lexicon majority-vote for organ classification, longest-match enums for TNM, organ-aware grade extractors (Gleason → ISUP for prostate; Nottingham sub-scores for breast), and numeric span extractors with unit normalization. Coverage policy is `omit-on-no-signal`: a field is emitted only when a regex / lexicon match clears its confidence threshold. Full inventory: evaluation_code/docs/benchmarks/06_methods.md.

Section S1.2.3 BERT-merged system (encoder-only reference)

`emilyalsentzer/Bio_ClinicalBERT` as a shared encoder behind a multi-head linear classifier (`clinicalbert_cls.py`; one head per categorical or boolean field, with an explicit `null` class) and a SQuAD-style extractive QA head for numeric span fields (`clinicalbert_qa.py`). Fine-tuned on the full CMUH annotation pool (5 epochs CLS head, 3 epochs QA head) and predicted on the held-out TCGA cohort — the only fair training configuration given privacy and disjointness constraints. Nested variable-length list fields cannot be emitted from a CLS + QA architecture (`n_attempted = 0` by construction); this is a structural ceiling. Hyperparameter detail: evaluation_code/docs/benchmarks/02_train_bert.md.

Section S1.2.4 Local LLM (gpt-oss-20b via DSPy + Ollama)

The proposed system. `OpenAI gpt-oss-20b` is served by Ollama with `llama.cpp` grammar-constrained decoding; DSPy `Signature` declarations encode each per-organ schema as `Literal[...]` enums and typed nested lists, which the DSPy adapter compiles to a JSON Schema that Ollama (≥ 0.5) converts to a GBNF grammar masking tokens that would violate the schema at sample time. Inference runs on-prem; no PHI leaves the institutional perimeter. Serving stack and grammar pipeline: evaluation_code/docs/dspy_deep_dive.md Section 1, evaluation_code/docs/pipeline.md.

Section S1.2.5 API LLM (gpt-5.4-mini via OpenAI)

`gpt-5.4-mini` accessed through the OpenAI API as a closed-frontier reference, reusing the same DSPy Signatures but routing constrained decoding through OpenAI's native `response_format={"type": "json_schema"}` mode rather than llama.cpp GBNF. Retry-on-parse-error is enabled; decoding parameters are pinned by the project's `MODEL_PROFILES` config. For this benchmark, the commercial API was called with `num_ctx = 4,096` while the local model was served with `num_ctx = 16,384` — a **4× asymmetry favouring the local model** that we did not have time to rebalance in this revision. The discussion of what this asymmetry does and does not establish is in Section S1.2.7 below. API-call shape: evaluation_code/docs/benchmarks/03_run_baselines.md.

Section S1.2.6 Cascade evaluation and error propagation

All four systems were scored under the three-stage cascade defined in Section 2.4 — Stage A (eligibility triage), Stage B (organ classification), Stage C (per-organ field extraction). A Stage-A or Stage-B failure halts the cascade for that attempt; no downstream stages are scored and the attempt counts as the failed stage's error in the cascade-level denominator. Stage-C per-attempt accuracy is computed only on attempts that survive both gating stages, and the per-attempt denominators read identically across systems on shared reports. Nested-list fields are scored separately under list-F1 (Section 2.5) and are not pooled into the Stage-C scalar denominator.

Section S1.2.7 Cross-architecture comparison: what the result does and does not establish

The four-system experiment was designed to answer **R1.a — methodological baseline contextualisation**. The two methodologically load-bearing baselines are the deterministic rule-based extractor (engineering floor: cannot reach fields outside its regex inventory) and the BERT encoder (representational ceiling: cannot emit variable-length nested structures by construction); the local language model clears both at per-field extraction, which is the comparison the reviewer's request anchors on.

A fourth contextualising arm — the commercial `gpt-5.4-mini` API — was added because the reviewer named "GPT-4" alongside the methodological baselines. Closed-frontier commercial models are not, strictly, methodological baselines in the encoder-vs-rule-vs-language-model sense; they are a comparison against a different deployment regime (off-prem, paid, rate-limited) rather than a different model class. We did not configure that comparison for a balanced head-to-head:

- The local model was served with `num_ctx = 16,384` while the commercial API was called with `num_ctx = 4,096` (a 4× asymmetry favouring the local model — pathology reports plus the per-organ output schema together routinely exceed 4 K tokens). This was an authors' configuration decision and not rebalanced before the in-revision deadline.
- The commercial system used was `gpt-5.4-mini`, which is the smaller, faster sibling of a frontier commercial family, not the full `gpt-5.4` variant.

What the cross-architecture experiment does establish:

1. All four systems collapse on the same set of TCGA caveat-tagged fields (Supplementary Table S38), confirming that the residual external-validation gap reported in Section 3.9 is a property of the corpus rather than of the extraction model. This is the load-bearing finding for R1.e.
2. Rule-based and BERT have structural ceilings that no amount of additional training data can close without changing the architecture — the rule-based system because its regex inventory cannot be exhaustive over the

schema, the BERT encoder because its classification + extractive-QA architecture has no representational primitive for nested variable-length structures (Supplementary Table S43).

3. The local language model clears the two methodological baselines by a wide margin at per-field extraction.

What the cross-architecture experiment does not establish:

- A categorical accuracy ordering between the local language model and frontier commercial language models. The local-vs-commercial result was: local ahead on per-field scalar extraction (Supplementary Table S39); commercial ahead on the two nested list fields (Supplementary Table S43). This scalar-vs-list divergence cannot be cleanly attributed to context-window capacity (the local model had 4× more context), and the smaller-sibling configuration on the commercial side leaves open whether the full commercial variant would lead on both surfaces.

Three discriminating experiments deferred to future work (each contingent on resources not available within this revision):

1. A matched-context API rerun at `num_ctx = 16,384` on the same 242 reports.

2. A comparison against the full `gpt-5.4` (not the mini variant).

3. An open-weights comparison against `gpt-oss-120b` — the larger sibling of our local 20 B model — under the same protocol and a comparable GPU footprint.

The paper's primary contribution is operational viability for on-prem registry extraction under a privacy constraint (no patient text leaving the institution), which does not depend on the local-vs-commercial accuracy ordering. The cross-architecture comparison is reported because it answers R1.a and supports R1.e; it is not framed as a categorical accuracy claim.

Setion S1.2.8 Corpus-side OCR caveat (TCGA-Reports)

The TCGA-Reports release we use is OCR-derived from the original PDF reports via Amazon Textract with custom post-processing. The corpus authors (Kefeli & Tatonetti, 2024) explicitly describe the result as "**moderately curated**" and acknowledge residual spelling errors (introduced either in the original text or during OCR), systematically mis-translated handwriting (dropped at corpus-release time), and exclusion of multiple-choice and synoptic report formats from the released plain text. This corpus-side noise contributes an unquantified component of the per-field extraction residual error in Section 3.9 / Section 3.10. It affects **all four benchmarked systems symmetrically** — rule-based, BERT, local language model, and commercial API all operate on the same Kefeli & Tatonetti plain-text release — so it does not bias the cross-architecture ranking. Quantifying this source-text-quality contribution would require a character-level audit of the OCR-cleaned text against the source PDFs and is outside the scope of this revision; the main-body external-validation paragraph (Section 2.5) carries a one-sentence pointer to this subsection.

Supplementary Section S1.3 — Statistical Framework (Extended Methods)

This file expands main-body Section 2.4 (Evaluation Protocol and Statistical Methods) with the detailed statistical formulas, multiple-comparison protocols, GLMM specification, and library version pinning. Main-body Section 2.4 carries the multi-run protocol summary (k = 30 internal, k = 22 external), the Wilson / McNemar / ICC / Krippendorff α framework names, Table 2 (metrics definitions), and a one-sentence pointer to this file. The deep derivations and library calls live here.

Section S1.3.1 Component Ablation Protocol

Operational definitions and cell composition

Two operational definitions feed the ablation grid. **DSPy `Literal` typing** is the use of `typing.Literal[...]` enum annotations inside `dspy.Signature` field declarations; the DSPy adapter consumes these annotations to emit a JSON Schema enum constraint that the downstream constrained-decoder enforces. **The `ReportJsonize` pre-pass** is the upstream DSPy signature that converts raw pathology-report paragraphs into a semi-structured JSON-like summary (`report_jsonized`) before the downstream extraction signature runs. The cells `dspy_monolithic` and `dspy_monolithic_no_jsonize` differ only on whether this pre-pass runs; full definitions are in evaluation_code/docs/dspy_deep_dive.md Section 1 and evaluation_code/docs/pipeline.md.

To quantify the contribution of the pipeline's engineering choices (per-organ decomposition, the `ReportJsonize` pre-pass, DSPy `Literal` typing, schema-constrained generation), a five-cell lesion study was run on the same internal CMUH cohort against the same committee-resolved gold (Section 2.4.2). The five cells span the proposed pipeline (`dspy_modular`, 10 multi-seed runs) and four single-axis lesions (`dspy_monolithic`, `dspy_monolithic_no_jsonize`, `raw_json`, `free_text_regex`). Compute budget restricted the four non-modular cells to one seed each; the within-cell stochasticity is bounded by the Fleiss κ across the modular cell's ten seeds (Supplementary Table S51). Headline results are reported in Section 3.6. The design rationale — non-orthogonality of the named axes, which matched-pair effects the grid does and does not cleanly isolate, the seed-count asymmetry, and the lesion-study framing — is documented below.

Cell-by-cell mechanical definitions

This subsection is the authoritative mechanical reference for each ablation cell. The full variant–component matrix, the per-variant descriptions in Section 3.6 and rebuttal R1c, and the per-stage rollup in Supplementary Table S54 all resolve here. Runner-level implementation paths are listed below; the canonical CMUH ablation manifest is `manifest.json` for the complete ablation result tree (git SHA `ba35efb`).

Variant–component matrix. ✓ = on, ✗ = off, n/a = not applicable.

Variant	Per-organ decomp	DSPy framework	`ReportJsonize` pre-pass	DSPy `Literal` typing	OpenAI `json_object` mode	Post-hoc `jsonschema` + retry	Regex + fuzzy extractor
------------------------------	:-:	:-:	:-:	:-:	:-:	:-:	:-:
`dspy_modular` (proposed)	✓	✓	✓	✓	n/a	n/a	✗
`dspy_monolithic`	✗	✓	✓	✓	n/a	n/a	✗
`dspy_monolithic_no_jsonize`	✗	✓	✗	✓	n/a	n/a	✗
`raw_json`	✗	✗	✗	✗	✓	✓	✗

free_text_regex	✗	✗	✗	✗	✗	✗	✓

- **`dspy_modular`** (runners/dspy_modular.py) — the proposed pipeline. Per-organ specialisation is realised as 5–7 chained dspy.Signature calls per organ; the ReportJsonize signature (models/common.py, class ReportJsonize) is invoked first to convert raw paragraphs into a semi-structured JSON summary that is passed as report_jsonized input to the downstream extraction signatures; output fields use Literal[...] enum annotations that the DSPy adapter compiles into a constrained-decoder enum constraint. Outputs are returned as Pydantic-typed instances and serialised by dump_prediction_plain(). No explicit retry budget — DSPy handles backtracking on schema violations internally per its framework defaults.
- **`dspy_monolithic`** (runners/dspy_monolithic.py, MonolithicPipeline with skip_jsonize=False) — collapses the per-organ specialisation. The 50–80 organ-specific output fields are merged into a single dspy.Signature per organ by signatures/monolithic.py::get_monolithic_signature(), which iterates over the per-subsection signature classes (e.g., BreastCancerNonnested, BreastCancerStaging, …) and merges their output fields into one class. ReportJsonize and Literal-typed extraction are kept.
- **`dspy_monolithic_no_jsonize`** (runners/dspy_monolithic_no_jsonize.py, MonolithicPipeline with skip_jsonize=True) — additionally skips the ReportJsonize pre-pass. When skip_jsonize=True the forward() method passes an empty {} as the report_jsonized input rather than invoking the ReportJsonize signature on the paragraphs. The monolithic signature therefore reads the raw paragraphs directly.
- **`raw_json`** (runners/raw_json.py) — removes the DSPy framework entirely. The OpenAI client is instantiated directly (from openai import OpenAI), invoked with response_format={"type":"json_object"} (falls back to prompt injection if the backend does not support JSON mode), and the schema appears in the prompt as a plain-text bulleted field-list (flatten_schema_for_prompt() in schemas/schema_builder.py) rather than as Pydantic introspection of dspy.OutputField instances. Outputs are validated post-hoc by jsonschema.Draft202012Validator via validate_cancer_data(); if validation errors are present and fewer than 20, the call is retried once with the error list appended to the user message. The _schema_errors field is set on the output when validation fails after retry; the validation_retries counter is tracked in the ledger.
- **`free_text_regex`** (runners/free_text_regex.py, extractor extractors/regex_per_field.py::RegexExtractor) — removes schema discipline as well. The model receives a prose-only system prompt asking it to summarise the pathology report in plain English ("the cancer site, the tumour size, histologic grade, TNM stage (pT/pN/pM), the presence or absence of lymphovascular and perineural invasion, surgical margins, lymph-node involvement, and any biomarker results … Do NOT use JSON or bullet points — write it as natural prose"). The model output is a single prose paragraph with no schema awareness. The RegexExtractor applies a fixed bank of 20+ regex patterns over the prose for primary endpoints (tumor size, pT/pN/pM, grade, LVI/PNI, biomarker presence) and a fuzzy token-Jaccard ≥ 0.6 match against per-organ enum vocabularies for secondary categorical fields. Organ is inferred via keyword-frequency over an organ-specific lexicon. There is no retry, no JSON validation, and no list-emitting code path; variable-length structured-list fields (e.g., involved_margin_list) have n_attempted = 0 because the extractor produces no list outputs.

Chapter-3 field-type categories and the chapter-4 / 5 list-level evaluation

The component-ablation analysis in Section 3.6 and Supplementary Table S54 stratifies the cascade evaluation along the field structure dimension. This is *not* labelled with letter codes (A/B/C/…) to avoid collision with the cascade-pipeline "Stage A / Stage B / Stage C" terminology established in Section 2.4 and Section 2.5 (eligibility classification / organ classification / per-organ field extraction). Instead, the four categories are referred to in the upper-layer prose by plain-language names; their definitions are pinned here.

Chapter-3 scalar field types. Every chapter-3 in-scope field (62 fields, code/derived/field_complexity_stratification.csv) is assigned to exactly one of three types, fixed in `paper-revision/code/analysis.py::FIELD_COMPLEXITY_STAGE`:

- **boolean / single-flag** — the value is a single yes/no answer to a presence question. 20 fields, including `lymphovascular_invasion`, `perineural_invasion`, `vascular_invasion`, `dcis_present`, `intraductal_carcinoma_presence`, `extranodal_extension`, `extrathyroid_extension`, `extraprostatic_extension`, `seminal_vesicle_invasion`, `visceral_pleural_invasion`, `bladder_invasion`, `tumor_invasion`, `tumor_necrosis`, `signet_ring`, `tumor_budding`, `spread_through_air_spaces_stas`, `direct_invasion_of_adjacent_structures`, `cribriform_pattern_presence`, `extracellular_mucin`, and `margin_positivity` (a derived bool over the margin object list).
- **rigid enum (single-value categorical)** — the value is a single token drawn from a fixed vocabulary defined either by the AJCC 8th-edition staging system, by the WHO histology classifications, or by the per-organ enum vocabularies in the schema. 32 fields. This category is *not* split further — TNM categories (`pt_category`, `pn_category`, `pm_category`, `tnm_descriptor`, `anatomic_stage_group`, `pathologic_stage_group`, `stage_group`, `overall_stage`, `distant_metastasis`), histology / grade / laterality (`histology`, `grade`, `nuclear_grade`, `cancer_laterality`, `sideness`, `tubule_formation`, `total_score`, `mitotic_rate`, `mitotic_activity`, `dcis_grade`, `dcis_comedo_necrosis`, `dcis_size`, `depth_of_invasion_three_tier`), procedure / technique (`procedure`, `surgical_technique`, `type_of_polyp`, `tumor_focality`, `tumor_extent`, `tumor_site`, `tumor_extension`, `cancer_primary_site`, `cancer_quadrant`, `cancer_clock`) are all rigid enums in this sense — each value space is a finite, machine-defined vocabulary that the schema enforces.
- **numerical** — the value is a number (possibly with an implicit unit). 9 fields, including `tumor_size`, `prostate_size`, `prostate_weight`, `gleason_4_percentage`, `gleason_5_percentage`, `tumor_percentage`, `depth_of_invasion_number`, `margin_length` (numeric distance within the margin object list), and `maximal_ln_size` (numeric count over the LN-group list).

The chapter-3 `involved_margin_list` field is a chapter-3 summary view of the canonical chapter-4 evaluation and is *excluded* from the per-field-type aggregation in S54a. It remains in `ablation_per_field.csv` and `field_complexity_stratification.csv` as a per-field row for visibility.

Chapter 4 (surgical margins) and chapter 5 (lymph-node groups) list-level evaluation. These two cascade chapters score variable-length lists of structured objects and are the canonical evaluation of nested-list extraction. Both apply the per-element scoring rule defined in Section 2.4.1: predicted and gold lists are aligned greedily by canonical category (margin canonical label or LN canonical station), ties broken by Jaccard similarity on the free-text sub-fields (margin description or station name), and a matched element scores correct only when every sub-field exact-matches the aligned gold element. The chapter-level micro F1 reported in S54a and S54b/c is computed by pooling true positive (tp), false positive (fp), and false negative (fn) counts across the 10 organs

(with the per-(method × organ) breakdown, then computing micro F1 = 2 tp / (2 tp + fp + fn), hallucination rate = fp / (tp + fp), and miss rate = fn / (tp + fn) on the pooled counts.

The chapter-4 margin object has three attributes (canonical margin category, involved bool, optional distance in millimetres); the chapter-5 LN-group object has four (canonical anatomical station, side, examined count, involved count). This single-attribute difference between the two list-level schema elements is the load-bearing structural difference behind the asymmetric `raw_json` collapse pattern reported in Section 3.6 and Table S54: chapter 4 absorbs DSPy removal almost intact, chapter 5 collapses by 31.6 pp F1 on the pooled denominator and by 49–70 pp on four individual organs.

Non-orthogonality of schema, DSPy, and prompting

A naïve reading of "schema constraints, DSPy, prompting" treats these three as independently manipulable axes. In the pipeline studied here, they are not:

- The schema is realised through DSPy `Literal[...]` type hints in `dspy.Signature` declarations. There is no "schema without DSPy" cell — removing DSPy and retaining the schema would require a re-implementation of the constrained-output mechanism in a different framework (e.g., guidance, outlines, instructor), which would confound the engineering-choice question with a framework-substitution question.
- The prompting itself is partially produced by DSPy's compilation step, so there is no "DSPy without DSPy-style prompts" cell.
- The `ReportJsonize` pre-pass can be ablated within the monolithic DSPy regime — which is what the `dspy_monolithic_no_jsonize` cell does.

The five cells in the grid are therefore the cleanest set of single-component lesions the pipeline architecture admits:

Cell	Per-organ decomposition	DSPy + `Literal` typing	`ReportJsonize` pre-pass	Constrained generation
`**dspy_modular**` (proposed)	✓	✓	✓	✓
`dspy_monolithic`	✗	✓	✓	✓
`dspy_monolithic_no_jsonize`	✗	✓	✗	✓
`raw_json`	✗	✗	n/a	partial (JSON mode)
`free_text_regex`	✗	✗	n/a	✗

Reading this table column-by-column gives the lesion-study claim that the grid can support: each row removes one or more axes, and the question is which axes are cleanly isolable by a matched-pair test in the grid as run. Section Matched-pair effects the grid can and cannot isolate enumerates which matched pairs are unambiguous.

Matched-pair effects the grid can and cannot isolate

The five cells above admit four candidate single-axis matched-pair tests, but only two of them are unambiguous; the other two run in the opposite direction from a naïve reading of the Δ-vs-modular column. Specifically:

Axis being lesioned	Matched pair (cells differ only on this axis)	Direction	Paired Δ (case-level)	Raw Δ (per-organ macro)

Per-organ decomposition	`dspy_modular` vs `dspy_monolithic` (matched on DSPy + `ReportJsonize` + schema)	clean, **in favour of decomposition**	+0.71 pp (Holm p = 0.003)	+4.78 pp micro / ≈ +9.6 pp on breast
`ReportJsonize` pre-pass	`dspy_monolithic` vs `dspy_monolithic_no_jsonize` (matched on DSPy + schema, no per-organ decomp)	clean, **against the pre-pass in this monolithic regime** (removing it improves)	**−0.57 pp** (i.e., retaining the pre-pass hurts)	−0.57 pp micro / **−4.05 pp on breast**
DSPy `Literal` typing	`dspy_monolithic_no_jsonize` vs `raw_json` (matched on schema, no per-organ decomp, no `ReportJsonize`)	clean, **against DSPy typing in this regime** (`raw_json` 93.07 % > `dspy_monolithic_no_jsonize` 91.28 %)	**−1.79 pp** for having DSPy typing	confounded with output-mode change (`dspy.Predict` vs JSON-mode), not cleanly attributable to `Literal` typing alone
Schema vs schema-blind	`raw_json` vs `free_text_regex` (matched on absence of DSPy + decomp + `ReportJsonize`)	clean, **strongly in favour of schema**	−77.78 pp (Holm $p < 10^{-300}$)	catastrophic across all organs

The +2.31 pp gap that `raw_json` shows against the **modular** cell, and the −0.54 pp gap that `dspy_monolithic_no_jsonize` shows against the **modular** cell, are both **multi-axis comparisons confounded with per-organ decomposition** and should not be read as isolated component contributions. The grid as run therefore supports a clean lesion-study claim only for **per-organ decomposition** and for **schema vs schema-blind**. The two remaining axes are reported as honest within-axis matched-pair effects rather than "additive contributions" — and for `ReportJsonize`, the within-axis effect runs against the proposed configuration in the monolithic regime. This is surfaced transparently in Section 3.6 observation (3). A cleaner 3 × 3 × 3 factorial that would extend the within-monolithic `ReportJsonize` observation to the modular regime (specifically a `dspy_modular_no_jsonize` cell) is left for follow-up work; see Section Honest scope: what the grid cannot reach.

Seed-count asymmetry across cells

The 10-seed run of the modular cell against 1-seed runs of each alternative cell reflects a compute-budget choice, not a methodological one. The full 10-seed run of the modular cell takes approximately three days on the available hardware; multiplying by four for the alternative cells would have required roughly twelve additional days and was not budgeted for this revision cycle.

The within-cell stochasticity that the single-run estimates conceal is bounded by the modular cell's own seed-consistency statistics. The Fleiss κ across the ten modular seeds (per-field median 0.51, range 0.21–0.62; Supplementary Table S51) and the per-field accuracy SD across seeds (median ≈ 1.2 pp) bound the seed-to-seed variability that would be expected on any other DSPy-flavoured cell, because the underlying model and prompt template are identical. The smallest between-cell gap reported in Figure 6 / Supplementary Table S44 (0.54 pp for `dspy_monolithic_no_jsonize` vs modular, Holm-corrected p = 0.014) is larger than the per-field accuracy SD; the headline conclusions are not at risk from the 1-vs-10 seed asymmetry. A future ablation pass with matched

n_seeds across cells would tighten the CIs on the four non-modular cells and is planned for any subsequent revision.

Lesion-study framing

The grid is described as a **lesion study on engineering choices** rather than a **factorial decomposition of independent components**, for three reasons:

- The three components named in the standard reviewer prompt (schema constraints, DSPy, prompting) are mechanically entangled in this pipeline (Section Non-orthogonality of schema, DSPy, and prompting).
- The five cells are not a balanced factorial — they are an ordered sequence of single-axis lesions from the proposed configuration toward the schema-blind floor.
- Each cell is interpretable as "what happens when this engineering choice is removed" rather than "the marginal contribution of axis X holding axes Y, Z fixed".

This framing matches what the data can support. It is also the framing that the standard reviewer prompt accepts — "controlled experiments by removing or modifying each component to quantify its impact on performance" is exactly a lesion study. No clean orthogonal factorial is claimed in Section 3.6 or here.

Honest scope: what the grid cannot reach

Two follow-up grids are scoped but unrun in this revision cycle:

- **Grid 2 (3 × 3 × 3 factorial)** — pipeline decomposition × output-structuring discipline × prompting strategy. Would require approximately one week of additional compute; not budgeted.
- **Grid 3 (model-identity sweep)** — `gpt-oss:20b` vs `gemma3:27b` vs `qwen3:30b` vs `medgemma:27b` at matched compute. Would address the question of whether the present lesion-study effects are an artefact of the chosen base model; not budgeted.

The current ablation is sufficient to support the lesion-study claim made in Section 3.6; the broader factorial decomposition and the cross-model sweep are honest future work.

Section S1.3.2 Confidence Intervals

All proportions are reported with 95 % **Wilson score** intervals on the numerator and denominator stated for that table. For the upstream classification endpoints, repeated inferences are first collapsed by report-level majority vote, and the Wilson denominator is the number of independent reports. For scalar-extraction endpoints, repeated inferences are first collapsed by case-field majority vote, and the Wilson denominator is the number of eligible case-field units. Pooled case-run and case-run-field summaries remain descriptive repeated-inference summaries rather than independent-clinical-sample intervals inflated by the seed count. Small-cell counts (n_attempted < 30 or extreme proportions) are cross-checked against Clopper–Pearson exact intervals. Continuous metrics (per-case F1, Lin's CCC for paired counts, F1 deltas between methods) are reported with **bias-corrected accelerated (BCa) bootstrap** intervals (B = 2 000), resampled at the **case level** so that case-clustered run-to-run variance is preserved; the bias and acceleration estimates use `scipy.stats` distributional support.

Section S1.3.3 Hypothesis Tests and Multiple-Comparison Corrections

Pairwise tests on paired binary outcomes (e.g., field-level accuracy between two pipeline configurations on the same case set) used **McNemar's test** with continuity correction, falling back to the exact binomial when discordant pairs were below 25. Marginal-homogeneity tests on multi-class endpoints (e.g., the 11-class organ-

classification confusion table) used the **Stuart–Maxwell** statistic (Bhapkar variant when df > 1). Repeated-measures comparisons across three or more pipeline configurations on the same case set used the **Friedman test** with **Nemenyi** post-hoc, the standard non-parametric protocol recommended by Demšar (2006).

Within each comparison family (per-field × axis), p-values were corrected using **Holm–Bonferroni** for family-wise error control, with **Benjamini–Hochberg** FDR reported as a sensitivity check on the larger field × organ exploratory grid and **Benjamini–Yekutieli** FDR on dependent-test families. The Holm correction family scope is one family per endpoint tier .

Effect sizes were reported alongside p-values: **Cohen's κ** and **quadratic-weighted κ** for nominal/ordinal agreement (the weighting choice is per-field-type — unweighted for nominal-categorical fields and binary endpoints, quadratic-weighted for ordinal fields such as TNM and grade — and is assigned automatically by house-designed code per the field-type map, **Krippendorff's α** for mixed-type reliability, **Matthews' correlation** for class-imbalance-robust binary endpoints, and **Cliff's δ** for non-parametric continuous comparisons.

Section S1.3.4 Reliability Coefficients (Multi-Run)

Run-to-run reliability is a separate analysis from the Wilson intervals above. Reliability of the case × run accuracy matrix was summarised by:

- **ICC(2,1)** under a two-way random-effects model — single-run reproducibility;
- **ICC(3,k)** with k = 30 — reliability of the thirty-run mean (Spearman–Brown extrapolation from the single-rater statistic);
- **Cronbach's α** — internal consistency cross-check;
- **Accuracy flip rate** — proportion of cases that flipped between correct and incorrect across the thirty runs;
- **Per-case run SD** — within-case standard deviation of the per-case F1 score, summarised by mean and 90th percentile across cases.

Reliability bands followed the Shrout & Fleiss conventions (ICC ≥ 0.75 good, ≥ 0.90 excellent, ≥ 0.99 essentially perfect). Cross-seed agreement on the modular ablation cell is additionally summarised by **Fleiss κ across the 10 modular seeds**, treating each seed as a "rater" on per-case correctness and reporting κ per field with Landis–Koch interpretation bands; per-case F1 SD is the within-case standard deviation of the per-case F1 score across the seed family, summarised by mean and 90th-percentile across case. Detailed per-stage and per-organ results are presented in Section 3.8 and Supplementary Table S35.

Section S1.3.5 GLMM Specification for Ablation Variance Decomposition

For the per-attempt accuracy decompositions reported in Section 3.6 (Supplementary Tables S48 and S49), the variance attributable to case identity vs run identity vs design axes is summarised by a **generalised linear mixed model** with the following specification:

- **Response variable**: per-attempt correctness, binary 0/1, one row per (case, run, field) eligible attempt that survives Stage A/B gating.
- **Family / link**: binomial / logit.
- **Fixed effects**: a design matrix on the four ablation axes — per-organ decomposition (binary), DSPy `Literal` typing (binary), `ReportJsonize` pre-pass (binary), schema-constrained generation (binary) — entered as main effects; per-field fits use one row per (axis, field) cell.
- **Random effects**: random intercepts for `case_id` (within-case clustering across runs) and `run_id` (run-to-run shared offset on the modular cell's 10 seeds; degenerate for the four single-seed cells). The two-source

random-effect structure decomposes total per-attempt variance into case-clustered vs run-clustered components.

• **Implementation**: Bayesian variational fit via `statsmodels.genmod.bayes_mixed_glm.BinomialBayesMixedGLM` (default mean-field VI, 500 iterations, convergence threshold 1×10^{-5}). Effect estimates are posterior means with credible intervals from the posterior covariance.

• **Convergence fallback**: on non-convergence (rare; tracked per-field in Supplementary Table S49), the cell is re-estimated with a **two-source bootstrap** that resamples cases and runs independently and reports the empirical CI directly. The fallback choice is logged per row of Supplementary Tables S48 / S49 in a `convergence_status` column.

Section S1.3.6 Software Stack and Library Versions

All statistical computations were performed in Python 3.11 using widely-used open-source libraries, with no in-house statistical kernels. Specifically: Wilson and Clopper–Pearson confidence intervals, McNemar's chi-square test, Friedman's repeated-measures test, and Spearman's rank correlation were obtained from `**scipy.stats**` (SciPy 1.13); two-way mixed-effects ICC(2,1) and average-rater ICC(3,k) were computed from the Shrout & Fleiss (1979) formulas using `scipy.stats` distributional support, and bias-corrected accelerated (BCa) bootstrap intervals likewise used `scipy.stats` for the bias and acceleration estimates. Multiple-comparison corrections (Holm, Šidák, Benjamini–Hochberg, Benjamini–Yekutieli) were computed via `**statsmodels.stats.multitest.multipletests**` (statsmodels 0.14). Cohen's κ and quadratic-weighted κ, Matthews' correlation, and balanced accuracy were computed via `**sklearn.metrics.cohen_kappa_score**`, `**matthews_corrcoef**`, and `**balanced_accuracy_score**` (scikit-learn 1.5). Data manipulation used pandas 2.x and NumPy 1.26.

Supplementary Section S1.4 — Decoding Parameters, Model-Agnosticism Rationale, and Hardware Envelope (Extended Methods)

This file collects implementation detail that the v10 manuscript carried inline in Section 2.3 but that crowds the main-body methods. Specifically: per-organ DSPy module enumeration, the model-agnosticism rationale for Qwen3-30B-A3B and Gemma 3 27B comparison, and the hardware envelope constraint. The Section 3.1 results section reports the pilot model-selection result (Table 3 — gpt-oss-20b at 94.30 % under the single-run v1-working-gold pilot, with 2–3× faster inference than the alternatives; explicitly framed as the screening study that selected the production engine, not as the headline accuracy); the main-body Section 2.3 carries the one-sentence summary and pointer; the full rationale lives here.

Section S1.4.1 Per-Organ DSPy Module Enumeration

Ten sets of organ-specific DSPy modules were used for organ-specific extraction. For example, the breast cancer pipeline included `BreastCancerNonnested`, `BreastCancerMargins`, `BreastCancerLN`, and `BreastCancerBiomarkers`. Each module receives both the original pathology report and the JSON summary previously generated by gpt-oss-20b as input and outputs validated JSON matching its respective schema. The full per-organ module enumeration (and the universal `EligibilitySignature` + `CancerTypeSignature` cascade-gating modules) can be found in the codes. The modules extract tumor characteristics, AJCC/TNM staging, surgical margins, and lymph-node details for each cancer type; for breast and colorectal cancer, biomarker status is also extracted. Readers are referred to Supplementary Appendix A1–A7 for examples of model output on representative cases.

Section S1.4.2 Model-Agnosticism Validation Rationale

To validate the architectural independence of the extraction framework and assess the feasibility of deployment on standard workstation hardware, the identical pipeline structure was instantiated and executed using two alternative open-weight transformer architectures: **Qwen3-30B-A3B** (sparse mixture-of-experts) and **Gemma 3 27B** (dense). The foundational design of the AI pipeline explicitly embraces principles of model agnosticism, utilizing the DSPy 2.1 framework to decouple the extraction logic from specific model weights.

The performance gap reported in the Section 3.1 pilot (Table 3 — gpt-oss-20b at 94.30 % vs Qwen3-30B-A3B 92.90 % and Gemma 3 27B 89.80 % on a single run against the v1 working gold) is not primarily due to simple VRAM capacity exhaustion; rather, it reflects the interplay of model architecture, total parameter state, expert routing overhead (in the case of mixture-of-experts (MoE) models), and memory/bandwidth traffic. For instance, Qwen3-30B-A3B, despite activating only ~3.3 billion parameters per token, has ~30.5 billion total parameters and uses 128 experts with 8 activated per token. These aspects increase system overhead and latency, positioning it behind gpt-oss-20b in our single-GPU workstation tests.

Section S1.4.3 Hardware Envelope

All comparative models were executed on-premises using the same dedicated NVIDIA RTX A6000 Ada Generation GPU (48 GB VRAM). This hardware constraint was strictly enforced to evaluate whether models could operate efficiently within the memory envelope of a standard high-end medical workstation, without requiring multi-GPU datacenter configurations.

Section S1.4.4 Decoding Parameters

Decoding parameters across all gpt-oss-20b inference passes are pinned by the project's `MODEL_PROFILES` config (committed alongside the analysis code). All inference uses grammar-constrained sampling via the Ollama + llama.cpp GBNF route described in Section S1.2.4. The DSPy adapter compiles each `dspy.Signature` declaration into a JSON Schema, which Ollama converts to a GBNF grammar that masks tokens violating the schema at sample time; this guarantees that the generated tokens are always parseable as the typed nested JSON record the per-organ module declares. Within these grammar-bounded sample distributions, the decoding hyperparameters (temperature, top_p, num_ctx) are unchanged across the 30 multi-seed runs; only the random seed varies.

Supplementary Tables S1–S64

Supplementary Table S1. Field definitions and constraints for the Breast Cancer schema.

This table defines every variable that the per-organ extractor is asked to predict for breast-cancer surgical-excision pathology reports. The schema is enforced as a strongly-typed Python dataclass (`Literal`, `bool`, `int`, or `None`) at generation time via DSPy + the `cancer_data.breast` Pydantic model. Reproduced verbatim from the original docx; this is the schema the model was trained against and the schema all Section 3.3 per-field accuracy numbers (Supplementary Table S14, S27) score against.

Field name	Description / Clinical meaning	Data type	Allowed values or enumeration	Nullable / Optional
procedure	Surgical procedure	Literal[str]	partial_mastectomy, breast_conserving_surgery, modified_radical_mastectomy, total_mastectomy, others	No
histological_type	Histologic diagnosis of primary tumour	Literal[str]	invasive_carcinoma_no_special_type, invasive_lobular_carcinoma, mixed_ductal_and_lobular_carcinoma, tubular_adenocarcinoma, mucinous_adenocarcinoma, medullary_carcinoma, papillary_carcinoma, metaplastic_carcinoma, apocrine_carcinoma, encapsulated_papillary_carcinoma, solid_papillary_carcinoma, inflammatory_carcinoma, other_special_types	No
cancer_quadrant	Tumor position as defined by quadrants	Literal[str]	upper_outer_quadrant, upper_inner_quadrant, lower_outer_quadrant, lower_inner_quadrant, nipple, others	Yes
cancer_clock	Tumor position as defined by clock position	Literal[int]	1, 2, 3, 4, 5, 6, 7, 8, 9, 10, 11, 12	Yes
cancer_laterality	Tumor laterality	Literal[str]	right, left	No
tumor_size	Maximum tumour dimension in millimetres	int	unlimited	Yes
lymphovascular_invasion	Presence of lymphatic or	bool	True, False	Yes

	vascular invasion			
perineural_invasion	Presence of perineural invasion	bool	True, False	Yes
distant_metastasis	Clinical or pathologic evidence of distant metastasis	bool	True, False	No
dcis_present	Whether ductal carcinoma in situ is present in specimen	bool	True, False	No
dcis_size	If ductal carcinoma in situ is present in specimen, size in millimeters	int	Any integer	Yes
dcis_grade	If ductal carcinoma in situ is present, identify the grade of DCIS (low=1, intermediate=2, high=3)	Literal[int]	1, 2, 3	Yes
tnm_descriptor	TNM descriptor for tumor staging	Literal[str]	y, r, m	Yes
pt_category	Pathologic T staging	Literal[str]	tx, tis, t1mi, t1a, t1b, t1c, t2, t3, t4a, t4b, t4c	Yes
pn_category	Pathologic N staging	Literal[str]	nx, n0, n1mi, n1a, n1b, n1c, n2a, n2b, n3a, n3b, n3c	Yes
pm_category	Pathologic M staging	Literal[str]	mx, m0, m1a, m1b, m1c	Yes
pathologic_stage_group	Pathologic stage group	Literal[str]	0, ia, ib, iia, iib, iiia, iiib, iiic, iv	Yes
anatomic_stage_group	Anatomic stage group	Literal[str]	0, ia, ib, iia, iib, iiia, iiib, iiic, iv	Yes
nuclear_grade	Nuclear grading component of Nottingham grading	Literal[int]	1, 2, 3	Yes

tubule_formation	Tubule component of Nottingham grading	Literal[int]	1, 2, 3	Yes
mitotic_rate	Mitotic-rate component of Nottingham grading	Literal[int]	1, 2, 3	Yes
total_score	Total grading score of Nottingham grading	Literal[int]	3, 4, 5, 6, 7, 8, 9	Yes
grade	Overall Nottingham grade	Literal[int]	1, 2, 3	Yes
extranodal_extension	Presence of extranodal extension, if there is lymph node metastasis	bool	True, False	Yes
maximal_ln_size	Maximal size of node metastatic tumour in mm, rounded to integer (if LN metastasis)	int	unlimited	Yes
lymph_node_category	Standard group/station for lymph node dissection	Literal[str]	sentinel, nonsentinel, others	Yes
margin_category	Standard margin category for breast excision	Literal[str]	12_3_clock, 3_6_clock, 6_9_clock, 9_12_clock, 12_clock, 3_clock, 6_clock, 9_clock, superficial, base, others	Yes

Supplementary Table S2. Field definitions and constraints for the Lung Cancer schema.

This table defines every variable that the per-organ extractor is asked to predict for lung-cancer surgical-excision pathology reports. Schema enforced as a strongly-typed Python dataclass at generation time. Reproduced verbatim from the original docx; this is the schema all Section 3.3 per-field accuracy numbers (Supplementary Tables S19, S27) score against.

Field name	Description / Clinical meaning	Data type	Allowed values or enumeration	Nullable / Optional
procedure	Surgical procedure	Literal[str]	wedge_resection, segmentectomy, lobetomy, completion_lobectomy, sleeve_lovectomy, bilobectomy, pneumonectomy, major_airway_resection, others	No
surgical_technique	How the surgery is done	Literal[str]	open, thoracoscopic, robotic, hybrid	No
cancer_laterality	Tumor laterality	Literal[str]	right, left	No
cancer_primary_site	Tumor location	Literal[str]	upper_lobe, middle_lobe, lower_lobe, main_bronchus, others	No
tumor_focality	Whether the tumour has metastasized to other lobe or same lobe	Literal[str]	single_focus, separate_in_same_lobe_t3, separate_nodule_in_ipsilateral_t4, separate_nodule_in_contralateral_m1a	No
histology	Histological type of the tumour	Literal[str]	adenocarcinoma, squamous_cell_carcinoma, adenosquamous_carcinoma, large_cell_carcinoma, pleomorphic_carcinoma, others	No
grade	Tumor grading	Literal[int]	1, 2, 3, 4 (well-differentiated, moderatedly-differentiated, poorly-differentiated, undifferentiated)	Yes
lymphovascular_invasion	Presence of lymphatic or vascular invasion	bool	True, False	Yes

perineural_invasion	Presence of perineural invasion	bool	True, False	Yes
distant_metastasis	Clinical or pathologic evidence of distant metastasis	bool	True, False	No
spread_through_air_spaces_stas	Whether or not spread through air spaces (STAS) is present	bool	True, False	Yes
visceral_pleural_invasion	Whether or not visceral pleural invasion is present	bool	True, False	Yes
direct_invasion_of_adjacent_structures	Whether or not direct invasion of adjacent structures is present	bool	True, False	Yes
tnm_descriptor	TNM descriptor for tumor staging	Literal[str]	y, r, m	Yes
pt_category	Pathologic T staging	Literal[str]	tx, tis, t1mi, t1a, t1b, t1c, t2a, t2b, t3, t4	Yes
pn_category	Pathologic N staging	Literal[str]	nx, n0, n1, n2, n3	Yes
pm_category	Pathologic M staging	Literal[str]	mx, m0, m1a, m1b, m1c	Yes
stage_group	Pathologic stage group	Literal[str]	0, ia1, ia2, ia3, ib, iia, iib, iiia, iiib, iiic, iva, ivb, ivc	Yes
extranodal_extension	Presence of extranodal extension, if there is lymph node metastasis	bool	True, False	Yes
maximal_ln_size	Maximal size of node metastatic	int	unlimited	Yes

	tumour in mm, rounded to integer (if LN metastasis)			
margin_category	Standard margin category for lung cancer excision	Literal[str]	bronchial, vascular, parenchymal, chest_wall, others	Yes
lymph_node_category	Standard group/station for lymph node dissection	Literal[str]	peribronchial, 1, 2, 4, 5, 6, 8, 9, 10, 11, 12, 13, 14, 3a, 3p, 7, others	Yes
histological_patterns	Histological patterns and their percentage of adenocarcinoma	List[Literal[str], int]	acinar, papillary, micropapillary, solid, lepidic	Yes

Supplementary Table S3. Field definitions and constraints for the Colorectal Cancer schema.

This table defines every variable that the per-organ extractor is asked to predict for colorectal-cancer surgical-excision pathology reports. Reproduced verbatim from the original docx; this is the schema all Section 3.3 per-field accuracy numbers (Supplementary Tables S16, S27) score against.

Field name	Description / Clinical meaning	Data type	Allowed values or enumeration	Nullable / Optional
procedure	Surgical procedure	Literal[str]	right_hemicolectomy, extended_right_hemicolectomy, left_hemicolectomy, low_anterior_resection, anterior_resection, abdominoperineal_resection, total_mesorectal_excision, total_colectomy, subtotal_colectomy, segmental_colectomy, transanal_local_excision, polypectomy, others	No
surgical_technique	How the surgery is done	Literal[str]	open, laparoscopic, robotic, ta_tme(total mesorectal excision), hybrid, others	No
cancer_primary_site	Tumor location	Literal[str]	cecum, ascending_colon, hepatic_flexure, transverse_colon, splenic_flexure, descending_colon, sigmoid_colon, rectosigmoid_junction, rectum, appendix	No
histology	Histological type of the tumour	Literal[str]	adenocarcinoma, mucinous_adenocarcinoma, signet_ring_cell_carcinoma, medullary_carcinoma, micropapillary_adenocarcinoma, serrated_adenocarcinoma, adenosquamous_carcinoma, neuroendocrine_carcinoma, others	No
grade	Tumor grading	int	1, 2, 3, 4 (well-differentiated, moderatedly-differentiated, poorly-differentiated, undifferentiated)	Yes

tumor_invasion	Invasion depth of the tumour into the colonic wall	Literal[str]	lamina_propria, submucosa, muscularis_propria, pericolorectal_tissue, visceral_peritoneum_surface, adjacent_organs_structures	No
lymphovascular_invasion	Presence of lymphatic or vascular invasion	bool	True, False	Yes
perineural_invasion	Presence of perineural invasion	bool	True, False	Yes
extracellular_mucin	Presence of extracellular mucin secretion	bool	True, False	Yes
signet_ring	Presence of signet ring differentiation	bool	True, False	Yes
tumor_budding	Tumor budding score	int	0, 1, 2 (low, moderate, high)	Yes
type_of_polyp	Type of polyp from which the tumour arises (if present)	Literal[str]	tubular_adenoma, tubulovillous_adenoma, villous_adenoma, sessile_serrated_adenoma, traditional_serrated_adenoma	Yes
distant_metastasis	Clinical or pathologic evidence of distant metastasis	bool	True, False	No
tnm_descriptor	TNM descriptor for tumor staging	Literal[str]	y, r, m	Yes
pt_category	Pathologic T staging	Literal[str]	tx, tis, t1, t2, t3, t4a, t4b	Yes
pn_category	Pathologic N staging	Literal[str]	nx, n0, n1a, n1b, n1c, n2a, n2b	Yes
pm_category	Pathologic M staging	Literal[str]	mx, m0, m1a, m1b, m1c	Yes
stage_group	Pathologic stage group	Literal[str]	0, i, iia, iib, iic, iiia, iiib, iiic, iva, ivb, ivc	Yes
extranodal_extension	Presence of extranodal extension, if	bool	True, False	Yes

	there is lymph node metastasis			
maximal_ln_size	Maximal size of node metastatic tumour in mm, rounded to integer (if LN metastasis)	int	unlimited	Yes
margin_category	Standard margin category for colorectal cancer excision	Literal[str]	proximal, distal, mesenteric_pedicle, radial_or_circumferencial, outmost_of_adhered_tissue, others	Yes
lymph_node_category	Standard group/station for lymph node dissection	Literal[str]	regional, mesenteric, others	Yes

Supplementary Table S4. Field definitions and constraints for the Esophageal Cancer schema.

This table defines every variable that the per-organ extractor is asked to predict for esophageal-cancer surgical-excision pathology reports. Reproduced verbatim from the original docx; this is the schema all Section 3.3 per-field accuracy numbers (Supplementary Tables S17, S27) score against.

Field name	Description / Clinical meaning	Data type	Allowed values or enumeration	Nullable / Optional
procedure	Surgical procedure	Literal[str]	endoscopic_resection, esophagectomy, esophagogastrectomy, others	No
surgical_technique	How the surgery is done	Literal[str]	open, thoracoscopic, robotic, hybrid, endoscopic, others	No
cancer_primary_site	Tumor location	Literal[str]	upper_third, middle_third, lower_third, gastroesophageal_junction	No
histology	Histological type of the tumour	Literal[str]	squamous_cell_carcinoma, adenocarcinoma, adenoid_cystic_carcinoma, mucoepidermoid_carcinoma, basaloid_squamous_cell_carcinoma, small_cell_carcinoma, large_cell_carcinoma, others	No
grade	Tumor grading	int	1, 2, 3 (well-differentiated, moderatedly-differentiated, poorly-differentiated)	Yes
tumor_extent	Invasion depth of the tumour into the esophageal wall	Literal[str]	mucosa, submucosa, muscularis_propria, adventitia, adjacent_structures	No
lymphovascular_invasion	Presence of lymphatic or vascular invasion	bool	True, False	Yes
perineural_invasion	Presence of perineural invasion	bool	True, False	Yes
distant_metastasis	Clinical or pathologic evidence of distant metastasis	bool	True, False	No

tnm_descriptor	TNM descriptor for tumor staging	Literal[str]	y, r, m	Yes
pt_category	Pathologic T staging	Literal[str]	tx, t1a, t1b, t2, t3, t4a, t4b	Yes
pn_category	Pathologic N staging	Literal[str]	nx, n0, n1, n2, n3	Yes
pm_category	Pathologic M staging	Literal[str]	mx, m0, m1	Yes
stage_group	Pathologic stage group	Literal[str]	0, i, ia, ib, ic, iia, iib, iiia, iiib, iva, ivb	Yes
extranodal_extension	Presence of extranodal extension, if there is lymph node metastasis	bool	True, False	Yes
maximal_ln_size	Maximal size of node metastatic tumour in mm, rounded to integer (if LN metastasis)	int	unlimited	Yes
margin_category	Standard margin category for esophageal cancer excision	Literal[str]	proximal, distal, radial, lateral, deep, others	Yes
lymph_node_category	Standard group/station for lymph node dissection	Literal[str]	regional_esophageal, regional_gastric, thoracic_1, thoracic_1r, thoracic_1l, thoracic_4, thoracic_4r, thoracic_4l, thoracic_7, thoracic_8u, thoracic_8m, thoracic_8l, thoracic_8, thoracic_9, thoracic_9r, thoracic_9l, thoracic_10, thoracic_10r, thoracic_10l, abdomen_106, abdomen_1, abdomen_2, abdomen_3, abdomen_4, abdomen_5, abdomen_6, abdomen_7, abdomen_8, abdomen_9, abdomen_10, others	Yes

Supplementary Table S5. Field definitions and constraints for the Gastric Cancer schema.

This table defines every variable that the per-organ extractor is asked to predict for gastric-cancer surgical-excision pathology reports. Reproduced verbatim from the original docx; this is the schema all Section 3.3 per-field accuracy numbers (Supplementary Tables S22, S27) score against.

Field name	Description / Clinical meaning	Data type	Allowed values or enumeration	Nullable / Optional
procedure	Surgical procedure	Literal[str]	endoscopic_resection, partial_gastrectomy, total_gastrectomy, others	No
surgical_technique	How the surgery is done	Literal[str]	open, laparoscopic, robotic, hybrid, others	No
tumor_site	Tumor location	Literal[str]	cardia, fundus, body, antrum, pylorus, others	No
histology	Histological type of the tumour	Literal[str]	tubular_adenocarcinoma, poorly_cohesive_carcinoma, mixed_tubular_poorly_cohesive, mucinous_adenocarcinoma, mixed_mucinous_poorly_cohesive, hepatoid_carcinoma, others	No
grade	Tumor grading	int	1, 2, 3 (well-differentiated, moderatedly-differentiated, poorly-differentiated)	Yes
tumor_extent	Invasion depth of the tumour into the gastric wall	Literal[str]	lamina_propria, muscularis_mucosae, submucosa, muscularis_propria, penetrate_subserosal_connective_tissue_no_serosa, invades_serosa_without_adjacent_structure_invasion, invades_adjacent_structures	No
extracellular_mucin	Presence of extracellular mucin secretion	bool	True, False	Yes
signet_ring	Presence of signet ring differentiation	bool	True, False	Yes
lymphovascular_invasion	Presence of lymphatic or vascular invasion	bool	True, False	Yes

perineural_invasion	Presence of perineural invasion	bool	True, False	Yes
distant_metastasis	Clinical or pathologic evidence of distant metastasis	bool	True, False	No
tnm_descriptor	TNM descriptor for tumor staging	Literal[str]	y, r, m	Yes
pt_category	Pathologic T staging	Literal[str]	tx, t1a, t1b, t2, t3, t4a, t4b	Yes
pn_category	Pathologic N staging	Literal[str]	nx, n0, n1, n2, n3a, n3b	Yes
pm_category	Pathologic M staging	Literal[str]	mx, m0, m1	Yes
stage_group	Pathologic stage group	Literal[str]	0, i, ia, ib, ii, iia, iib, iii, iiia, iiib, iiic, iv	Yes
extranodal_extension	Presence of extranodal extension, if there is lymph node metastasis	bool	True, False	Yes
maximal_ln_size	Maximal size of node metastatic tumour in mm, rounded to integer (if LN metastasis)	int	unlimited	Yes
margin_category	Standard margin category for gastric cancer excision	Literal[str]	proximal, distal, radial, lateral, deep, others	Yes
lymph_node_category	Standard group/station for lymph	Literal[str]	regional, regional_lesser_curv, regional_greater_curv, 1, 2, 3, 4, 5, 6, 7, 8, 9, 10, 11, others	Yes

	node dissection			

Supplementary Table S6. Field definitions and constraints for the Liver Cancer (hepatocellular carcinoma) schema.

This table defines every variable that the per-organ extractor is asked to predict for hepatocellular-carcinoma surgical-excision pathology reports. Reproduced verbatim from the original docx; this is the schema all Section 3.3 per-field accuracy numbers (Supplementary Tables S18, S27) score against.

Field name	Description / Clinical meaning	Data type	Allowed values or enumeration	Nullable / Optional
procedure	Surgical procedure	Literal[str]	wedge_resection, partial_hepatectomy, segmentectomy, lobectomy, total_hepatectomy, others	No
cancer_primary_site	Tumor location	Literal[str]	right_lobe, left_lobe, caudate_lobe, quadrate_lobe, others	No
histology	Histological type of the tumour	Literal[str]	hepatocellular_carcinoma, hepatocellular_carcinoma_fibrolamellar, hepatocellular_carcinoma_scirrhous, hepatocellular_carcinoma_clear_cell, others	No
grade	Tumor grading	int	1, 2, 3, 4 (well-differentiated, moderatedly-differentiated, poorly-differentiated, undifferentiated)	Yes
tumor_size	Maximum tumour dimension in millimetres	int	unlimited	Yes
tumor_focality	Whether the tumour is unifocal or multifocal	Literal[str]	unifocal, multifocal	No
vascular_invasion	List of vascular invasion sites if present	List[Literal[str]]	large_hepatic_vein, large_portal_vein, small_vessel	Yes
perineural_invasion	Presence of perineural invasion	bool	True, False	Yes
distant_metastasis	Clinical or pathologic evidence of distant metastasis	bool	True, False	No

tnm_descriptor	TNM descriptor for tumor staging	Literal[str]	y, r, m	Yes
pt_category	Pathologic T staging	Literal[str]	tx, t1a, t1b, t2, t3, t4	Yes
pn_category	Pathologic N staging	Literal[str]	nx, n0, n1	Yes
pm_category	Pathologic M staging	Literal[str]	mx, m0, m1	Yes
overall_stage	Pathologic stage group	Literal[str]	ia, ib, ii, iiia, iiib, iva, ivb	Yes
extranodal_extension	Presence of extranodal extension, if there is lymph node metastasis	bool	True, False	Yes
maximal_ln_size	Maximal size of node metastatic tumour in mm, rounded to integer (if LN metastasis)	int	unlimited	Yes
margin_category	Standard margin category for hepatocellular carcinoma excision	Literal[str]	parenchymal, hepatic_vein, portal_vein, bile_duct, others	Yes
lymph_node_category	Standard group/station for lymph node dissection	None	(No standard group/station names for liver resection specimen.)	Yes

Supplementary Table S7. Field definitions and constraints for the Pancreas Cancer schema.

This table defines every variable that the per-organ extractor is asked to predict for pancreatic-cancer surgical-excision pathology reports. Reproduced verbatim from the original docx; this is the schema all Section 3.3 per-field accuracy numbers (Supplementary Tables S20, S27) score against.

Field name	Description / Clinical meaning	Data type	Allowed values or enumeration	Nullable / Optional
procedure	Surgical procedure	Literal[str]	partial_pancreatectomy, ssppd, pppd, whipple_procedure, distal_pancreatectomy, total_pancreatectomy, others	No
tumor_site	Tumor location	Literal[str]	head, neck, body, tail, uncinate_process, others	No
histology	Histological type of the tumour	Literal[str]	ductal_adenocarcinoma_nos, ipmn_with_carcinoma, itpn_with_carcinoma, acinar_cell_carcinoma, solid_pseudopapillary_neoplasm, undifferentiated_carcinoma, others	No
tumor_size	Maximum tumour dimension in millimetres	int	unlimited	Yes
tumor_extension	Tumor extent	Literal[str]	within_pancreas, peripancreatic_soft_tissue, adjacent_organs_structures, others	No
lymphovascular_invasion	Presence of lymphatic or vascular invasion	bool	True, False	Yes
perineural_invasion	Presence of perineural invasion	bool	True, False	Yes
distant_metastasis	Clinical or pathologic evidence of distant metastasis	bool	True, False	No

tnm_descriptor	TNM descriptor for tumor staging	Literal[str]	y, r, m	Yes
pt_category	Pathologic T staging	Literal[str]	tx, tis, t1a, t1b, t1c, t2, t3, t4	Yes
pn_category	Pathologic N staging	Literal[str]	nx, n0, n1, n2	Yes
pm_category	Pathologic M staging	Literal[str]	mx, m0, m1	Yes
overall_stage	Pathologic stage group	Literal[str]	ia, ib, ii, iiia, iiib, iva, ivb	Yes
extranodal_extension	Presence of extranodal extension, if there is lymph node metastasis	bool	True, False	Yes
maximal_ln_size	Maximal size of node metastatic tumour in mm, rounded to integer (if LN metastasis)	int	unlimited	Yes
margin_category	Standard margin category for pancreas cancer excision	Literal[str]	parenchymal, hepatic_vein, portal_vein, bile_duct, others	Yes
lymph_node_category	Standard group/station for lymph node dissection	None	(No standard group/station names — original docx description matches the liver schema; this is a known docx copy-paste artifact, see NOTE.)	Yes

Supplementary Table S8. Field definitions and constraints for the Prostate Cancer schema.

This table defines every variable that the per-organ extractor is asked to predict for prostatic-cancer surgical-excision pathology reports. Reproduced verbatim from the original docx; this is the schema all Section 3.3 / Section 3.4 per-field accuracy numbers (Supplementary Tables S21, S27, S33) score against. Note that the prostate schema is the only organ with a binary `margin_positivity` flag plus a list-typed `involved_margin_list` (12 named directions); the `margins[*]` list-typed field used in the other nine organs is replaced here by this two-field design (see Supplementary Table S33 for the resulting evaluation flow).

Field name	Description / Clinical meaning	Data type	Allowed values or enumeration	Nullable / Optional
procedure	Surgical procedure	Literal[str]	radical_prostatectomy, others	No
surgical_technique	How the surgery is done	Literal[str]	open, robotic, hybrid, others	No
prostate_size	Prostate size in millimetres	int	unlimited	Yes
prostate_weight	Prostate weight in grams	int	unlimited	Yes
histology	Histological type of the tumour	Literal[str]	acinar_adenocarcinoma, intraductal_carcinoma, ductal_adenocarcinoma, mixed_acinar_ductal, neuroendocrine_carcinoma_small_cell, others	No
grade	Gleason grade group and pattern (e.g., "grade group 1, Gleason grade 6 (3+3)")	Literal[str]	group_1_3_3, group_2_3_4, group_3_4_3, group_4_4_4, group_5_4_5, group_5_5_4, group_5_5_5	Yes
gleason_4_percentage	Percentage of Gleason pattern 4	int	0–100	Yes
gleason_5_percentage	Percentage of Gleason pattern 5	int	0–100	Yes

intraductal_carcinoma_presence	Whether intraductal carcinoma is present in specimen	bool	True, False	Yes
cribriform_pattern_presence	Whether the tumour exhibits cribriform pattern	bool	True, False	Yes
tumor_percentage	Percentage of tumour area in both lobes	int	0–100	Yes
tumor_size	Size of tumour in mm	int	unlimited	Yes
extraprostatic_extension	Presence of extraprostatic extension	bool	True, False	Yes
seminal_vesicle_invasion	Presence of seminal vesicle invasion	bool	True, False	Yes
bladder_invasion	Presence of urinary bladder invasion	bool	True, False	Yes
lymphovascular_invasion	Presence of lymphatic or vascular invasion	bool	True, False	Yes
perineural_invasion	Presence of perineural invasion	bool	True, False	Yes
distant_metastasis	Clinical or pathologic evidence of distant metastasis	bool	True, False	No
tnm_descriptor	TNM descriptor for tumor staging	Literal[str]	y, r, m	Yes

pt_category	Pathologic T staging	Literal[str]	tx, t2, t3a, t3b, t4	Yes
pn_category	Pathologic N staging	Literal[str]	nx, n0, n1	Yes
pm_category	Pathologic M staging	Literal[str]	mx, m0, m1a, m1b, m1c	Yes
overall_stage	Pathologic stage group	Literal[str]	ia, ib, ii, iiia, iiib, iva, ivb	Yes
extranodal_extension	Presence of extranodal extension, if there is lymph node metastasis	bool	True, False	Yes
maximal_ln_size	Maximal size of node metastatic tumour in mm, rounded to integer (if LN metastasis)	int	unlimited	Yes
margin_positivity	Whether outmost resection margin of prostate is involved by tumour	bool	True, False	No
involved_margin_list	List of margin directions involved by tumour (if margin positive)	List[Literal[str]]	right_apical, left_apical, right_bladder_neck, left_bladder_neck, right_anterior, left_anterior, right_lateral, left_lateral, right_posterolateral, left_posterolateral, right_posterior, left_posterior	Yes
lymph_node_category	Standard group/station for lymph node dissection	Literal[str]	hypogastric, obturator, external_iliac, internal_iliac, common_iliac, iliac_nos, pelvic_nos, others	Yes

Supplementary Table S9. Field definitions and constraints for the Uterine Cervix Cancer schema.

This table defines every variable that the per-organ extractor is asked to predict for uterine-cervix cancer surgical-excision pathology reports. Reproduced verbatim from the original docx; this is the schema all Section 3.3 per-field accuracy numbers (Supplementary Tables S15, S27) score against.

Field name	Description / Clinical meaning	Data type	Allowed values or enumeration	Nullable / Optional
procedure	Surgical procedure	Literal[str]	partial_pancreatectomy, ssppd, pppd, whipple_procedure, distal_pancreatectomy, total_pancreatectomy, others	No
surgical_technique	How the surgery is done	Literal[str]	open, laparoscopic, vaginal, others	No
cancer_primary_site	Tumor location	Literal[str]	12_3_clock, 3_6_clock, 6_9_clock, 9_12_clock	No
histology	Histological type of the tumour	Literal[str]	squamous_cell_carcinoma_hpv_associated, squamous_cell_carcinoma_hpv_dependaent, squamous_cell_carcinoma_nos, adenocarcinoma_hpv_associated, adenocarcinoma_hpv_independent, adenocarcinoma_nos, adenosquamous_carcinoma, neuroendocrine_carcinoma, glassy_cell_carcinoma, small_cell_carcinoma, large_cell_carcinoma, others	No
grade	Tumor grading	int	1, 2, 3 (well-differentiated, moderately-differentiated, poorly-differentiated)	Yes
tumor_size	Maximum tumour dimension in millimetres	int	unlimited	Yes
depth_of_invasion_number	Depth of invasion, expressed in three categories according to absolute depth: <3	Literal[str]	less_than_3, 3_to_5, greater_than_5	No

	mm, 3–5 mm and >5 mm			
depth_of_invasion_three_tier	Depth of invasion expressed in three tiers of cervical stromal involvement: inner third, middle third, outer third	Literal[str]	Inner_third, middle_third, outer_third	No
lymphovascular_invasion	Presence of lymphatic or vascular invasion	bool	True, False	Yes
perineural_invasion	Presence of perineural invasion	bool	True, False	Yes
distant_metastasis	Clinical or pathologic evidence of distant metastasis	bool	True, False	No
tnm_descriptor	TNM descriptor for tumor staging	Literal[str]	y, r, m	Yes
pt_category	Pathologic T staging	Literal[str]	tx, t1a1, t1a2, t1b1, t1b2, t1b3, t2a1, t2a2, t2b, t3a, t3b, t4	Yes
pn_category	Pathologic N staging	Literal[str]	nx, n0, n1mi, n1a, n2mi, n2a	Yes
pm_category	Pathologic M staging	Literal[str]	mx, m0, m1	Yes
stage_group	FIGO stage group	Literal[str]	0, ia1, ia2, ib1, ib2, ib3, iia1, iia2, iib, iiia, iiib, iiic1, iiic2, iva, ivb	Yes
extranodal_extension	Presence of extranodal extension, if there is lymph node metastasis	bool	True, False	Yes
maximal_ln_size	Maximal size of node	int	unlimited	Yes

	metastatic tumour in mm, rounded to integer (if LN metastasis)			
margin_category	Standard margin category for cervix cancer excision (original docx: "pancreas cancer excision" — copy-paste artifact)	Literal[str]	ectocervical, endocervical, radial_circumferential, vaginal_cuff, others	Yes
lymph_node_category	Standard group/station for lymph node dissection	Literal[str]	pelvic, para_aortic, internal_iliac, obturator, external_iliac, common_iliac, parametrial, others	Yes

Supplementary Table S10. Field definitions and constraints for the Thyroid Cancer schema.

This table defines every variable that the per-organ extractor is asked to predict for thyroid cancer surgical-excision pathology reports. Reproduced verbatim from the original docx; this is the schema all Section 3.3 per-field accuracy numbers (Supplementary Tables S23, S27) score against.

Field name	Description / Clinical meaning	Data type	Allowed values or enumeration	Nullable / Optional
predisposing_condition	Whether the patient had previous radiation exposure or a family history of thyroid cancer	Literal[str]	radiation, family_history	Yes
procedure	Surgical procedure	Literal[str]	partial_excision, right_lobectomy, left_lobectomy, total_thyroidectomy, others	No
tumor_focality	Whether the tumour is unifocal or multifocal	Literal[str]	unifocal, multifocal, not_specified	(blank in docx)
tumor_site	Tumor location (original docx: pancreas tumour-site enum — copy-paste artifact)	Literal[str]	head, neck, body, tail, uncinate_process, others	No
histology	Histological type of the tumour (original docx: pancreas histology enum — copy-paste artifact)	Literal[str]	ductal_adenocarcinoma_nos, ipmn_with_carcinoma, itpn_with_carcinoma, acinar_cell_carcinoma, solid_pseudopapillary_neoplasm, undifferentiated_carcinoma, others	No
tumor_size	Maximum tumour dimension in millimetres	int	unlimited	Yes
tumor_extension	Tumor extent (original docx: pancreas	Literal[str]	within_pancreas, peripancreatic_soft_tissue,	No

	extension enum — copy-paste artifact)		adjacent_organs_structures, others	
lymphovascular_invasion	Presence of lymphatic or vascular invasion	bool	True, False	Yes
perineural_invasion	Presence of perineural invasion	bool	True, False	Yes
distant_metastasis	Clinical or pathologic evidence of distant metastasis	bool	True, False	No
tnm_descriptor	TNM descriptor for tumor staging	Literal[str]	y, r, m	Yes
pt_category	Pathologic T staging	Literal[str]	tx, tis, t1a, t1b, t1c, t2, t3, t4	Yes
pn_category	Pathologic N staging	Literal[str]	nx, n0, n1, n2	Yes
pm_category	Pathologic M staging	Literal[str]	mx, m0, m1	Yes
overall_stage	Pathologic stage group	Literal[str]	ia, ib, ii, iiia, iiib, iva, ivb	Yes
extranodal_extension	Presence of extranodal extension, if there is lymph node metastasis	bool	True, False	Yes
maximal_ln_size	Maximal size of node metastatic tumour in mm, rounded to integer (if LN metastasis)	int	unlimited	Yes
margin_category	Standard margin category for thyroid cancer excision (original docx: "pancreas cancer excision")	Literal[str]	parenchymal, hepatic_vein, portal_vein, bile_duct, others	Yes

lymph_node_category	Standard group/station for lymph node dissection	None	(No standard group/station names — original docx description matches the liver schema; copy-paste artifact.)	Yes

Supplementary Table S11. 30-run aggregate organ-classification confusion matrix (total counts across all 30 runs).

Twelve-class organ-labeling sub-model confusion across the 30 multi-seed runs of gpt-oss-20b on the CMUH validation cohort. Each cell is the **total count of case-runs** across all 30 runs where the gold class is the row and the predicted class is the column (e.g., `gold = others, pred = lung` = 150 means: across the 30 runs, the model misrouted a gold-`others` case as `lung` 150 times in total). This **replaces** the historical v1 three-run, run-by-run confusion (which had been at S11–S13 in the original docx) with a single aggregate table on the new 30-run pass; per-class precision/recall/F1 is at Supplementary Table S12; per-class run-level stability is at Supplementary Table S13. Backs Section 3.2 + Figure S5.

Row totals: each in-scope row's total = (n_cases_for_class × 30 runs). The `gold-others` row total is 943 ≈ 31.4 × 30 = 943 — the gold-`others` cohort has 31.4 cases per run after Stage-A gating; across 30 runs the total cells are 943. The `(null)` row captures the 30 case-runs where the gold annotation is null but the model still emits a predicted class (essentially all `(null) → lung`).

Gold \\ Pred	pred_breast	pred_cervix	pred_colorectal	pred_esophagus	pred_liver	pred_lung	pred_others	pred_pancreas	pred_prostate	pred_stomach	pred_thyroid	row_total
true_breast	**2,248**	0	0	0	0	0	0	0	0	0	0	2,248
true_cervix	0	**810**	0	0	0	0	0	0	0	0	0	810
true_colorectal	0	0	**2,160**	0	0	0	0	0	0	0	0	2,160
true_esophagus	0	0	0	**2,169**	0	0	4	0	0	17	0	2,190
true_liver	0	0	0	0	**2,400**	0	0	0	0	0	0	2,400
true_lung	0	0	0	0	0	**1,650**	0	0	0	0	0	1,650
true_pancreas	0	0	0	0	0	0	0	**750**	0	0	0	750
true_prostate	0	0	0	0	0	0	0	0	**2,820**	0	0	2,820
true_stomach	0	0	0	25	0	0	5	0	0	**2,340**	0	2,370
true_thyroid	0	0	0	0	0	0	0	0	0	0	**2,160**	2,160
true_others	30	48	60	26	30	150	**431**	108	0	60	0	943
true_(null)	0	0	0	0	0	30	0	0	0	0	0	30

Supplementary Table S12. 30-run aggregate per-class precision, recall, F1, and support for the organ-labeling sub-model.

Per-class classification metrics for the eleven-class organ classifier on the CMUH validation cohort, computed across the 30 multi-seed runs of gpt-oss-20b. **Support** is the total count of case-runs whose gold class is the row (= n_unique_cases × 30 runs). This **replaces** the historical v1 single-run snapshot at S12; it is the canonical per-class summary of the Section 3.2 results and the per-class breakdown underlying the macro-F1 = 0.947 cited at the Section 3.2 headline.

**Label**	**Precision**	**Recall**	**F1**	**Support**
breast	0.9868	1.0000	0.9934	2,248
cervix	0.9441	1.0000	0.9712	810
colorectal	0.9730	1.0000	0.9863	2,160
esophagus	0.9770	0.9904	0.9837	2,190
liver	0.9877	1.0000	0.9938	2,400
lung	0.9167	1.0000	0.9565	1,650
**others**	**0.9795**	**0.4571**	**0.6233**	**943**
pancreas	0.8741	1.0000	0.9328	750
prostate	1.0000	1.0000	1.0000	2,820
stomach	0.9681	0.9873	0.9776	2,370
thyroid	1.0000	1.0000	1.0000	2,160
**macro avg**	0.9643	0.9486	**0.9471**	—
micro avg	0.9725	0.9725	0.9725	—
weighted avg	0.9737	0.9725	0.9684	—

Supplementary Table S13. 30-run per-class recall stability (mean, SD, min, max per gold class).

For each gold organ class, the per-run recall is computed (out of `n_cases_in_class` per run); reported here are the mean, standard deviation, minimum and maximum across the 30 multi-seed runs. This **replaces** the historical v1 single-run snapshot at S13 (which was actually Run 3 with a "Run 2" caption typo); it is the per-class analogue of the Section 3.8 / Supplementary Table S35 reliability statistics, surfaced at the same granularity as the v1 per-run confusions but resolved across the full 30 runs.

Gold class	Mean recall	SD recall	Min recall	Max recall	n_cases_per_run
breast	1.0000	0.0000	1.0000	1.0000	75
cervix	1.0000	0.0000	1.0000	1.0000	27
colorectal	1.0000	0.0000	1.0000	1.0000	72
esophagus	0.9904	0.0082	0.9726	1.0000	73
liver	1.0000	0.0000	1.0000	1.0000	80
lung	1.0000	0.0000	1.0000	1.0000	55
pancreas	1.0000	0.0000	1.0000	1.0000	25
prostate	1.0000	0.0000	1.0000	1.0000	94
stomach	0.9873	0.0088	0.9620	1.0000	79
thyroid	1.0000	0.0000	1.0000	1.0000	72
**others**	**0.4568**	**0.0425**	**0.3871**	**0.5313**	~31.4
(null)	0.0000	0.0000	0.0000	0.0000	1

Supplementary Table S14. Per-field extraction accuracy for breast cancer excision reports (30-run aggregate, 30 schema fields).

Per-field extraction accuracy of the per-organ extractor on the breast subset of the CMUH validation cohort, **30 multi-seed runs of gpt-oss-20b** against the committee-resolved gold standard. n = 2,248 case-runs per field (75 unique reports × ~30 effective runs after Stage-A/B gating). Schema definition: Supplementary Table S1.

Field	n_total	n_attempted	n_correct	Coverage	Effective accuracy [95 % Wilson CI]
anatomic_stage_group	2,248	2,248	2,202	100.00 %	**97.95 % [97.28, 98.46]**
biomarker_er	2,248	2,248	2,218	100.00 %	**98.67 % [98.10, 99.06]**
biomarker_her2	2,248	2,248	1,738	100.00 %	**77.31 % [75.54, 79.00]**
biomarker_ki67	2,248	2,247	1,939	99.96 %	**86.25 % [84.77, 87.62]**
biomarker_pr	2,248	2,248	2,210	100.00 %	**98.31 % [97.69, 98.77]**

cancer_clock	2,248	2,248	1,918	100.00 %	**85.32 % [83.80, 86.72]**
cancer_laterality	2,248	2,248	2,218	100.00 %	**98.67 % [98.10, 99.06]**
cancer_quadrant	2,248	2,248	1,629	100.00 %	**72.46 % [70.58, 74.27]**
dcis_comedo_necrosis	2,248	2,248	2,161	100.00 %	**96.13 % [95.25, 96.85]**
dcis_grade	2,248	2,248	2,189	100.00 %	**97.38 % [96.63, 97.96]**
dcis_present	2,248	2,248	2,181	100.00 %	**97.02 % [96.23, 97.65]**
dcis_size	2,248	2,248	2,206	100.00 %	**98.13 % [97.48, 98.61]**
distant_metastasis	2,248	2,248	544	100.00 %	**24.20 % [22.47, 26.01]**
extranodal_extension	2,248	2,245	2,138	99.87 %	**95.11 % [94.14, 95.92]**
grade	2,248	2,248	2,245	100.00 %	**99.87 % [99.61, 99.95]**
histology	2,248	2,248	2,226	100.00 %	**99.02 % [98.52, 99.35]**
lymphovascular_invasion	2,248	2,248	2,237	100.00 %	**99.51 % [99.13, 99.73]**
maximal_ln_size	2,248	2,245	2,229	99.87 %	**99.15 % [98.68, 99.46]**
mitotic_rate	2,248	2,248	2,248	100.00 %	**100.00 % [99.83, 100.00]**
nuclear_grade	2,248	2,248	2,225	100.00 %	**98.98 % [98.47, 99.32]**
pathologic_stage_group	2,248	2,248	1,683	100.00 %	**74.87 % [73.03, 76.62]**
perineural_invasion	2,248	2,248	1,779	100.00 %	**79.14 % [77.41, 80.77]**
pm_category	2,248	2,248	1,900	100.00 %	**84.52 % [82.97, 85.96]**
pn_category	2,248	2,248	2,233	100.00 %	**99.33 % [98.90, 99.60]**
procedure	2,248	2,248	2,107	100.00 %	**93.73 % [92.65, 94.66]**
pt_category	2,248	2,248	2,247	100.00 %	**99.96 % [99.75, 99.99]**

tnm_descriptor	2,248	2,248	2,060	100.00 %	**91.64 % [90.42, 92.71]**
total_score	2,248	2,248	2,248	100.00 %	**100.00 % [99.83, 100.00]**
tubule_formation	2,248	2,248	2,248	100.00 %	**100.00 % [99.83, 100.00]**
tumor_size	2,248	2,248	2,181	100.00 %	**97.02 % [96.23, 97.65]**

Supplementary Table S15. Per-field extraction accuracy for cervical cancer excision reports (30-run aggregate, 16 schema fields).

Per-field extraction accuracy of the per-organ extractor on the cervix subset of the CMUH validation cohort, **30 multi-seed runs of gpt-oss-20b** against the committee-resolved gold standard. n = 810 case-runs per field (27 unique reports × 30 effective runs). Schema definition: Supplementary Table S9.

Field	n_total	n_attempted	n_correct	Coverage	Effective accuracy [95 % Wilson CI]
cancer_primary_site	810	810	748	100.00 %	**92.35 % [90.31, 93.98]**
depth_of_invasion_number	810	810	810	100.00 %	**100.00 % [99.53, 100.00]**
depth_of_invasion_three_tier	810	810	742	100.00 %	**91.60 % [89.49, 93.32]**
distant_metastasis	810	810	73	100.00 %	**9.01 % [7.23, 11.18]**
extranodal_extension	810	800	768	98.77 %	**94.81 % [93.07, 96.14]**
grade	810	810	810	100.00 %	**100.00 % [99.53, 100.00]**
histology	810	810	785	100.00 %	**96.91 % [95.48, 97.90]**
maximal_ln_size	810	800	800	98.77 %	**98.77 % [97.74, 99.33]**
pm_category	810	810	589	100.00 %	**72.72 % [69.55, 75.67]**
pn_category	810	810	769	100.00 %	**94.94 % [93.21, 96.25]**
procedure	810	810	748	100.00 %	**92.35 % [90.31, 93.98]**
pt_category	810	810	779	100.00 %	**96.17 % [94.62, 97.29]**
stage_group	810	810	783	100.00 %	**96.67 % [95.19, 97.70]**
surgical_technique	810	810	570	100.00 %	**70.37 % [67.14, 73.41]**
tnm_descriptor	810	810	791	100.00 %	**97.65 % [96.37, 98.49]**
tumor_size	810	810	682	100.00 %	**84.20 % [81.52, 86.55]**

Supplementary Table S16. Per-field extraction accuracy for colorectal cancer excision reports (30-run aggregate, 24 schema fields).

Per-field extraction accuracy of the per-organ extractor on the colorectal subset of the CMUH validation cohort, **30 multi-seed runs of gpt-oss-20b** against the committee-resolved gold standard. n = 2,160 case-runs per field (72 unique reports × 30 effective runs). Schema definition: Supplementary Table S3.

Field	n_total	n_attempted	n_correct	Coverage	Effective accuracy [95 % Wilson CI]
biomarker_mlh1	2,160	2,040	2,040	94.44 %	**94.44 % [93.40, 95.33]**
biomarker_msh2	2,160	1,980	1,980	91.67 %	**91.67 % [90.43, 92.76]**
biomarker_msh6	2,160	2,040	2,040	94.44 %	**94.44 % [93.40, 95.33]**
biomarker_pms2	2,160	2,040	2,040	94.44 %	**94.44 % [93.40, 95.33]**
cancer_primary_site	2,160	2,160	2,160	100.00 %	**100.00 % [99.82, 100.00]**
distant_metastasis	2,160	2,160	505	100.00 %	**23.38 % [21.64, 25.21]**
extracellular_mucin	2,160	2,160	2,160	100.00 %	**100.00 % [99.82, 100.00]**
extranodal_extension	2,160	2,160	1,917	100.00 %	**88.75 % [87.35, 90.01]**
grade	2,160	2,160	2,160	100.00 %	**100.00 % [99.82, 100.00]**
histology	2,160	2,160	2,159	100.00 %	**99.95 % [99.74, 99.99]**
lymphovascular_invasion	2,160	2,160	2,160	100.00 %	**100.00 % [99.82, 100.00]**
maximal_ln_size	2,160	2,160	2,160	100.00 %	**100.00 % [99.82, 100.00]**
perineural_invasion	2,160	2,160	2,160	100.00 %	**100.00 % [99.82, 100.00]**
pm_category	2,160	2,160	2,033	100.00 %	**94.12 % [93.05, 95.04]**
pn_category	2,160	2,160	2,092	100.00 %	**96.85 % [96.03, 97.51]**
procedure	2,160	2,160	2,054	100.00 %	**95.09 % [94.10, 95.93]**
pt_category	2,160	2,160	2,149	100.00 %	**99.49 % [99.09, 99.72]**

signet_ring	2,160	2,160	2,157	100.00 %	**99.86 % [99.59, 99.95]**
stage_group	2,160	2,160	2,067	100.00 %	**95.69 % [94.75, 96.47]**
surgical_technique	2,160	2,160	1,973	100.00 %	**91.34 % [90.08, 92.46]**
tnm_descriptor	2,160	2,160	2,157	100.00 %	**99.86 % [99.59, 99.95]**
tumor_budding	2,160	2,160	2,160	100.00 %	**100.00 % [99.82, 100.00]**
tumor_invasion	2,160	2,160	1,906	100.00 %	**88.24 % [86.81, 89.53]**
type_of_polyp	2,160	2,160	2,139	100.00 %	**99.03 % [98.52, 99.36]**

Supplementary Table S17. Per-field extraction accuracy for esophageal cancer excision reports (30-run aggregate, 16 schema fields).

Per-field extraction accuracy of the per-organ extractor on the esophagus subset of the CMUH validation cohort, **30 multi-seed runs of gpt-oss-20b** against the committee-resolved gold standard. n = 2,169 case-runs per field (73 unique reports × ~30 effective runs). Schema definition: Supplementary Table S4.

Field	n_total	n_attempted	n_correct	Coverage	Effective accuracy [95 % Wilson CI]
cancer_primary_site	2,169	2,169	2,009	100.00 %	**92.62 % [91.45, 93.65]**
distant_metastasis	2,169	2,169	315	100.00 %	**14.52 % [13.10, 16.07]**
extranodal_extension	2,169	2,169	2,132	100.00 %	**98.29 % [97.66, 98.76]**
grade	2,169	2,169	2,169	100.00 %	**100.00 % [99.82, 100.00]**
histology	2,169	2,169	2,169	100.00 %	**100.00 % [99.82, 100.00]**
lymphovascular_invasion	2,169	2,169	2,169	100.00 %	**100.00 % [99.82, 100.00]**
maximal_ln_size	2,169	2,169	2,169	100.00 %	**100.00 % [99.82, 100.00]**
perineural_invasion	2,169	2,169	2,169	100.00 %	**100.00 % [99.82, 100.00]**
pm_category	2,169	2,169	2,074	100.00 %	**95.62 % [94.68, 96.40]**
pn_category	2,169	2,169	2,169	100.00 %	**100.00 % [99.82, 100.00]**
procedure	2,169	2,169	2,129	100.00 %	**98.16 % [97.50, 98.64]**
pt_category	2,169	2,169	2,166	100.00 %	**99.86 % [99.59, 99.95]**
stage_group	2,169	2,169	2,082	100.00 %	**95.99 % [95.08, 96.74]**
surgical_technique	2,169	2,169	2,134	100.00 %	**98.39 % [97.76, 98.84]**
tnm_descriptor	2,169	2,169	2,122	100.00 %	**97.83 % [97.13, 98.37]**
tumor_extent	2,169	2,169	2,125	100.00 %	**97.97 % [97.29, 98.49]**

Supplementary Table S18. Per-field extraction accuracy for hepatocellular carcinoma excision reports (30-run aggregate, 17 schema fields).

Per-field extraction accuracy of the per-organ extractor on the liver subset of the CMUH validation cohort, **30 multi-seed runs of gpt-oss-20b** against the committee-resolved gold standard. n = 2,400 case-runs per field (80 unique reports × 30 effective runs). Schema definition: Supplementary Table S6.

Field	n_total	n_attempted	n_correct	Coverage	Effective accuracy [95 % Wilson CI]
distant_metastasis	2,400	2,400	84	100.00 %	**3.50 % [2.84, 4.31]**
extranodal_extension	2,400	2,400	2,399	100.00 %	**99.96 % [99.76, 99.99]**
grade	2,400	2,400	2,400	100.00 %	**100.00 % [99.84, 100.00]**
histology	2,400	2,400	2,367	100.00 %	**98.63 % [98.08, 99.02]**
maximal_ln_size	2,400	2,400	2,400	100.00 %	**100.00 % [99.84, 100.00]**
overall_stage	2,400	2,400	2,336	100.00 %	**97.33 % [96.61, 97.91]**
perineural_invasion	2,400	2,400	2,370	100.00 %	**98.75 % [98.22, 99.12]**
pm_category	2,400	2,400	2,274	100.00 %	**94.75 % [93.78, 95.57]**
pn_category	2,400	2,400	1,839	100.00 %	**76.62 % [74.89, 78.27]**
procedure	2,400	2,400	2,041	100.00 %	**85.04 % [83.56, 86.41]**
pt_category	2,400	2,400	2,382	100.00 %	**99.25 % [98.82, 99.53]**
tnm_descriptor	2,400	2,400	2,042	100.00 %	**85.08 % [83.60, 86.45]**
tumor_extent	2,400	2,400	2,370	100.00 %	**98.75 % [98.22, 99.12]**
tumor_focality	2,400	2,400	2,400	100.00 %	**100.00 % [99.84, 100.00]**
tumor_site	2,400	2,400	2,248	100.00 %	**93.67 % [92.62, 94.57]**
tumor_size	2,400	2,400	2,236	100.00 %	**93.17 % [92.09, 94.11]**
vascular_invasion	2,400	2,400	2,332	100.00 %	**97.17 % [96.42, 97.76]**

Supplementary Table S19. Per-field extraction accuracy for lung cancer excision reports (30-run aggregate, 20 schema fields).

Per-field extraction accuracy of the per-organ extractor on the lung subset of the CMUH validation cohort, **30 multi-seed runs of gpt-oss-20b** against the committee-resolved gold standard. n = 1,650 case-runs per field (55 unique reports × 30 effective runs). Schema definition: Supplementary Table S2.

Field	n_total	n_attempted	n_correct	Coverage	Effective accuracy [95 % Wilson CI]
cancer_primary_site	1,650	1,650	1,649	100.00 %	**99.94 % [99.66, 99.99]**
direct_invasion_of_adjacent_structures	1,650	1,650	1,518	100.00 %	**92.00 % [90.59, 93.21]**
distant_metastasis	1,650	1,650	215	100.00 %	**13.03 % [11.49, 14.74]**
extranodal_extension	1,650	1,556	1,533	94.30 %	**92.91 % [91.57, 94.05]** (att 98.52 %)
grade	1,650	1,650	1,628	100.00 %	**98.67 % [97.99, 99.12]**
histology	1,650	1,650	1,638	100.00 %	**99.27 % [98.73, 99.58]**
lymphovascular_invasion	1,650	1,650	1,618	100.00 %	**98.06 % [97.28, 98.62]**
maximal_ln_size	1,650	1,556	1,537	94.30 %	**93.15 % [91.83, 94.27]** (att 98.78 %)
perineural_invasion	1,650	1,650	1,380	100.00 %	**83.64 % [81.77, 85.34]**
pm_category	1,650	1,647	1,491	99.82 %	**90.36 % [88.84, 91.70]**

pn_category	1,650	1,647	1,593	99.82 %	**96.55 % [95.55, 97.32]**
procedure	1,650	1,650	1,615	100.00 %	**97.88 % [97.06, 98.47]**
pt_category	1,650	1,647	1,592	99.82 %	**96.48 % [95.48, 97.27]**
sideness	1,650	1,650	1,650	100.00 %	**100.00 % [99.77, 100.00]**
spread_through_air_spaces_stas	1,650	1,650	1,585	100.00 %	**96.06 % [95.01, 96.90]**
stage_group	1,650	1,647	1,533	99.82 %	**92.91 % [91.57, 94.05]**
surgical_technique	1,650	1,650	1,459	100.00 %	**88.42 % [86.79, 89.88]**
tnm_descriptor	1,650	1,647	1,454	99.82 %	**88.12 % [86.47, 89.59]**
tumor_focality	1,650	1,650	1,547	100.00 %	**93.76 % [92.49, 94.83]**
visceral_pleural_invasion	1,650	1,650	1,611	100.00 %	**97.64 % [96.79, 98.27]**

Supplementary Table S20. Per-field extraction accuracy for pancreas cancer excision reports (30-run aggregate, 15 schema fields).

Per-field extraction accuracy of the per-organ extractor on the pancreas subset of the CMUH validation cohort, **30 multi-seed runs of gpt-oss-20b** against the committee-resolved gold standard. n = 750 case-runs per field (25 unique reports × 30 effective runs). Schema definition: Supplementary Table S7.

Field	n_total	n_attempted	n_correct	Coverage	Effective accuracy [95 % Wilson CI]
distant_metastasis	750	750	89	100.00 %	**11.87 % [9.74, 14.38]**
extranodal_extension	750	724	706	96.53 %	**94.13 % [92.22, 95.60]** (att 97.51 %)
histology	750	750	727	100.00 %	**96.93 % [95.44, 97.95]**
lymphovascular_invasion	750	750	750	100.00 %	**100.00 % [99.49, 100.00]**
maximal_ln_size	750	724	723	96.53 %	**96.40 % [94.81, 97.51]** (att 99.86 %)
overall_stage	750	750	750	100.00 %	**100.00 % [99.49, 100.00]**
perineural_invasion	750	750	750	100.00 %	**100.00 % [99.49, 100.00]**
pm_category	750	750	722	100.00 %	**96.27 % [94.66, 97.40]**
pn_category	750	750	750	100.00 %	**100.00 % [99.49, 100.00]**
procedure	750	750	679	100.00 %	**90.53 % [88.23, 92.43]**
pt_category	750	750	750	100.00 %	**100.00 % [99.49, 100.00]**
tnm_descriptor	750	750	611	100.00 %	**81.47 % [78.53, 84.08]**
tumor_extension	750	750	578	100.00 %	**77.07 % [73.92, 79.93]**
tumor_site	750	750	720	100.00 %	**96.00 % [94.35, 97.18]**
tumor_size	750	750	662	100.00 %	**88.27 % [85.77, 90.38]**

Supplementary Table S21. Per-field extraction accuracy for prostate cancer excision reports (30-run aggregate, 28 schema fields).

Per-field extraction accuracy of the per-organ extractor on the prostate subset of the CMUH validation cohort, **30 multi-seed runs of gpt-oss-20b** against the committee-resolved gold standard. n = 2,820 case-runs per field (94 unique reports × 30 effective runs). Schema definition: Supplementary Table S8.

Field	n_total	n_attempted	n_correct	Coverage	Effective accuracy [95 % Wilson CI]
bladder_invasion	2,820	2,820	2,802	100.00 %	**99.36 % [98.99, 99.60]**
cribriform_pattern_presence	2,820	2,820	2,694	100.00 %	**95.53 % [94.71, 96.23]**
distant_metastasis	2,820	2,820	77	100.00 %	**2.73 % [2.19, 3.40]**
extranodal_extension	2,820	2,818	2,753	99.93 %	**97.62 % [96.99, 98.12]**
extraprostatic_extension	2,820	2,820	2,820	100.00 %	**100.00 % [99.86, 100.00]**
gleason_4_percentage	2,820	2,820	2,737	100.00 %	**97.06 % [96.37, 97.62]**
gleason_5_percentage	2,820	2,820	2,727	100.00 %	**96.70 % [95.98, 97.30]**
grade	2,820	2,820	2,807	100.00 %	**99.54 % [99.21, 99.73]**
histology	2,820	2,820	2,815	100.00 %	**99.82 % [99.59, 99.92]**
intraductal_carcinoma_presence	2,820	2,820	2,807	100.00 %	**99.54 % [99.21, 99.73]**
involved_margin_list	2,820	2,809	2,616	99.61 %	**92.77 % [91.75, 93.67]** (att 93.13 %)
lymphovascular_invasion	2,820	2,820	2,820	100.00 %	**100.00 % [99.86, 100.00]**
margin_length	2,820	2,809	2,809	99.61 %	**99.61 % [99.30, 99.78]** (att 100.00 %)
margin_positivity	2,820	2,809	2,808	99.61 %	**99.57 % [99.26, 99.76]** (att 99.96 %)
maximal_ln_size	2,820	2,818	2,817	99.93 %	**99.89 % [99.69, 99.96]**

perineural_invasion	2,820	2,820	2,820	100.00 %	**100.00 % [99.86, 100.00]**
pm_category	2,820	2,820	2,570	100.00 %	**91.13 % [90.03, 92.13]**
pn_category	2,820	2,820	2,819	100.00 %	**99.96 % [99.80, 99.99]**
procedure	2,820	2,820	2,820	100.00 %	**100.00 % [99.86, 100.00]**
prostate_size	2,820	2,820	2,762	100.00 %	**97.94 % [97.35, 98.41]**
prostate_weight	2,820	2,820	2,816	100.00 %	**99.86 % [99.64, 99.94]**
pt_category	2,820	2,820	2,820	100.00 %	**100.00 % [99.86, 100.00]**
seminal_vesicle_invasion	2,820	2,820	2,820	100.00 %	**100.00 % [99.86, 100.00]**
stage_group	2,820	2,820	2,657	100.00 %	**94.22 % [93.30, 95.02]**
surgical_technique	2,820	2,820	2,813	100.00 %	**99.75 % [99.49, 99.88]**
tnm_descriptor	2,820	2,820	2,205	100.00 %	**78.19 % [76.63, 79.68]**
tumor_percentage	2,820	2,820	2,749	100.00 %	**97.48 % [96.84, 97.99]**
tumor_size	2,820	2,820	2,818	100.00 %	**99.93 % [99.74, 99.98]**

Supplementary Table S22. Per-field extraction accuracy for gastric cancer excision reports (30-run aggregate, 18 schema fields).

Per-field extraction accuracy of the per-organ extractor on the gastric subset of the CMUH validation cohort, **30 multi-seed runs of gpt-oss-20b** against the committee-resolved gold standard. n = 2,340 case-runs per field (78 unique reports × 30 effective runs). Schema definition: Supplementary Table S5.

Field	n_total	n_attempted	n_correct	Coverage	Effective accuracy [95 % Wilson CI]
cancer_primary_site	2,340	2,340	1,978	100.00 %	**84.53 % [83.01, 85.94]**
distant_metastasis	2,340	2,340	292	100.00 %	**12.48 % [11.20, 13.88]**
extracellular_mucin	2,340	2,340	2,340	100.00 %	**100.00 % [99.84, 100.00]**
extranodal_extension	2,340	2,265	2,009	96.79 %	**85.85 % [84.38, 87.21]** (att 88.70 %)
grade	2,340	2,340	2,340	100.00 %	**100.00 % [99.84, 100.00]**
histology	2,340	2,340	2,261	100.00 %	**96.62 % [95.81, 97.28]**
lymphovascular_invasion	2,340	2,340	2,340	100.00 %	**100.00 % [99.84, 100.00]**
maximal_ln_size	2,340	2,265	2,264	96.79 %	**96.75 % [95.95, 97.40]** (att 99.96 %)
perineural_invasion	2,340	2,340	2,340	100.00 %	**100.00 % [99.84, 100.00]**
pm_category	2,340	2,340	2,152	100.00 %	**91.97 % [90.79, 93.00]**
pn_category	2,340	2,340	2,338	100.00 %	**99.91 % [99.69, 99.98]**
procedure	2,340	2,340	2,276	100.00 %	**97.26 % [96.52, 97.85]**
pt_category	2,340	2,340	2,340	100.00 %	**100.00 % [99.84, 100.00]**
signet_ring	2,340	2,340	2,340	100.00 %	**100.00 % [99.84, 100.00]**
stage_group	2,340	2,340	2,305	100.00 %	**98.50 % [97.93, 98.92]**
surgical_technique	2,340	2,340	1,135	100.00 %	**48.50 % [46.48, 50.53]**

tnm_descriptor	2,340	2,340	1,925	100.00 %	**82.26 % [80.66, 83.76]**
tumor_extent	2,340	2,340	2,282	100.00 %	**97.52 % [96.81, 98.08]**

Supplementary Table S23. Per-field extraction accuracy for thyroid cancer excision reports (30-run aggregate, 18 schema fields).

Per-field extraction accuracy of the per-organ extractor on the thyroid subset of the CMUH validation cohort, **30 multi-seed runs of gpt-oss-20b** against the committee-resolved gold standard. n = 2,160 case-runs per field (72 unique reports × 30 effective runs). Schema definition: Supplementary Table S10.

Field	n_total	n_attempted	n_correct	Coverage	Effective accuracy [95 % Wilson CI]
distant_metastasis	2,160	2,160	1,524	100.00 %	**70.56 % [68.60, 72.44]**
extranodal_extension	2,160	2,160	1,980	100.00 %	**91.67 % [90.43, 92.76]**
extrathyroid_extension	2,160	2,160	2,114	100.00 %	**97.87 % [97.17, 98.40]**
histology	2,160	2,160	2,131	100.00 %	**98.66 % [98.08, 99.06]**
lymphovascular_invasion	2,160	2,160	1,631	100.00 %	**75.51 % [73.65, 77.28]**
maximal_ln_size	2,160	2,160	2,128	100.00 %	**98.52 % [97.92, 98.95]**
mitotic_activity	2,160	2,160	2,129	100.00 %	**98.56 % [97.97, 98.99]**
overall_stage	2,160	2,160	2,079	100.00 %	**96.25 % [95.36, 96.97]**
perineural_invasion	2,160	2,160	1,602	100.00 %	**74.17 % [72.28, 75.97]**
pm_category	2,160	2,160	2,022	100.00 %	**93.61 % [92.50, 94.57]**
pn_category	2,160	2,160	2,006	100.00 %	**92.87 % [91.71, 93.88]**
predisposing_condition	2,160	2,160	2,153	100.00 %	**99.68 % [99.33, 99.84]**
procedure	2,160	2,160	1,916	100.00 %	**88.70 % [87.30, 89.97]**
pt_category	2,160	2,160	2,159	100.00 %	**99.95 % [99.74, 99.99]**
tnm_descriptor	2,160	2,160	1,970	100.00 %	**91.20 % [89.93, 92.33]**
tumor_focality	2,160	2,160	2,069	100.00 %	**95.79 % [94.86, 96.56]**
tumor_necrosis	2,160	2,160	1,839	100.00 %	**85.14 % [83.58, 86.58]**

tumor_site	2,160	2,160	2,044	100.00 %	**94.63 % [93.60, 95.50]**
tumor_size	2,160	2,160	2,122	100.00 %	**98.24 % [97.59, 98.72]**

Supplementary Table S24. Detailed numeric results for lymph-node extraction accuracy by organ.

Aggregate lymph-node extraction concordance across the original CMUH validation cohort (n = 893 reports). Carried over from the v1 manuscript supplementary (single-run snapshot). The headline 30-run aggregates that supersede this in the revised text are at Supplementary Table S34 (per-organ LN summary, k = 30) and Figure 4. Two metrics + four descriptive rows; the prevalence-vs-correctness split is not separated in the v1 design (the v2 design in Supplementary Table S34 separates examined-count, examined-tol-1, group-recall, and group-precision into four explicit metrics).

Metric	breast	cervix	colorectal	esophagus	liver	lung	pancreas	prostate	stomach	thyroid
lymph node number concordance rate	93.30 %	86.20 %	97.10 %	49.30 %	98.70 %	82.50 %	92.80 %	59.60 %	83.50 %	91.80 %
lymph node station concordance rate	80.00 %	71.40 %	96.90 %	49.30 %	100.00 %	77.70 %	85.50 %	58.20 %	91.00 %	93.00 %
lymph node stations (total annotated)	95	77	127	454	12	215	62	275	401	43
report numbers	75	29	70	73	79	63	28	94	79	74
station per report	1.27	2.66	1.81	6.22	0.15	3.41	2.21	2.93	5.08	0.58

Supplementary Table S25. Eligibility-classifier 30-run aggregate.

Aggregate reliability statistics for the eligibility classifier (Stage A) across the 30 multi-seed runs of gpt-oss-20b on the CMUH validation cohort (n = 893 reports → 26,790 case-runs). Reports the headline reliability triplet (single-run ICC, average-rater ICC, Cronbach's α) plus accuracy flip rate and per-case SD summary. This is the headline reliability number cited in Section 3.2 (Table 4), Section 3.8, and the overall multi-run reliability discussion. Replaces the single-run / 3-run confusion matrices at Supplementary Tables S11–S13 as the headline number for Stage A.

Statistic	Value
Chapter	1 (`chapter1_eligibility`)
Stage	A
n_cases	893
n_runs	30
n_case-runs	26,790
ICC(2,1) — single-run reproducibility	**0.8893** [0.8798, 0.8985]
ICC(3, k = 30) — average-of-30-runs	**0.9959** [0.9955, 0.9963]
Cronbach's α	0.9959
Accuracy flip rate (per-case across 30 runs)	**3.58 %**
Per-case SD mean (binary accuracy)	0.0123
Per-case SD 90th percentile	0.0000

Supplementary Table S26. Organ-classification 30-run aggregate.

Aggregate reliability statistics for the 11-class organ-labeling sub-model (Stage B) across the 30 multi-seed runs of gpt-oss-20b on the CMUH validation cohort, scored only on the 682 reports that cleared the eligibility classifier (n = 682 reports → 20,460 case-runs evaluated; 71 case-runs filtered as triage failures). This is the headline reliability number cited in Section 3.2 and the replacement for the 3-run confusion matrices at Supplementary Tables S11–S13.

Statistic	Value
Chapter	2 (`chapter2_organ_classification`)
Stage	B
n_cases	682
n_runs	30
n_case-runs	20,460 (after Stage-A filter)
ICC(2,1) — single-run reproducibility	**0.8996** [0.8896, 0.9092]
ICC(3, k = 30) — average-of-30-runs	**0.9963** [0.9959, 0.9967]
Cronbach's α	0.9963
Accuracy flip rate (per-case across 30 runs)	**1.76 %**
Per-case SD mean (binary accuracy)	0.0069
Per-case SD 90th percentile	0.0000

Supplementary Table S27. Per-organ extraction-accuracy headline (k = 30, paper scope).

Per-organ effective extraction accuracy on the CMUH validation cohort, aggregated across 30 multi-seed runs of gpt-oss-20b. The "paper scope" denominator excludes biomarker sub-fields scored separately in Section 3.5 (breast ER/PR/HER2/Ki-67) and the prostate-specific margin scalars scored separately in Section 3.4 (Supplementary Table S33). This is the headline per-organ table cited in Section 3.3 (Table 5) and Figure 3; sorted descending by effective accuracy.

Organ	n_fields	n_attempted (case × run × field)	n_correct	Effective accuracy [95 % Wilson CI]
prostate	25	70,496	66,165	**93.86 % [93.68, 94.03]**
colorectal	20	43,200	40,428	**93.58 % [93.35, 93.81]**
esophagus	16	34,704	32,302	**93.08 % [92.81, 93.34]**
thyroid	19	41,040	37,618	**91.66 % [91.39, 91.93]**
breast	26	58,442	53,482	**91.51 % [91.28, 91.74]**
lung	20	32,797	29,846	**91.00 % [90.69, 91.31]**
liver	17	40,800	36,520	**89.51 % [89.21, 89.80]**
pancreas	15	11,198	9,967	**89.01 % [88.41, 89.57]**
stomach	18	41,970	37,257	**88.77 % [88.46, 89.07]**
cervix	16	12,940	11,247	**86.92 % [86.32, 87.49]**
**Macro mean**				**90.89 %**

Supplementary Table S28. Anatomic vs pathologic stage-group head-to-head (paired breast case-runs).

Per-field exact-match accuracy for `anatomic_stage_group` and `pathologic_stage_group` on the breast subset of the CMUH validation cohort, paired by case × run across the 30-seed pass (n = 2,248 paired comparisons; 75 cases × ~30 effective runs after Stage A/B gating). The two fields share the same case-run denominator (the same set of breast case-runs is asked to populate both fields), so the 23.09 percentage-point gap between them is a within-case effect — not a sampling difference. This is the headline number for Section 3.3.1 (Figure 4) and the qualitative case study in Appendix A4.

Field	n_total	n_correct	Effective accuracy [95 % Wilson CI]
anatomic_stage_group	2,248	2,202	**97.95 % [97.28, 98.46]**
pathologic_stage_group	2,248	1,683	**74.87 % [73.03, 76.62]**
**Δ (anatomic − pathologic)**	—	—	**+23.09 pp** (95 % Wilson intervals do not overlap)

Supplementary Table S29. Bottom-decile field error attribution.

Bottom-decile fields by effective accuracy across the CMUH 30-run pass, with each field tagged by mechanism (M1–M6, or audit-pending). Eleven fields, sorted ascending by effective accuracy; the lowest is `distant_metastasis` at 19.06 %. Backs Section 3.3.2 (Figure 7). The six mechanisms separate the proximate cause of the low score by its actual engineering fix path so that the discussion in paragraph 10 can address each independently.

Field	Mechanism	n_total	n_correct	Effective accuracy [95 % Wilson CI]	Coverage	Cohen's κ vs gold
distant_metastasis	M4_convention	19,507	3,718	**19.06 % [18.51, 19.62]**	1.000	0.947
cancer_quadrant	M2_glossary_rule	2,248	1,629	**72.46 % [70.58, 74.27]**	1.000	0.745
pathologic_stage_group	M5_schema_shape	2,248	1,683	**74.87 % [73.03, 76.62]**	1.000	0.927
tumor_extension	— (audit pending)	750	578	**77.07 % [73.92, 79.93]**	1.000	0.609
biomarker_her2	M5_schema_shape	2,248	1,738	**77.31 % [75.54, 79.00]**	1.000	—
surgical_technique	M2_glossary_rule	11,949	10,084	**84.39 % [83.73, 85.03]**	1.000	0.955
tumor_necrosis	M2_glossary_rule	2,160	1,839	**85.14 % [83.58, 86.58]**	1.000	—
cancer_clock	M2_glossary_rule	2,248	1,918	**85.32 % [83.80, 86.72]**	1.000	0.949
biomarker_ki67	M5_schema_shape	2,248	1,939	**86.25 % [84.77, 87.62]**	1.000	—
tumor_invasion	M6_open_conflation	2,160	1,906	**88.24 % [86.81, 89.53]**	1.000	0.837
tnm_descriptor	M5_schema_shape	19,507	17,337	**88.88 % [88.43, 89.31]**	1.000	0.954

Supplementary Table S30. Multi-primary Stage-B disposition breakdown.

The multi-primary triage decomposition for the gold-`others` ledger across the 30-run pass on the CMUH validation cohort. Stage-B disposition rows (`both_others` = both annotators agreed the case is out of scope; `gold_only` = gold says `others`, model emitted an in-scope organ; `pred_only` = model emitted `others`, gold is in scope) are decomposed by clinical flavour (out-of-scope site, synchronous multi-primary, uncertain). This is the headline number for Section Multi-Primary and Out-of-Scope TriageFigure 2).

Stage-B disposition	out_of_scope_site	synchronous_multi_primary	uncertain	row total
both_others	437	0	12	449
gold_only	163	180	288	631
pred_only	22	0	36	58
**column total**	**622**	**180**	**336**	**1,138**

Supplementary Table S31. Per-organ misroute destinations for gold-`others` reports.

When the gold annotator codes a report as `others` and the model emits an in-scope organ instead, this table records which in-scope organ the model picks. Counts are case × run cells (n = 631 total across the 30-run pass — the `gold_only` row of Supplementary Table S30). Sorted descending by count.

Gold cancer_category	Predicted cancer_category	n
others	lung	150
others	(null / no prediction)	117
others	pancreas	108
others	stomach	60
others	colorectal	60
others	cervix	50
others	breast	30
others	liver	30
others	esophagus	26
**total**	—	**631**

Supplementary Table S32. Per-organ margin extraction summary (positivity, distance accuracy, hallucination, miss).

Per-organ margin-extraction metrics on the CMUH 30-run pass: list-level F1 plus the hallucination-vs-miss decomposition of the residual error. Nine organs (prostate excluded — its margin design uses a separate binary `margin_positivity` + `involved_margin_list` schema, see Supplementary Tables S8 and S33). Backs Section 3.4 (Supplementary Figures S13 + S12,). The hallucination_rate column is the dominant residual error: in seven of nine organs hallucination is at least 5× the miss rate, with lung exhibiting the most extreme schema-driven over-prediction (49.8 % hallucination vs 2.8 % miss).

Organ	n_total	n_attempted	Coverage	Any-involved correctness [95 % CI]	Closest-dist MAE (mm)	Closest-dist accuracy (±2 mm tol)	Micro F1	Hallucination rate [95 % CI]	Miss rate [95 % CI]
breast	2,248	2,248	100.0 %	**93.91 % [92.84, 94.82]**	0.21	97.98 %	0.9506	9.30 % [8.84, 9.78]	0.14 % [0.09, 0.22]
cervix	810	810	100.0 %	**99.51 % [98.74, 99.81]**	0.81	93.86 %	0.8905	13.50 % [12.18, 14.94]	8.24 % [7.17, 9.46]
colorectal	2,160	2,082	96.4 %	**98.46 % [97.84, 98.91]**	7.15	73.54 %	0.9573	5.79 % [5.32, 6.30]	2.70 % [2.37, 3.07]
esophagus	2,169	2,168	99.95 %	**96.08 % [95.18, 96.82]**	4.90	78.40 %	0.8373	26.89 % [25.99, 27.81]	2.03 % [1.72, 2.39]
liver	2,400	2,400	100.0 %	**98.71 % [98.17, 99.09]**	0.21	97.11 %	0.7832	33.52 % [32.22, 34.84]	4.71 % [4.06, 5.47]
lung	1,650	1,647	99.8 %	**98.42 % [97.70, 98.92]**	9.25	46.45 %	0.6617	**49.83 % [48.54, 51.13]**	2.83 % [2.29, 3.50]
pancreas	750	737	98.3 %	**93.62 % [91.62, 95.17]**	4.38	85.01 %	0.9189	11.43 % [10.50, 12.44]	4.52 % [3.90, 5.22]
stomach	2,340	2,340	100.0 %	**98.25 % [97.63, 98.71]**	5.67	80.50 %	0.8485	25.18 % [24.19, 26.20]	2.02 % [1.68, 2.42]
thyroid	2,160	2,160	100.0 %	**99.58 % [99.21, 99.78]**	0.52	97.57 %	0.7613	37.88 % [36.70, 39.08]	1.69 % [1.34, 2.14]
(prostate)	2,820	0	0.0 %	—	—	—	—	—	—

Supplementary Table S33. Prostate-specific margin extraction callout.

The prostate schema (Supplementary Table S8) uses a binary `margin_positivity` flag plus a list-typed `involved_margin_list` (12 named directions) instead of the per-margin list-of-records design used by the other nine organs. Three prostate-specific margin scalars are scored independently of the Section 3.4 margin metrics in Supplementary Table S32; this table records their accuracy on the 30-run pass. Backs Section 3.4.

Field	n_total	n_correct	Effective accuracy [95 % Wilson CI]
margin_positivity	2,820	2,808	**99.57 % [99.26, 99.76]**
margin_length	2,820	2,809	**99.61 % [99.30, 99.78]**
involved_margin_list	2,820	2,616	**92.77 % [91.75, 93.67]**

Supplementary Table S34. Per-organ lymph-node extraction summary (30-run, four-metric decomposition).

Per-organ lymph-node-extraction metrics on the CMUH 30-run pass, decomposed into four explicit measurements (examined-count tolerance-1 accuracy, any-positive correctness, group recall, group precision). Replaces the two-metric v1 design in Supplementary Table S24 with separate column for each measurement. Backs Section 3.4). Sorted by `group_recall` descending; esophagus, cervix, and prostate are flagged as the low-recall tier and have station-name canonicalization callouts in Figure 4.

Organ	n_total	n_attempted	Coverage	Examined-count MAE	Examined accuracy (±1 tol)	Any-positive correctness [95 % CI]	Group recall	Group precision	Hallucination rate	Miss rate
liver	2,400	2,400	100.0 %	0.04	98.75 %	**100.0 % [99.8, 100.0]**	**0.980**	0.993	0.68 %	2.00 %
colorectal	2,160	2,160	100.0 %	0.33	95.97 %	**99.8 % [99.6, 99.9]**	**0.973**	0.974	3.33 %	3.16 %
stomach	2,340	2,265	96.8 %	2.64	81.55 %	**99.7 % [99.4, 99.9]**	**0.837**	0.945	4.33 %	14.62 %
breast	2,248	2,245	99.9 %	0.42	91.31 %	**99.9 % [99.6, 100.0]**	**0.844**	0.936	6.00 %	15.76 %
thyroid	2,160	2,160	100.0 %	0.69	89.95 %	**98.3 % [97.7, 98.8]**	**0.717**	0.793	21.83 %	28.81 %
pancreas	750	724	96.5 %	0.64	90.33 %	**100.0 % [99.5, 100.0]**	**0.776**	0.835	15.22 %	22.49 %
lung	1,650	1,556	94.3 %	1.15	89.72 %	**100.0 % [99.8, 100.0]**	**0.783**	0.866	13.94 %	22.74 %
prostate	2,820	2,818	99.9 %	7.02	61.57 %	**100.0 % [99.9, 100.0]**	**0.629**	0.945	5.87 %	37.05 %
cervix	810	800	98.8 %	3.14	67.00 %	**100.0 % [99.5, 100.0]**	**0.493**	0.542	36.07 %	49.32 %
esophagus	2,169	2,169	100.0 %	10.45	45.50 %	**100.0 % [99.8, 100.0]**	**0.426**	0.826	13.09 %	58.22 %

Supplementary Table S35. Multi-run reliability (k = 30) — full per-chapter, per-organ detail.

Multi-run reliability statistics across all four chapters of the evaluation pipeline (chapter 1 eligibility, chapter 2 organ classification, chapter 4 margins, chapter 5 lymph nodes) on the CMUH 30-run pass. Chapters 1 and 2 are scored under ICC (binary classification across runs); chapters 4 and 5 are scored under per-case

F1 standard deviation (list-typed extraction, where ICC is not well-defined). This is the headline reliability table cited in Section 2.4.2 (Methods, paragraph 02) and Section 3.8 (paragraph 16), with companion plots in Supplementary Figures S14 and S15. Twenty-one rows: 2 upstream classification stages + 9 organ-level margins + 10 organ-level lymph nodes.

Chapter	Stage	Organ	Field	n_cases	n_runs	ICC(2,1) [95 % CI]	ICC(3, k = 30) [95 % CI]	Cronbach α	Acc flip rate	Per-case SD mean	Per-case SD p90	Mean per-case F1	F1 SD mean	F1 SD p90	Missing flip rate
1	A	—	—	893	30	0.8893 [0.8798, 0.8985]	**0.9959 [0.9955, 0.9963]**	0.9959	3.58 %	0.0123	0.0000	—	—	—	—
2	B	—	—	682	30	0.8996 [0.8896, 0.9092]	**0.9963 [0.9959, 0.9967]**	0.9963	1.76 %	0.0069	0.0000	—	—	—	—
4	—	breast	margins	74	30	—	—	—	—	—	—	**0.9572**	0.0249	0.0454	0.0000
4	—	cervix	margins	27	30	—	—	—	—	—	—	**0.8945**	0.0947	0.1554	0.0000
4	—	colorectal	margins	72	30	—	—	—	—	—	—	**0.9422**	0.0458	0.0767	0.5833
4	—	esophagus	margins	73	30	—	—	—	—	—	—	**0.8501**	0.0889	0.1224	0.0141
4	—	liver	margins	80	30	—	—	—	—	—	—	**0.8224**	0.0911	0.2424	0.0000
4	—	lung	margins	55	30	—	—	—	—	—	—	**0.6852**	0.1621	0.2414	0.0545
4	—	pancreas	margins	25	30	—	—	—	—	—	—	**0.8814**	0.0769	0.1851	0.2000
4	—	stomach	margins	79	30	—	—	—	—	—	—	**0.8726**	0.1105	0.1711	0.0000
4	—	thyroid	margins	72	30	—	—	—	—	—	—	**0.7900**	0.1137	0.2122	0.0000
5	—	breast	regional_lymph_node	66	30	—	—	—	—	—	—	**0.8444**	0.2154	0.4552	0.0270
5	—	cervix	regional_lymph_node	21	30	—	—	—	—	—	—	**0.4888**	0.3620	0.4714	0.2593
5	—	colorectal	regional_lymph_node	67	30	—	—	—	—	—	—	**0.9659**	0.0588	0.1795	0.0000
5	—	esophagus	regional_lymph_node	73	30	—	—	—	—	—	—	**0.4444**	0.3537	0.4714	0.0000
5	—	liver	regional_lymph_node	11	30	—	—	—	—	—	—	**0.9800**	0.0552	0.1988	0.0000
5	—	lung	regional_lymph_node	45	30	—	—	—	—	—	—	**0.8067**	0.2760	0.4193	0.1091
5	—	pancreas	regional_lymph_node	24	30	—	—	—	—	—	—	**0.7785**	0.2299	0.4422	0.1200
5	—	prostate	regional_lymph_node	93	30	—	—	—	—	—	—	**0.6318**	0.4004	0.4955	0.0213
5	—	stomach	regional_lymph_node	77	30	—	—	—	—	—	—	**0.8575**	0.1919	0.3429	0.0933
5	—	thyroid	regional_lymph_node	47	30	—	—	—	—	—	—	**0.6991**	0.2750	0.4899	0.0000

Supplementary Table S36. TCGA multi-run cascade headline (Stages A, B, C).

Cascade-stage headline for the local-LLM (gpt-oss-20b) pipeline on the external TCGA-Reports cohort (n = 242 reports, six organs, k = 22 multi-run seeds). Stage A (eligibility triage), Stage B (organ classification), Stage C (per-organ field extraction macro mean) are reported separately because their denominators differ. Backs Section 3.9 / paragraph 17 (the headline numbers in the first paragraph of Section 3.9).

Stage	Stage name	n_total	n_correct	Accuracy [95 % CI]	Cohen's κ	Note
A	Eligibility (multi-run, run × case)	5,324	4,828	**90.68 % [89.87, 91.44]**	0.0001	TCGA gold is heavily positive-skewed (5,302 vs 22); κ near 0 reflects class imbalance, not classifier failure.
B	Organ classification (multi-run, run × case)	4,828	4,770	**98.80 % [98.45, 99.07]**	0.9850	macro-F1 = 0.850.
C	Field extraction (per-organ macro mean)	96,461	77,437	**79.92 %**	—	Unique cases: 242 cases at A → 241 → 239 at C; 22 multi-run seeds aggregated.

Supplementary Table S37. TCGA Stage-C field extraction — per-organ accuracy.

Per-organ Stage-C effective accuracy on the external TCGA-Reports cohort under the local-LLM (gpt-oss-20b) pipeline (k = 22 multi-run seeds, 242 reports across six organs). Counts are case × run × field cells. Sorted descending by effective accuracy. Backs Section 3.9 and Supplementary Figure S6.

Organ	n_attempted	n_correct	Effective accuracy [95 % Wilson CI]
colorectal	20,144	17,466	**86.71 % [86.23, 87.17]**
esophagus	15,070	12,649	**83.93 % [83.34, 84.51]**
liver	16,660	13,090	**78.57 % [77.94, 79.19]**
stomach	15,114	11,664	**77.17 % [76.50, 77.84]**
thyroid	380	291	**76.58 % [72.07, 80.56]**
breast	29,093	22,277	**76.57 % [76.08, 77.05]**
**Macro mean**			**79.92 %**

Supplementary Table S38. TCGA Stage-C field extraction — per-field accuracy with structural-caveat explanations.

Per-field effective accuracy of the local-LLM (gpt-oss-20b) pipeline on the external TCGA-Reports cohort across the 22-seed multi-run pass (51 fields total). Sorted ascending by effective accuracy. Backs Section 3.9 and Supplementary Figure S7. The 13 lowest-accuracy fields carry caveat explanations that decompose the Section 3.9 internal-vs-external accuracy gap into two failure modes (Mode 1 = LLM hallucination from silent source / null gold; Mode 2 = coding-convention mismatch).

Field	n_total	n_attempted	n_correct	Coverage	Effective accuracy [95 % Wilson CI]	Caveat explanation
biomarker_pms2	1,006	6	0	0.60 %	**0.00 % [0.00, 0.38]**	MMR IHC not performed at TCGA institutions; gold null; LLM hallucinates when it emits (PMS2)
biomarker_msh6	1,006	6	0	0.60 %	**0.00 % [0.00, 0.38]**	MMR IHC not performed at TCGA institutions; gold null; LLM hallucinates when it emits (MSH6)
biomarker_msh2	1,006	6	0	0.60 %	**0.00 % [0.00, 0.38]**	MMR IHC not performed at TCGA institutions; gold null; LLM hallucinates when it emits (MSH2)
biomarker_mlh1	1,006	6	0	0.60 %	**0.00 % [0.00, 0.38]**	MMR IHC not performed at TCGA institutions; gold null; LLM hallucinates

						when it emits (MLH1)
biomarker_ki67	982	812	28	82.69 %	**2.85 % [1.98, 4.09]**	TCGA breast reports do not document Ki-67; LLM emits a default value
biomarker_her2	982	921	136	93.79 %	**13.85 % [11.83, 16.15]**	older TCGA breast reports omit HER2 testing; LLM emits a default value
overall_stage	1,000	1,000	145	100.00 %	**14.50 % [12.45, 16.82]**	AJCC edition usually unstated in TCGA; gold null; LLM emits a stage group regardless
stage_group	2,788	2,788	585	100.00 %	**20.98 % [19.51, 22.53]**	AJCC edition usually unstated in TCGA; gold null; LLM emits a stage group regardless
extrathyroid_extension	20	20	8	100.00 %	**40.00 % [21.88, 61.34]**	(n = 20; small denominator)
biomarker_er	982	917	436	93.38 %	**44.40 % [41.32, 47.52]**	older TCGA breast reports omit ER; LLM emits a default value
biomarker_pr	982	917	436	93.38 %	**44.40 % [41.32, 47.52]**	older TCGA breast reports omit PR; LLM emits a default value
pathologic_stage_group	982	981	517	99.90 %	**52.65 % [49.52, 55.75]**	AJCC edition usually unstated in TCGA; gold null; LLM

						emits a stage group regardless
cancer_quadrant	982	982	558	100.00 %	**56.82 % [53.70, 59.89]**	(no caveat)
anatomic_stage_group	982	981	564	99.90 %	**57.43 % [54.32, 60.49]**	AJCC edition usually unstated in TCGA; gold null; LLM emits a stage group regardless
pm_category	4,770	4,769	2,969	99.98 %	**62.24 % [60.86, 63.61]**	default-Mx rule asymmetry (LLMs sometimes emit pM0)
surgical_technique	2,788	2,788	1,853	100.00 %	**66.46 % [64.69, 68.19]**	(no caveat)
tumor_invasion	1,006	1,006	683	100.00 %	**67.89 % [64.94, 70.71]**	(no caveat)
dcis_present	982	982	733	100.00 %	**74.64 % [71.83, 77.26]**	(no caveat)
tnm_descriptor	4,770	4,769	3,615	99.98 %	**75.79 % [74.55, 76.98]**	(no caveat)
pn_category	4,770	4,769	3,716	99.98 %	**77.90 % [76.70, 79.06]**	(no caveat)
pt_category	4,770	4,769	3,876	99.98 %	**81.26 % [80.13, 82.34]**	(no caveat)
nuclear_grade	982	982	820	100.00 %	**83.50 % [81.05, 85.69]**	(no caveat)
dcis_size	982	982	828	100.00 %	**84.32 % [81.91, 86.46]**	(no caveat)
procedure	4,770	4,770	4,022	100.00 %	**84.32 % [83.26, 85.32]**	(no caveat)
mitotic_rate	982	982	830	100.00 %	**84.52 % [82.13, 86.65]**	(no caveat)
distant_metastasis	4,770	4,770	4,063	100.00 %	**85.18 % [84.14, 86.16]**	(no caveat)
cancer_primary_site	2,788	2,788	2,425	100.00 %	**86.98 % [85.68, 88.18]**	(no caveat)
tumor_extent	2,762	2,762	2,406	100.00 %	**87.11 % [85.81, 88.31]**	(no caveat)

dcis_comedo_necrosis	982	982	860	100.00 %	**87.58 % [85.37, 89.49]**	(no caveat)
vascular_invasion	980	980	864	100.00 %	**88.16 % [85.99, 90.04]**	(no caveat)
cancer_clock	982	982	873	100.00 %	**88.90 % [86.78, 90.72]**	(no caveat)
maximal_ln_size	4,770	4,766	4,245	99.92 %	**88.99 % [88.07, 89.85]**	(no caveat)
extranodal_extension	4,770	4,766	4,255	99.92 %	**89.20 % [88.29, 90.05]**	(no caveat)
type_of_polyp	1,006	1,006	898	100.00 %	**89.26 % [87.20, 91.03]**	(no caveat)
signet_ring	1,846	1,846	1,676	100.00 %	**90.79 % [89.39, 92.03]**	(no caveat)
tumor_site	1,000	1,000	912	100.00 %	**91.20 % [89.28, 92.80]**	(no caveat)
extracellular_mucin	1,846	1,846	1,689	100.00 %	**91.50 % [90.13, 92.68]**	(no caveat)
tubule_formation	982	982	899	100.00 %	**91.55 % [89.64, 93.13]**	(no caveat)
total_score	982	982	907	100.00 %	**92.36 % [90.53, 93.86]**	(no caveat)
lymphovascular_invasion	3,790	3,790	3,528	100.00 %	**93.09 % [92.24, 93.85]**	(no caveat)
tumor_focality	1,000	1,000	934	100.00 %	**93.40 % [91.69, 94.78]**	(no caveat)
histology	4,770	4,770	4,501	100.00 %	**94.36 % [93.67, 94.98]**	(no caveat)
tumor_size	1,982	1,982	1,904	100.00 %	**96.06 % [95.12, 96.84]**	(no caveat)
perineural_invasion	4,770	4,770	4,625	100.00 %	**96.96 % [96.43, 97.41]**	(no caveat)
grade	4,750	4,750	4,618	100.00 %	**97.22 % [96.71, 97.65]**	(no caveat)
dcis_grade	982	982	955	100.00 %	**97.25 % [96.03, 98.10]**	(no caveat)
cancer_laterality	982	982	976	100.00 %	**99.39 % [98.67, 99.72]**	(no caveat)
mitotic_activity	20	20	20	100.00 %	**100.00 % [83.87, 100.00]**	(n = 20; small denominator)
tumor_necrosis	20	20	20	100.00 %	**100.00 % [83.87, 100.00]**	(n = 20; small denominator)

tumor_budding	1,006	1,006	1,006	100.00 %	**100.00 % [99.62, 100.00]**	(no caveat)
predisposing_condition	20	20	20	100.00 %	**100.00 % [83.87, 100.00]**	(n = 20; small denominator)

Supplementary Table S39. Baseline cascade comparison — four systems × three stages on the external TCGA cohort.

Stage A / B / C cascade accuracy for four systems on the TCGA-Reports cohort (n = 242 reports across six organs). The four systems span the relevant architectural spectrum: deterministic regex (rule-based), encoder-only fine-tuned (BERT-merged), local LLM (gpt-oss-20b under DSPy with grammar-constrained decoding), and closed-frontier API LLM (gpt-5.4-mini under DSPy with native JSON-schema constrained decoding). Backs Section 3.10.

Stage	Stage name	System	Family	n	Accuracy [95 % Wilson CI]	Cohen's κ
A	Eligibility triage	Rule-based	rule	242	**96.69 % [93.61, 98.32]**	—
A	Eligibility triage	BERT-merged	encoder	2,420	**99.59 % [99.24, 99.78]**	—
A	Eligibility triage	Local LLM (gpt-oss-20b)	local_llm	4,848	**99.59 % [99.36, 99.73]**	—
A	Eligibility triage	API LLM (gpt-5.4-mini)	api_llm	1,452	**99.31 % [98.74, 99.63]**	—
B	Organ classification	Rule-based	rule	234	**96.58 % [93.40, 98.26]**	0.957
B	Organ classification	BERT-merged	encoder	2,410	**66.14 % [64.23, 68.00]**	0.576
B	Organ classification	Local LLM (gpt-oss-20b)	local_llm	4,828	**98.80 % [98.45, 99.07]**	0.985
B	Organ classification	API LLM (gpt-5.4-mini)	api_llm	1,442	**99.31 % [98.73, 99.62]**	0.991
C	Field extraction	Rule-based	rule	1,283	**65.16 % [62.51, 67.72]**	—
C	Field extraction	BERT-merged	encoder	26,205	**40.06 % [39.47, 40.65]**	—
C	Field extraction	Local LLM (gpt-oss-20b)	local_llm	105,853	**77.02 % [76.76, 77.27]**	—
C	Field extraction	API LLM (gpt-5.4-mini)	api_llm	31,200	**75.22 % [74.74, 75.70]**	—

Supplementary Table S40. Baseline cascade-attrition funnel by system.

Per-system attrition through the three cascade gates on the TCGA-Reports cohort (n = 242 reports per system). Reports the proportion of cases that successfully pass each gate (Stage A → Stage B → Stage C). Backs Section 3.10. The Stage-C yield is the fraction of the 242-report cohort that produced a scoreable Stage-C field-extraction output.

System	Family	n_total	n_passed_A	n_passed_B	n_scored_C	Attrition A	Attrition B given A	Stage-C yield
Rule-based	rule	242	234	226	226	3.31 %	3.42 %	**93.39 %**
BERT-merged	encoder	242	241	238	238	0.41 %	1.24 %	**98.35 %**
Local LLM (gpt-oss-20b)	local_llm	242	241	239	239	0.41 %	0.83 %	**98.76 %**
API LLM (gpt-5.4-mini)	api_llm	242	241	240	240	0.41 %	0.41 %	**99.17 %**

Supplementary Table S41. Baseline Stage-C pairwise McNemar tests (Holm-corrected).

Six pairwise McNemar tests on the four baseline systems at Stage C (per-organ field extraction) on the external TCGA-Reports cohort. Each test pairs the two systems on case × field cells where both attempted the same field; n_pairs varies because the systems cover different field-emission regimes. Backs Section 3.10. All six p-values are $< 10^{-9}$; the headline Local-vs-API comparison gives a narrow $\Delta = -3.05$ pp in favour of the local LLM.

Pair (a vs b)	n_pairs	Acc a	Acc b	Δ (b − a)	95 % CI	McNemar p	Verdict
Rule-based vs BERT-merged	1,082	0.6895	0.3005	−0.3889	[−0.4183, −0.3583]	6.5×10^{-84}	rule-based better
Rule-based vs Local LLM (gpt-oss-20b)	1,283	0.6539	0.9042	**+0.2503**	[+0.2263, +0.2750]	3.0×10^{-68}	Local LLM better
Rule-based vs API LLM (gpt-5.4-mini)	1,282	0.6544	0.9028	**+0.2484**	[+0.2223, +0.2733]	1.6×10^{-62}	API LLM better
BERT-merged vs Local LLM (gpt-oss-20b)	3,986	0.3799	0.8160	**+0.4361**	[+0.4174, +0.4534]	1.2×10^{-288}	Local LLM better
BERT-merged vs API LLM (gpt-5.4-mini)	4,001	0.3795	0.7670	**+0.3875**	[+0.3689, +0.4061]	4.3×10^{-236}	API LLM better
Local LLM (gpt-oss-20b) vs API LLM (gpt-5.4-mini)	5,262	0.7976	0.7671	**−0.0305**	[−0.0386, −0.0222]	1.5×10^{-9}	Local LLM better (slight)

Supplementary Table S42. Baseline Stage-C accuracy per organ × system on TCGA.

Per-organ Stage-C accuracy across the four baseline systems on the TCGA-Reports cohort (six organs). Backs Section 3.10. Sorted by organ; values are effective accuracy [95 % Wilson CI].

Organ	Rule-based	BERT-merged	Local LLM (gpt-oss-20b)	API LLM (gpt-5.4-mini)
breast	**56.4 % [51.4, 61.2]** (n=392)	**37.8 % [36.6, 38.9]** (6,779)	**76.6 % [76.1, 77.1]** (29,093)	**76.0 % [75.1, 76.9]** (8,093)
colorectal	**63.1 % [58.2, 67.8]** (n=385)	**44.7 % [43.5, 45.9]** (6,537)	**86.7 % [86.2, 87.2]** (20,144)	**82.4 % [81.4, 83.3]** (6,000)

esophagus	**78.7 % [73.0, 83.4]** (n=239)	**34.2 % [32.9, 35.5]** (4,920)	**83.9 % [83.3, 84.5]** (15,070)	**81.1 % [79.9, 82.2]** (4,625)
liver	**75.6 % [68.3, 81.7]** (n=156)	**49.1 % [47.7, 50.5]** (5,045)	**78.6 % [77.9, 79.2]** (16,660)	**75.2 % [74.0, 76.3]** (4,998)
stomach	**62.5 % [52.5, 71.5]** (n=96)	**29.4 % [27.8, 31.1]** (2,924)	**77.2 % [76.5, 77.8]** (15,114)	**73.4 % [72.1, 74.7]** (4,536)
thyroid	**50.0 % [18.8, 81.2]** (n=6)	— (no cases scored)	**76.6 % [72.1, 80.6]** (380)	**71.1 % [62.1, 78.6]** (114)

Supplementary Table S43. Variable-length list-field F1 (surgical margins, regional lymph-node groups) across baseline systems on TCGA.

List-typed extraction F1 for the two list-typed schema fields (`margins[*]`, `regional_lymph_node[*]`) across the four baseline systems on TCGA-Reports (n = 226 to 1,594 cases per system, depending on field-emission regime). Backs Section 3.10.

Scalar-vs-list divergence — read with Supplementary Table S39. On per-field scalar extraction (Supplementary Table S39) our local language model (gpt-oss-20b) was ahead of the commercial gpt-5.4-mini API (77.02 % vs 75.22 %). On the two nested list fields here, the direction reverses — the commercial API led on both margins (F1 0.831 vs 0.783) and regional lymph-node groups (F1 0.686 vs 0.587). **The local-vs-commercial benchmark was not configured for a balanced head-to-head**: the local model was served with `num_ctx = 16,384` while the commercial API was called with `num_ctx = 4,096` (a 4× asymmetry favouring the local model on long-context inputs), and gpt-5.4-mini is the smaller, faster sibling of a frontier commercial family rather than the full variant. We therefore do not draw a categorical accuracy ordering between local and commercial language models from these numbers. Three discriminating experiments — a matched-context rerun of the commercial API at `num_ctx = 16,384`, a comparison against the full `gpt-5.4`, and an open-weights comparison against `gpt-oss-120b` — are flagged as future work (the GPU budget and time available in this revision did not extend to running them); Section 4 Discussion frames the local-vs-commercial result as a promising follow-up question rather than a categorical accuracy claim.

Task	System	Family	n_total	n_attempted	Coverage	Mean F1 [95 % CI]
margins	Rule-based	rule	226	4	1.77 %	**0.839 [0.625, 1.000]**
regional_lymph_node	Rule-based	rule	226	4	1.77 %	**0.417 [0.000, 0.833]**
margins	BERT-merged	encoder	1,594	0	0.00 %	**— (no list emission)**
regional_lymph_node	BERT-merged	encoder	1,594	0	0.00 %	**— (no list emission)**
margins	Local LLM (gpt-oss-20b)	local_llm	4,770	4,491	94.15 %	**0.783 [0.776, 0.790]**
regional_lymph_node	Local LLM (gpt-oss-20b)	local_llm	4,770	3,967	83.17 %	**0.587 [0.572, 0.601]**
margins	API LLM (gpt-5.4-mini)	api_llm	1,432	1,420	99.16 %	**0.831 [0.820, 0.843]**
regional_lymph_node	API LLM (gpt-5.4-mini)	api_llm	1,432	1,216	84.92 %	**0.686 [0.661, 0.709]**

Supplementary Table S44. Component-ablation 5-method headline.

Five-cell component-ablation headline on the CMUH validation cohort under gpt-oss-20b. The five cells span the proposed pipeline (`dspy_modular`, 10 multi-seed runs) and four single-axis lesions (`dspy_monolithic`, `dspy_monolithic_no_jsonize`, `raw_json`, `free_text_regex`, 1 run each). Total n = 109,618 case × field cells for the modular cell; 10,471–10,953 cells for the four lesion cells (single-run smaller denominator). Backs Section 3.6 (Figure 6).

Method	n_attempted	n_correct	Effective accuracy [95 % Wilson CI]	Δ vs modular	McNemar p (Holm-corrected)
**dspy_modular** (proposed)	109,546	104,678	**95.49 % [95.37, 95.61]**	—	—
raw_json	10,899	10,166	**93.07 % [92.58, 93.53]**	−2.42 pp	1.5×10^{-19}
dspy_monolithic_no_jsonize	10,526	9,998	**91.28 % [90.74, 91.79]**	−4.21 pp	0.0143
dspy_monolithic	10,467	9,925	**90.71 % [90.15, 91.24]**	−4.79 pp	0.0027
free_text_regex	9,690	1,601	**15.29 % [14.61, 15.99]**	−80.20 pp	$< 10^{-300}$

Supplementary Table S45. Component-ablation per-method × per-field accuracy with McNemar Δ.

Per-(method, field) accuracy across the five ablation cells on the CMUH validation cohort. 310 rows = 5 methods × ~62 fields. For each field, the modular cell is the reference; the other four cells report Δ vs modular with paired-bootstrap CIs and McNemar p (note the per-row p is the raw Bonferroni-pre-corrected value; the Holm-corrected aggregate is in Supplementary Table S50).

Because the full 310-row table is unwieldy in a single MD render, the table below renders **one section per method** (excerpt to ~10 representative fields each) plus the aggregate `ALL` row per method.

dspy_modular_gpt_oss_20b — proposed cell (reference)

Field	n	n_correct	n_attempted	Effective accuracy [95 % Wilson CI]	Δ vs modular	McNemar p
anatomic_stage_group	739	724	739	**97.97 % [96.68, 98.77]**	(reference)	—
pathologic_stage_group	629	514	629	**81.72 % [78.51, 84.54]**	(reference)	—
pm_category	1,950	1,774	1,950	**90.98 % [90.26, 91.65]**	(reference)	—
procedure	8,720	8,214	8,720	**94.20 % [93.61, 94.74]**	(reference)	—
histology	4,748	4,701	4,748	**99.01 % [98.74, 99.22]**	(reference)	—
tumor_necrosis	320	203	320	**63.44 % [58.03, 68.53]**	(reference)	—
cancer_quadrant	640	464	640	**72.50 % [68.91, 75.82]**	(reference)	—
surgical_technique	3,863	3,293	3,863	**85.24 % [84.09, 86.33]**	(reference)	—
ALL	109,546	104,678	109,546	**95.49 % [95.37, 95.61]**	(reference)	—

dspy_monolithic_gpt_oss_20b — single-organ-collapsed DSPy with the `ReportJsonize` pre-pass

Field	n	n_correct	Effective accuracy [95 % CI]	Δ vs modular	McNemar p
anatomic_stage_group	74	72	97.30 % [90.85, 99.27]	−0.68 pp	1.000
pathologic_stage_group	62	50	80.65 % [69.10, 88.59]	−1.07 pp	1.000

pm_category	195	176	90.26 % [85.34, 93.65]	−0.72 pp	1.000
procedure	871	815	93.57 % [91.78, 94.97]	−0.62 pp	1.000
tumor_necrosis	32	21	65.62 % [48.34, 79.59]	+2.18 pp	1.000
ALL	10,467	9,925	**90.71 % [90.15, 91.24]**	**−4.79 pp**	**0.0027**

dspy_monolithic_no_jsonize_gpt_oss_20b — single-organ-collapsed DSPy without the `ReportJsonize` pre-pass

Field	n	n_correct	Effective accuracy [95 % CI]	Δ vs modular	McNemar p
anatomic_stage_group	74	70	94.59 % [86.91, 97.88]	−3.39 pp	0.250
pathologic_stage_group	63	60	95.24 % [86.91, 98.37]	+13.52 pp	0.013
pm_category	195	155	79.49 % [73.34, 84.51]	−11.49 pp	1.6×10^{-4}
procedure	871	788	90.47 % [88.36, 92.22]	−3.72 pp	0.001
tumor_necrosis	32	21	65.62 % [48.34, 79.59]	+2.18 pp	1.000
ALL	10,526	9,998	**91.28 % [90.74, 91.79]**	**−4.21 pp**	**0.0143**

raw_json_gpt_oss_20b — schema-constrained JSON without DSPy typing

Field	n	n_correct	Effective accuracy [95 % CI]	Δ vs modular	McNemar p
anatomic_stage_group	74	70	94.59 % [86.91, 97.88]	−3.39 pp	0.250
pathologic_stage_group	63	46	73.02 % [60.91, 82.42]	−8.70 pp	0.057
pm_category	215	174	80.95 % [75.13, 85.69]	−10.04 pp	5.5×10^{-8}
procedure	871	798	91.62 % [89.59, 93.27]	−2.59 pp	0.0006
tumor_necrosis	32	30	93.75 % [79.85, 98.27]	**+30.31 pp**	0.002

ALL	10,899	10,166	**93.07 % [92.58, 93.53]**	**−2.42 pp**	**1.5×10^{-19}**

free_text_regex_gpt_oss_20b — schema-blind regex (the floor)

Field	n	n_correct	Effective accuracy [95 % CI]	Δ vs modular	McNemar p
anatomic_stage_group	74	3	4.05 % [1.39, 11.27]	−93.92 pp	$< 10^{-20}$
pathologic_stage_group	63	5	7.94 % [3.43, 17.34]	−73.78 pp	$< 10^{-15}$
pm_category	195	10	5.13 % [2.82, 9.18]	−85.85 pp	$< 10^{-42}$
procedure	871	62	7.12 % [5.59, 9.04]	−87.08 pp	$< 10^{-150}$
tumor_necrosis	32	8	25.00 % [12.81, 43.43]	−38.44 pp	0.012
ALL	9,690	1,601	**15.29 % [14.61, 15.99]**	**−80.20 pp**	**$< 10^{-300}$**

Supplementary Table S46. Component-ablation per-method × per-organ accuracy with Cochran's Q.

Per-organ accuracy across the five ablation cells on the CMUH validation cohort. Five methods × ten organs = 50 rows. Each row reports n_attempted, n_correct, effective accuracy, 95 % Wilson CI, and the Cochran's Q heterogeneity statistic across organs (per method). Backs Section 3.6 (companion to Supplementary Table S44).

Method	Organ	n_attempted	n_correct	Effective accuracy [95 % Wilson CI]	Cochran's Q	Q-test p
dspy_modular_gpt_oss_20b	breast	15,788	14,979	**94.88 % [94.52, 95.21]**	—	—
dspy_modular_gpt_oss_20b	cervix	3,268	3,088	**94.49 % [93.66, 95.22]**	—	—
dspy_modular_gpt_oss_20b	colorectal	11,730	11,458	**97.68 % [97.39, 97.94]**	—	—
dspy_modular_gpt_oss_20b	esophagus	10,187	9,848	**96.67 % [96.31, 97.00]**	—	—
dspy_modular_gpt_oss_20b	liver	9,870	9,336	**94.59 % [94.13, 95.02]**	—	—
dspy_modular_gpt_oss_20b	lung	9,280	8,830	**95.15 % [94.69, 95.57]**	—	—
dspy_modular_gpt_oss_20b	pancreas	3,840	3,592	**93.54 % [92.72, 94.28]**	—	—
dspy_modular_gpt_oss_20b	prostate	22,550	22,159	**98.27 % [98.09, 98.43]**	—	—
dspy_modular_gpt_oss_20b	stomach	12,515	11,771	**94.06 % [93.63, 94.46]**	—	—
dspy_modular_gpt_oss_20b	thyroid	10,590	9,617	**90.81 % [90.25, 91.35]**	—	—
dspy_monolithic_gpt_oss_20b	breast	1,580	1,347	**85.25 % [83.42, 86.92]**	0.419	0.99999

dspy_monolithic_gpt_oss_20b	cervix	318	282	**88.68 % [84.73, 91.71]**	0.419	0.99999
dspy_monolithic_gpt_oss_20b	colorectal	1,173	1,104	**94.12 % [92.62, 95.33]**	0.419	0.99999
dspy_monolithic_gpt_oss_20b	esophagus	1,003	927	**92.42 % [90.62, 93.90]**	0.419	0.99999
dspy_monolithic_gpt_oss_20b	liver	987	933	**94.53 % [92.93, 95.78]**	0.419	0.99999
dspy_monolithic_gpt_oss_20b	lung	928	774	**83.41 % [80.87, 85.66]**	0.419	0.99999
dspy_monolithic_gpt_oss_20b	pancreas	384	303	**78.91 % [74.55, 82.69]**	0.419	0.99999
dspy_monolithic_gpt_oss_20b	prostate	2,255	2,181	**96.72 % [95.90, 97.38]**	0.419	0.99999
dspy_monolithic_gpt_oss_20b	stomach	1,255	1,099	**87.57 % [85.63, 89.28]**	0.419	0.99999
dspy_monolithic_gpt_oss_20b	thyroid	1,059	975	**92.07 % [90.28, 93.55]**	0.419	0.99999
dspy_monolithic_no_jsonize_gpt_oss_20b	breast	1,580	1,411	**89.30 % [87.68, 90.73]**	0.342	0.99999
dspy_monolithic_no_jsonize_gpt_oss_20b	cervix	329	312	**94.83 % [91.88, 96.75]**	0.342	0.99999
dspy_monolithic_no_jsonize_gpt_oss_20b	colorectal	1,173	1,114	**94.97 % [93.57, 96.08]**	0.342	0.99999
dspy_monolithic_no_jsonize_gpt_oss_20b	esophagus	1,003	936	**93.32 % [91.60, 94.71]**	0.342	0.99999
dspy_monolithic_no_jsonize_gpt_oss_20b	liver	987	925	**93.72 % [92.03, 95.07]**	0.342	0.99999
dspy_monolithic_no_jsonize_gpt_oss_20b	lung	928	783	**84.38 % [81.90, 86.57]**	0.342	0.99999

dspy_monolithic_no_jsonize_gpt_oss_20b	pancreas	384	334	**86.98 % [83.24, 89.98]**	0.342	0.99999
dspy_monolithic_no_jsonize_gpt_oss_20b	prostate	2,255	2,116	**93.84 % [92.77, 94.76]**	0.342	0.99999
dspy_monolithic_no_jsonize_gpt_oss_20b	stomach	1,255	1,084	**86.37 % [84.37, 88.16]**	0.342	0.99999
dspy_monolithic_no_jsonize_gpt_oss_20b	thyroid	1,059	983	**92.82 % [91.11, 94.23]**	0.342	0.99999
free_text_regex_gpt_oss_20b	breast	1,559	238	**15.27 % [13.57, 17.14]**	0.069	0.99999
free_text_regex_gpt_oss_20b	cervix	318	26	**8.18 % [5.64, 11.71]**	0.069	0.99999
free_text_regex_gpt_oss_20b	colorectal	1,121	176	**15.70 % [13.69, 17.95]**	0.069	0.99999
free_text_regex_gpt_oss_20b	esophagus	858	209	**24.36 % [21.61, 27.34]**	0.069	0.99999
free_text_regex_gpt_oss_20b	liver	987	210	**21.28 % [18.84, 23.94]**	0.069	0.99999
free_text_regex_gpt_oss_20b	lung	928	210	**22.63 % [20.05, 25.43]**	0.069	0.99999
free_text_regex_gpt_oss_20b	pancreas	384	49	**12.76 % [9.79, 16.47]**	0.069	0.99999
free_text_regex_gpt_oss_20b	prostate	2,203	120	**5.45 % [4.57, 6.47]**	0.069	0.99999
free_text_regex_gpt_oss_20b	stomach	1,054	109	**10.34 % [8.64, 12.33]**	0.069	0.99999
free_text_regex_gpt_oss_20b	thyroid	1,059	254	**23.98 % [21.51, 26.65]**	0.069	0.99999
raw_json_gpt_oss_20b	breast	1,580	1,318	**83.42 % [81.50, 85.17]**	0.627	0.99992

raw_json_gpt_oss_20b	cervix	318	290	**91.19 % [87.57, 93.84]**	0.627	0.99992
raw_json_gpt_oss_20b	colorectal	1,173	1,133	**96.59 % [95.39, 97.49]**	0.627	0.99992
raw_json_gpt_oss_20b	esophagus	1,005	973	**96.82 % [95.54, 97.74]**	0.627	0.99992
raw_json_gpt_oss_20b	liver	987	929	**94.12 % [92.48, 95.43]**	0.627	0.99992
raw_json_gpt_oss_20b	lung	922	848	**91.97 % [90.04, 93.56]**	0.627	0.99992
raw_json_gpt_oss_20b	pancreas	384	357	**92.97 % [89.96, 95.12]**	0.627	0.99992
raw_json_gpt_oss_20b	prostate	2,255	2,186	**96.94 % [96.15, 97.58]**	0.627	0.99992
raw_json_gpt_oss_20b	stomach	1,240	1,138	**91.77 % [90.11, 93.18]**	0.627	0.99992
raw_json_gpt_oss_20b	thyroid	1,059	994	**93.86 % [92.25, 95.16]**	0.627	0.99992

Supplementary Table S47. Component-ablation marginal means per (field × axis × level).

Marginal accuracies per (field × axis × level), where each axis is a binary engineering choice (modular vs monolithic; jsonize vs no-jsonize; DSPy typing vs raw JSON; schema-constrained vs free-text-regex). 314 rows = ~62 fields × 5 axis levels per field. Used to compute the cross-method effect sizes in Supplementary Tables S44 and S46 after marginalising out the other axes.

The full 314-row long-form is at `code/derived/ablation_marginal_means.csv` and is the canonical numeric source. The summary below reports the four axis aggregates across the `ALL` field (the cross-axis contrast headline).

Aggregate marginal means at the `ALL` (full-schema) level

Axis	Level	n	Marginal accuracy [95 % Wilson CI]
axis_A	modular	109,546	**95.49 % [95.37, 95.61]**
axis_A	non_modular (monolithic + raw_json)	32,318	**89.69 % [89.39, 89.97]**
axis_B	jsonize_on (modular + monolithic + raw_json)	130,907	**94.34 % [94.21, 94.46]**
axis_B	jsonize_off (monolithic_no_jsonize)	10,526	**94.98 % [94.55, 95.38]**
axis_C	dspy_typed (modular + monolithic + monolithic_no_jsonize)	130,539	**94.50 % [94.37, 94.62]**
axis_C	raw_json	10,899	**93.27 % [92.79, 93.73]**
axis_D	schema_constrained (all four except free_text_regex)	141,438	**94.40 % [94.27, 94.51]**
axis_D	free_text_regex	9,690	**16.52 % [15.80, 17.27]**

Supplementary Table S48. Component-ablation factorial GLMM estimates per (field × term).

Per-(field × term) factorial estimates on the logit scale. 250 rows = ~62 fields × 4 terms (intercept, axis_A_modular, axis_B_jsonize, axis_C_dspy_typed). The model is a logistic regression on the binary correctness outcome with the four ablation axes encoded as orthogonal contrasts. Each row reports the field, the term name, the estimate (in logit), the standard error, and the z-statistic.

Field	Term	Estimate (logit)	SE (logit)	z
anatomic_stage_group	(Intercept)	+1.821	0.285	+6.379
anatomic_stage_group	axis_A_modular	+0.902	0.412	+2.190
anatomic_stage_group	axis_B_jsonize	−0.214	0.391	−0.547
anatomic_stage_group	axis_C_dspy_typed	+0.486	0.385	+1.262
pathologic_stage_group	(Intercept)	+1.503	0.198	+7.587
pathologic_stage_group	axis_A_modular	−0.342	0.265	−1.291
pathologic_stage_group	axis_B_jsonize	+0.917	0.281	+3.262
pathologic_stage_group	axis_C_dspy_typed	+0.214	0.258	+0.829
pm_category	(Intercept)	+2.293	0.097	+23.561
pm_category	axis_A_modular	+1.051	0.143	+7.330
pm_category	axis_B_jsonize	−0.067	0.135	−0.495
pm_category	axis_C_dspy_typed	+0.491	0.131	+3.738
procedure	(Intercept)	+2.421	0.062	+38.844
procedure	axis_A_modular	+0.396	0.090	+4.376
procedure	axis_B_jsonize	−0.014	0.084	−0.167
procedure	axis_C_dspy_typed	+0.187	0.082	+2.282
histology	(Intercept)	+4.118	0.131	+31.435
histology	axis_A_modular	+0.572	0.182	+3.149
histology	axis_B_jsonize	−0.034	0.171	−0.199
histology	axis_C_dspy_typed	+0.213	0.164	+1.298
tumor_necrosis	(Intercept)	−0.193	0.354	−0.546
tumor_necrosis	axis_A_modular	−0.587	0.519	−1.131
tumor_necrosis	axis_B_jsonize	+0.452	0.508	+0.890
tumor_necrosis	axis_C_dspy_typed	−1.142	0.480	−2.379
cancer_quadrant	(Intercept)	+0.967	0.085	+11.353
cancer_quadrant	axis_A_modular	+0.014	0.117	+0.121
cancer_quadrant	axis_B_jsonize	−0.078	0.110	−0.711
cancer_quadrant	axis_C_dspy_typed	−0.021	0.106	−0.198
surgical_technique	(Intercept)	+1.736	0.041	+42.336
surgical_technique	axis_A_modular	+0.128	0.058	+2.207
surgical_technique	axis_B_jsonize	−0.045	0.054	−0.838
surgical_technique	axis_C_dspy_typed	+0.096	0.052	+1.842

tnm_descriptor	(Intercept)	+1.683	0.046	+36.797
tnm_descriptor	axis_A_modular	+0.245	0.066	+3.738
tnm_descriptor	axis_B_jsonize	+0.014	0.061	+0.230
tnm_descriptor	axis_C_dspy_typed	+0.087	0.059	+1.479
tumor_extension	(Intercept)	+1.040	0.143	+7.290
tumor_extension	axis_A_modular	−0.204	0.198	−1.030
tumor_extension	axis_B_jsonize	+0.117	0.190	+0.617
tumor_extension	axis_C_dspy_typed	−0.053	0.183	−0.290

Supplementary Table S49. Ablation GLMM with case- and run-level variance decomposition.

Per-(field) generalised linear mixed-effects model estimates for the modular ablation cell, with two-source bootstrap CIs that decompose total uncertainty into case-level heterogeneity (variation across the 893 cases) vs run-level noise (variation across the 10 multi-seeds). 71 fields; the cell column is `dspy_modular` for all rows. Used to attribute residual variance to the underlying source (case-level heterogeneity vs run-to-run model stochasticity). Backs Section 3.6. For brevity below the table renders 12 representative rows spanning the full range of point estimates (high accuracy, mid accuracy, low accuracy)

Field	Field kind	n_cases	n_runs	Point estimate	Mean per-run accuracy	Case CI [lo, hi]	Run CI [lo, hi]	Total CI (two-source bootstrap) [lo, hi]	var_case	var_run
anatomic_stage_group	scalar	75	10	0.9800	0.9800	[0.9653, 0.9866]	[0.9707, 0.9892]	[0.9478, 1.0000]	0.9479	1.0000
biomarker_er	scalar	75	10	1.0000	1.0000	[1.0000, 1.0000]	[1.0000, 1.0000]	[0.9949, 1.0000]	0.9949	1.0000
biomarker_her2	scalar	75	10	1.0000	1.0000	[1.0000, 1.0000]	[1.0000, 1.0000]	[0.9949, 1.0000]	0.9949	1.0000
biomarker_ki67	scalar	75	10	0.9947	0.9946	[0.9853, 0.9973]	[0.9897, 0.9996]	[0.9840, 1.0000]	0.9840	1.0000
biomarker_mlh1	scalar	67	10	1.0000	1.0000	[1.0000, 1.0000]	[1.0000, 1.0000]	[0.9943, 1.0000]	0.9943	1.0000
pathologic_stage_group	scalar	75	10	0.8171	0.8171	[0.7741, 0.8454]	[0.7902, 0.8423]	[0.6747, 0.9325]	0.6747	0.9325
tumor_necrosis	scalar	32	10	0.6344	0.6344	[0.5278, 0.7333]	[0.5849, 0.6797]	[0.5125, 0.7406]	0.5125	0.7406
pm_category	scalar	1,950	10	0.9098	0.9098	[0.9024, 0.9166]	[0.9067, 0.9128]	[0.9006, 0.9192]	0.9006	0.9192
tumor_invasion	scalar	70	10	0.8414	0.8414	[0.8000, 0.8714]	[0.8147, 0.8682]	[0.7857, 0.9000]	0.7857	0.9000
tnm_descriptor	scalar	160	10	0.8421	0.8421	[0.8233, 0.8584]	[0.8331, 0.8511]	[0.8155, 0.8688]	0.8155	0.8688
tumor_size	scalar	379	10	0.9416	0.9416	[0.9335, 0.9485]	[0.9359, 0.9474]	[0.9213, 0.9623]	0.9213	0.9623
visceral_pleural_invasion	scalar	63	10	0.9921	0.9921	[0.9810, 0.9968]	[0.9840, 1.0000]	[0.9778, 1.0000]	0.9778	1.0000

Supplementary Table S50. Modularity advantage per field — modular cell vs best-performing alternative.

For every schema field, the modular cell's accuracy is compared against the best-performing alternative ablation cell on that field. The "modularity advantage" is the modular accuracy minus the best alternative's accuracy, with paired-bootstrap CIs and a Holm-corrected p-value. 63 fields plus the `ALL` aggregate row. Backs Section 3.6.

Sorted ascending by `modular_advantage` so the largest negative-modular and largest positive-modular cells are at the extremes.

Field	Modular accuracy [95 % CI]	Best alternative method	Best alternative accuracy [95 % CI]	Modular advantage [95 % CI]	Holm p
pathologic_stage_group	0.817 [0.785, 0.845]	dspy_monolithic_no_jsonize	0.952 [0.869, 0.984]	**−0.159** [−0.270, −0.048]	0.7505
tumor_necrosis	0.634 [0.580, 0.685]	raw_json	0.938 [0.799, 0.983]	**−0.313** [−0.469, −0.156]	0.1152
perineural_invasion	0.935 [0.929, 0.942]	raw_json	0.977 [0.961, 0.987]	**−0.040** [−0.059, −0.023]	0.0072
distant_metastasis	0.930 [0.923, 0.936]	raw_json	0.955 [0.935, 0.969]	**−0.025** [−0.047, −0.002]	1.0
lymphovascular_invasion	0.966 [0.961, 0.971]	raw_json	0.987 [0.973, 0.994]	**−0.024** [−0.043, −0.006]	1.0
extranodal_extension	0.948 [0.936, 0.957]	raw_json	0.961 [0.922, 0.981]	**−0.011** [−0.050, +0.028]	1.0
intraductal_carcinoma_presence	0.994 [0.986, 0.997]	dspy_monolithic	1.000 [0.961, 1.000]	−0.011 [−0.032, 0.000]	1.0
bladder_invasion	0.990 [0.982, 0.995]	raw_json	1.000 [0.961, 1.000]	−0.011 [−0.032, 0.000]	1.0
gleason_5_percentage	0.983 [0.973, 0.990]	raw_json	0.989 [0.940, 0.998]	−0.011 [−0.056, +0.022]	1.0
tumor_size	0.943 [0.936, 0.950]	raw_json	0.939 [0.909, 0.959]	−0.008 [−0.029, +0.011]	1.0

surgical_technique	0.852 [0.841, 0.863]	dspy_monolithic_no_jsonize	0.850 [0.811, 0.882]	−0.008 [−0.047, +0.031]	1.0
maximal_ln_size	0.976 [0.967, 0.982]	raw_json	0.971 [0.934, 0.988]	−0.006 [−0.041, +0.029]	1.0
tumor_invasion	0.841 [0.813, 0.867]	dspy_monolithic_no_jsonize	0.871 [0.773, 0.931]	−0.014 [−0.086, +0.057]	1.0
tumor_extension	0.739 [0.685, 0.787]	dspy_monolithic_no_jsonize	0.786 [0.605, 0.898]	−0.036 [−0.250, +0.179]	1.0
cancer_quadrant	0.725 [0.689, 0.758]	dspy_monolithic_no_jsonize	0.719 [0.599, 0.814]	−0.016 [−0.125, +0.078]	1.0
cribriform_pattern_presence	0.994 [0.986, 0.997]	dspy_monolithic	1.000 [0.955, 1.000]	0.000 [0.000, 0.000]	1.0
extracellular_mucin	1.000 [0.997, 1.000]	raw_json	1.000 [0.973, 1.000]	0.000 [0.000, 0.000]	1.0
extraprostatic_extension	1.000 [0.996, 1.000]	dspy_monolithic	1.000 [0.961, 1.000]	0.000 [0.000, 0.000]	1.0
extrathyroid_extension	0.978 [0.944, 0.991]	dspy_monolithic_no_jsonize	1.000 [0.824, 1.000]	0.000 [0.000, 0.000]	1.0
margin_length	0.990 [0.975, 0.996]	dspy_monolithic	1.000 [0.914, 1.000]	0.000 [0.000, 0.000]	1.0
margin_positivity	0.996 [0.989, 0.998]	raw_json	1.000 [0.961, 1.000]	0.000 [0.000, 0.000]	1.0
overall_stage	0.978 [0.970, 0.984]	raw_json	0.972 [0.936, 0.988]	0.000 [−0.034, +0.034]	1.0
prostate_size	0.993 [0.985, 0.996]	dspy_monolithic	0.989 [0.942, 0.998]	0.000 [−0.032, +0.032]	1.0
prostate_weight	1.000 [0.996, 1.000]	dspy_monolithic	1.000 [0.960, 1.000]	0.000 [0.000, 0.000]	1.0

seminal_vesicle_invasion	1.000 [0.996, 1.000]	dspy_monolithic	1.000 [0.961, 1.000]	0.000 [0.000, 0.000]	1.0
signet_ring	1.000 [0.997, 1.000]	raw_json	1.000 [0.974, 1.000]	0.000 [0.000, 0.000]	1.0
spread_through_air_spaces_stas	0.980 [0.964, 0.989]	raw_json	1.000 [0.930, 1.000]	0.000 [0.000, 0.000]	1.0
tubule_formation	1.000 [0.995, 1.000]	dspy_monolithic_no_jsonize	0.959 [0.886, 0.986]	+0.041 [0.000, +0.096]	1.0
visceral_pleural_invasion	0.991 [0.978, 0.996]	raw_json	1.000 [0.932, 1.000]	0.000 [0.000, 0.000]	1.0
tumor_budding	1.000 [0.994, 1.000]	dspy_monolithic	1.000 [0.946, 1.000]	0.000 [0.000, 0.000]	1.0
type_of_polyp	0.991 [0.969, 0.998]	raw_json	1.000 [0.857, 1.000]	0.000 [0.000, 0.000]	1.0
depth_of_invasion_number	1.000 [0.986, 1.000]	dspy_monolithic_no_jsonize	1.000 [0.875, 1.000]	0.000 [0.000, 0.000]	1.0
depth_of_invasion_three_tier	0.952 [0.915, 0.974]	raw_json	0.952 [0.773, 0.992]	0.000 [−0.143, +0.143]	1.0
direct_invasion_of_adjacent_structures	0.967 [0.947, 0.979]	raw_json	0.958 [0.860, 0.988]	+0.021 [−0.042, +0.104]	1.0
pn_category	0.963 [0.958, 0.967]	raw_json	0.949 [0.929, 0.963]	+0.011 [−0.009, +0.029]	1.0
cancer_clock	0.915 [0.884, 0.938]	dspy_monolithic	0.854 [0.716, 0.931]	0.000 [−0.098, +0.098]	1.0
vascular_invasion	0.921 [0.874, 0.952]	dspy_monolithic_no_jsonize	0.947 [0.754, 0.991]	0.000 [−0.158, +0.158]	1.0
tumor_size	0.943 [0.936, 0.950]	raw_json	0.939 [0.909, 0.959]	−0.008 [−0.029, +0.011]	1.0

total_score	1.000 [0.995, 1.000]	dspy_monolithic_no_jsonize	0.945 [0.867, 0.978]	+0.055 [+0.014, +0.110]	1.0
nuclear_grade	1.000 [0.995, 1.000]	dspy_monolithic_no_jsonize	0.959 [0.886, 0.986]	+0.041 [0.000, +0.096]	1.0
mitotic_rate	1.000 [0.995, 1.000]	dspy_monolithic_no_jsonize	0.959 [0.886, 0.986]	+0.041 [0.000, +0.096]	1.0
mitotic_activity	0.969 [0.953, 0.980]	dspy_monolithic_no_jsonize	0.968 [0.890, 0.991]	+0.016 [−0.032, +0.081]	1.0
dcis_size	1.000 [0.992, 1.000]	dspy_monolithic_no_jsonize	0.960 [0.865, 0.989]	+0.040 [0.000, +0.100]	1.0
dcis_present	0.992 [0.982, 0.996]	dspy_monolithic_no_jsonize	0.946 [0.869, 0.979]	+0.041 [0.000, +0.095]	1.0
dcis_grade	0.986 [0.972, 0.993]	dspy_monolithic_no_jsonize	0.961 [0.868, 0.989]	+0.020 [−0.039, +0.098]	1.0
dcis_comedo_necrosis	0.973 [0.954, 0.984]	dspy_monolithic	0.882 [0.766, 0.945]	**+0.078** [0.000, +0.176]	1.0
anatomic_stage_group	0.980 [0.967, 0.988]	dspy_monolithic_no_jsonize	0.946 [0.869, 0.979]	+0.041 [0.000, +0.095]	1.0
cancer_laterality	0.991 [0.981, 0.995]	free_text_regex	0.973 [0.907, 0.993]	+0.027 [0.000, +0.068]	1.0
sideness	0.998 [0.991, 1.000]	free_text_regex	0.984 [0.915, 0.997]	+0.016 [0.000, +0.048]	1.0
tumor_focality	0.979 [0.972, 0.984]	dspy_monolithic_no_jsonize	0.954 [0.917, 0.975]	**+0.028** [+0.005, +0.056]	1.0
tumor_site	0.947 [0.936, 0.957]	dspy_monolithic_no_jsonize	0.944 [0.900, 0.969]	+0.017 [−0.011, +0.045]	1.0
pt_category	0.998 [0.997, 0.999]	raw_json	0.992 [0.982, 0.997]	+0.006 [+0.002, +0.012]	1.0

stage_group	0.958 [0.951, 0.964]	raw_json	0.928 [0.897, 0.949]	**+0.026** [+0.003, +0.052]	1.0
tumor_extent	0.976 [0.967, 0.983]	raw_json	0.935 [0.885, 0.964]	**+0.045** [+0.006, +0.084]	1.0
histology	0.990 [0.987, 0.992]	raw_json	0.979 [0.965, 0.987]	**+0.015** [+0.005, +0.026]	0.750 5
grade	0.999 [0.998, 0.999]	raw_json	0.991 [0.979, 0.996]	+0.009 [+0.002, +0.018]	1.0
gleason_4_percentage	0.971 [0.958, 0.980]	dspy_monolithic	0.956 [0.892, 0.983]	+0.011 [0.000, +0.033]	1.0
cancer_primary_site	0.918 [0.907, 0.927]	raw_json	0.928 [0.892, 0.953]	+0.004 [−0.032, +0.036]	1.0
tumor_percentage	0.974 [0.962, 0.983]	dspy_monolithic	0.957 [0.895, 0.983]	+0.011 [−0.022, +0.054]	1.0
procedure	0.942 [0.936, 0.947]	raw_json	0.916 [0.893, 0.935]	**+0.035** [+0.017, +0.053]	0.035 4
pm_category	0.910 [0.903, 0.917]	raw_json	0.810 [0.778, 0.838]	**+0.096** [+0.062, +0.128]	3.4×10^{-6}
involved_margin_list	0.863 [0.827, 0.893]	dspy_monolithic	0.829 [0.687, 0.915]	+0.024 [−0.073, +0.123]	1.0
tnm_descriptor	0.842 [0.823, 0.859]	dspy_monolithic_no_jsonize	0.806 [0.738, 0.860]	+0.037 [−0.012, +0.081]	1.0
**ALL (aggregate)**	**0.955 [0.954, 0.956]**	raw_json	**0.931 [0.926, 0.935]**	**+0.024 [+0.019, +0.029]**	**3.5×10^{-19}**

Supplementary Table S51. Ablation modular-cell seed consistency (Fleiss κ across 10 seeds).

For every schema field in the modular cell (`dspy_modular_gpt_oss_20b`), the per-seed accuracy mean / SD / min / max across the 10 multi-seed runs of the modular cell, plus Fleiss κ across the 10 raters (seeds). Used to bound the seed-to-seed variability that the single-run estimates on the four alternative ablation cells conceal. 62 fields. Backs Section 3.6; the seed-count rationale and the within-axis matched-pair logic this table feeds are

documented in Supplementary Sections Matched-pair effects the grid can and cannot isolate and Seed-count asymmetry across cells.

Field	n_seeds	Accuracy mean	Accuracy SD	Accuracy min	Accuracy max	Fleiss κ across seeds
anatomic_stage_group	10	0.9797	0.0124	0.9459	0.9865	0.6116
bladder_invasion	10	0.9904	0.0074	0.9787	1.0000	0.4391
cancer_clock	10	0.9146	0.0273	0.8537	0.9512	0.6078
cancer_laterality	10	0.9907	0.0085	0.9733	1.0000	0.4712
cancer_primary_site	10	0.9177	0.0122	0.8968	0.9364	0.6160
cancer_quadrant	10	0.7250	0.0281	0.6719	0.7656	0.5733
cribriform_pattern_presence	10	0.9938	0.0062	0.9877	1.0000	0.4410
dcis_comedo_necrosis	10	0.9725	0.0180	0.9412	1.0000	0.4614
dcis_grade	10	0.9863	0.0090	0.9804	1.0000	0.2114
dcis_present	10	0.9919	0.0066	0.9865	1.0000	0.4406
dcis_size	10	1.0000	0.0000	1.0000	1.0000	— (constant)
depth_of_invasion_number	10	1.0000	0.0000	1.0000	1.0000	— (constant)
depth_of_invasion_three_tier	10	0.9524	0.0301	0.9048	1.0000	0.2067
direct_invasion_of_adjacent_structures	10	0.9667	0.0191	0.9375	1.0000	0.1954
distant_metastasis	10	0.9296	0.0051	0.9189	0.9371	0.4428
extracellular_mucin	10	1.0000	0.0000	1.0000	1.0000	— (constant)
extranodal_extension	10	0.9476	0.0090	0.9333	0.9663	0.7054
extraprostatic_extension	10	1.0000	0.0000	1.0000	1.0000	— (constant)
extrathyroid_extension	10	0.9778	0.0272	0.9444	1.0000	0.0341
gleason_4_percentage	10	0.9714	0.0088	0.9560	0.9780	0.7976
gleason_5_percentage	10	0.9833	0.0124	0.9556	1.0000	0.4350
grade	10	0.9989	0.0015	0.9964	1.0000	0.0730
histology	10	0.9901	0.0024	0.9864	0.9940	0.4280
intraductal_carcinoma_presence	10	0.9936	0.0071	0.9787	1.0000	0.2545
involved_margin_list	10	0.8634	0.0365	0.8293	0.9512	0.6737
lymphovascular_invasion	10	0.9661	0.0036	0.9591	0.9704	0.4437
margin_length	10	0.9902	0.0195	0.9512	1.0000	0.1024
margin_positivity	10	0.9957	0.0085	0.9787	1.0000	0.1073
maximal_ln_size	10	0.9755	0.0050	0.9651	0.9826	0.5659
mitotic_activity	10	0.9694	0.0168	0.9355	0.9839	0.4751
mitotic_rate	10	1.0000	0.0000	1.0000	1.0000	— (constant)
nuclear_grade	10	1.0000	0.0000	1.0000	1.0000	— (constant)
overall_stage	10	0.9782	0.0104	0.9553	0.9944	0.4554

pathologic_stage_group	10	0.8171	0.0282	0.7742	0.8730	0.2324
perineural_invasion	10	0.9355	0.0023	0.9318	0.9397	0.6011
pm_category	10	0.9098	0.0097	0.8908	0.9276	0.3430
pn_category	10	0.9631	0.0052	0.9555	0.9708	0.4672
procedure	10	0.9420	0.0085	0.9246	0.9532	0.6703
prostate_size	10	0.9926	0.0068	0.9787	1.0000	0.1844
prostate_weight	10	1.0000	0.0000	1.0000	1.0000	— (constant)
pt_category	10	0.9982	0.0012	0.9969	1.0000	0.3695
seminal_vesicle_invasion	10	1.0000	0.0000	1.0000	1.0000	— (constant)
sideness	10	0.9984	0.0048	0.9841	1.0000	−0.0016
signet_ring	10	1.0000	0.0000	1.0000	1.0000	— (constant)
spread_through_air_spaces_stas	10	0.9804	0.0175	0.9608	1.0000	0.1160
stage_group	10	0.9579	0.0072	0.9432	0.9667	0.5897
surgical_technique	10	0.8525	0.0186	0.8243	0.8961	0.5941
tnm_descriptor	10	0.8421	0.0104	0.8302	0.8571	0.8248
total_score	10	1.0000	0.0000	1.0000	1.0000	— (constant)
tubule_formation	10	1.0000	0.0000	1.0000	1.0000	— (constant)
tumor_budding	10	1.0000	0.0000	1.0000	1.0000	— (constant)
tumor_extension	10	0.7393	0.0393	0.6786	0.7857	0.4832
tumor_extent	10	0.9763	0.0070	0.9618	0.9873	0.7261
tumor_focality	10	0.9787	0.0052	0.9676	0.9861	0.8075
tumor_invasion	10	0.8414	0.0251	0.8000	0.8857	0.5253
tumor_necrosis	10	0.6344	0.0626	0.5313	0.7188	0.3219
tumor_percentage	10	0.9742	0.0086	0.9570	0.9892	0.5913
tumor_site	10	0.9475	0.0107	0.9274	0.9609	0.6207
tumor_size	10	0.9434	0.0073	0.9307	0.9520	0.7032
type_of_polyp	10	0.9913	0.0174	0.9565	1.0000	0.1033
vascular_invasion	10	0.9211	0.0424	0.8421	1.0000	0.2842
visceral_pleural_invasion	10	0.9906	0.0127	0.9623	1.0000	−0.0095

Supplementary Table S52. Component-ablation efficiency stats (schema-error rate, parse-error rate, latency).

Per-cell efficiency stats across the five ablation cells (deduplicated from the source CSV which lists the modular cell once per seed). Reports schema-error rate, parse-error rate, and median per-case latency with bootstrap CIs. Backs Section 3.6.

Cell	Model	n_cases	Schema-error rate [95 % Wilson CI]	Parse-error rate [95 % CI]	Median latency (s) [95 % BCa CI]	Mean latency (s)
dspy_modular	gpt_oss_20b	893	**0.00 % [0.00, 0.43]**	**0.00 % [0.00, 0.43]**	(not reported per seed; aggregate latency in the underlying eval CSVs)	—
dspy_monolithic	gpt_oss_20b	893	**0.00 % [0.00, 0.43]**	**3.36 % [2.36, 4.76]**	28.46 [27.49, 29.48]	26.96
dspy_monolithic_no_jsonize	gpt_oss_20b	893	**0.00 % [0.00, 0.43]**	**2.80 % [1.90, 4.10]**	15.00 [14.23, 15.52]	15.14
free_text_regex	gpt_oss_20b	893	**0.00 % [0.00, 0.43]**	**0.00 % [0.00, 0.43]**	2.94 [2.89, 3.00]	3.50
raw_json	gpt_oss_20b	893	**61.37 % [58.13, 64.50]**	**0.00 % [0.00, 0.43]**	21.83 [20.89, 22.51]	23.78

Supplementary Table S53. Ablation low-performer diagnostics — modular cell, fields with accuracy < 90 %.

Eight fields from the modular ablation cell (`dspy_modular_gpt_oss_20b`) with effective accuracy < 0.90; for each, the residual error is decomposed into model-silent (`field_missing_rate`) vs attempted-wrong (`wrong_when_attempted_rate`). Backs Section 3.6.

Field	n	Effective accuracy [95 % Wilson CI]	n_correct	n_wrong	n_field_missing	n_parse_error	Field-missing rate	Wrong-when-attempted rate
tumor_necrosis	320	**63.44 % [58.03, 68.53]**	203	117	0	0	0.00 %	36.56 %
cancer_quadrant	640	**72.50 % [68.91, 75.82]**	464	176	0	0	0.00 %	27.50 %
tumor_extension	280	**73.93 % [68.49, 78.72]**	207	73	0	0	0.00 %	26.07 %
pathologic_stage_group	629	**81.72 % [78.51, 84.54]**	514	115	0	0	0.00 %	18.28 %
tumor_invasion	700	**84.14 % [81.25, 86.66]**	589	111	0	0	0.00 %	15.86 %
tnm_descriptor	1,602	**84.21 % [82.34, 85.91]**	1,349	253	0	0	0.00 %	15.79 %
surgical_technique	3,863	**85.24 % [84.09, 86.33]**	3,293	570	0	0	0.00 %	14.76 %
involved_margin_list	410	**86.34 % [82.68, 89.33]**	354	52	4	0	0.98 %	12.81 %

Supplementary Table S54. Component-ablation accuracy by field-type and by cascade chapter.

Five-variant component-ablation accuracy stratified by chapter-3 scalar field type (boolean / rigid enum / numerical) and by the two cascade chapters that score variable-length lists of structured objects (chapter 4 surgical margins, chapter 5 lymph-node groups). Backs Section 3.6 / paragraph 14 and rebuttal R1c. The four field-type categories are defined below. The per-chapter list-level scoring rule (greedy canonical-category alignment then per-element exact-match) is defined in Section 2.4.1.

Each chapter-3 field-type row pools n_correct / n_attempted across the fields in that category and reports a Wilson 95 % CI on the pooled denominator. Each chapter-4 / chapter-5 row pools tp / fp / fn across the 10 organs and recomputes micro F1, hallucination rate = fp / (tp + fp), and miss rate = fn / (tp + fn) on the pooled counts. McNemar p-values across variants within a row require case-level matching data not available at the chapter-pooled level; per-(variant × field) significance for chapter-3 fields is at Supplementary Table S45.

S54a — Chapter-3 field-type accuracy (all five variants)

Variant	Boolean (20 fields, n)	Δ vs modular	Rigid enum (32 fields, n)	Δ vs modular	Numerical (9 fields, n)	Δ vs modular
**dspy_modular** (proposed)	**96.46 %** (25 121)	—	**95.06 %** (73 296)	—	**97.17 %** (10 723)	—
dspy_monolithic	97.64 % (2 416)	+1.18 pp	93.66 % (6 964)	**−1.40 pp**	96.56 % (1 046)	−0.62 pp
dspy_monolithic_no_jsonize	97.63 % (2 408)	+1.18 pp	93.84 % (7 042)	−1.22 pp	97.01 % (1 038)	−0.16 pp
raw_json	97.69 % (2 508)	+1.23 pp	91.38 % (7 278)	**−3.67 pp**	96.64 % (1 072)	−0.53 pp
free_text_regex	**5.79 %** (2 314)	**−90.67 pp**	**19.17 %** (6 939)	**−75.89 pp**	**31.35 %** (437)	**−65.82 pp**

S54b — Chapter 4 (surgical margins) list-level micro F1

Variant	n cases (10 organs)	Micro F1 [95 % bootstrap CI]	Hallucination rate (fp / (tp+fp))	Miss rate (fn / (tp+fn))	Δ F1 vs modular
**dspy_modular** (proposed)	5 659 / 6 636	**88.39 %**	18.73 %	3.12 %	—
dspy_monolithic	539 / 662	**92.80 %**	10.56 %	3.58 %	**+4.41 pp**
dspy_monolithic_no_jsonize	548 / 663	92.24 %	11.07 %	4.19 %	+3.85 pp
raw_json	566 / 660	87.39 %	11.56 %	13.64 %	−1.00 pp
free_text_regex	0 / 633	undefined (n_attempted = 0)	—	—	(regex extractor produces no list emissions)

S54c — Chapter 5 (lymph-node groups) list-level micro F1 (the load-bearing observation)

Variant	n cases (10 organs)	Micro F1 [95 % bootstrap CI]	Hallucination rate (fp / (tp+fp))	Miss rate (fn / (tp+fn))	Δ F1 vs modular
**dspy_modular** (proposed)	6 568 / 6 636	**77.68 %**	10.21 %	31.55 %	—
dspy_monolithic	633 / 662	**86.67 %**	11.19 %	15.38 %	**+8.98 pp**
dspy_monolithic_no_jsonize	639 / 663	85.67 %	10.90 %	17.50 %	+7.99 pp
**raw_json**	659 / 660	**46.12 %**	**52.50 %**	**55.17 %**	**−31.56 pp**
free_text_regex	0 / 633	undefined (n_attempted = 0)	—	—	(regex extractor produces no list emissions)

S54d — Per-organ chapter-5 F1 (where `raw_json` collapses)

Per-organ breakdown showing that the chapter-5 −31.56 pp pooled penalty for `raw_json` is concentrated in four organs (cervix, esophagus, pancreas, thyroid)

Organ	Proposed (modular) F1	`raw_json` F1	Δ pp F1	`raw_json` hallucination	`raw_json` miss
colorectal	96.9 %	92.9 %	−3.9	6.7 %	7.4 %
liver	95.7 %	75.0 %	−20.7	14.3 %	33.3 %
stomach	91.1 %	56.0 %	−35.0	45.3 %	42.6 %
breast	88.7 %	68.8 %	−19.9	24.7 %	36.8 %
lung	81.1 %	80.4 %	−0.7	16.4 %	22.6 %
pancreas	79.0 %	9.3 %	**−69.7**	**90.7 %**	**90.8 %**
thyroid	72.8 %	23.6 %	**−49.2**	**75.5 %**	**77.2 %**
prostate	71.5 %	74.7 %	+3.2	18.0 %	31.4 %
cervix	62.8 %	undefined (P=R=0)	**collapse**	**100.0 %**	**100.0 %**
esophagus	57.3 %	0.7 %	**−56.7**	**99.3 %**	**99.4 %**

S54e — Per-organ chapter-4 F1 (for comparison)

Per-organ chapter-4 detail confirming that the chapter-4 picture is benign across all variants — the prostate row is `n_attempted = 0` because the prostate schema does not score margins in the same list form.

Organ	Proposed (modular) F1	`raw_json` F1	Δ pp F1	`raw_json` hallucination	`raw_json` miss
breast	97.3 %	93.5 %	−3.8	3.0 %	9.7 %
colorectal	96.8 %	95.6 %	−1.1	3.0 %	5.8 %
pancreas	93.0 %	87.0 %	−6.0	10.7 %	15.2 %
esophagus	90.8 %	84.5 %	−6.3	3.5 %	24.9 %
cervix	89.9 %	83.5 %	−6.5	9.4 %	22.7 %

stomach	84.4 %	86.8 %	+2.3	21.7 %	2.7 %
thyroid	80.4 %	81.9 %	+1.5	18.6 %	17.5 %
liver	73.5 %	63.3 %	−10.1	40.2 %	32.7 %
lung	62.1 %	84.2 %	+22.1	24.8 %	4.5 %

Reading the table together

The dominant single observation across all five variants is the **chapter-5 −31.56 pp F1 collapse on `raw_json`**, with hallucination and miss rates both exceeding 50 %. This single penalty (1 of 25 cells in S54a–c) is larger in absolute pp than all chapter-3 cells from the three DSPy-or-JSON variants combined. Every other comparison either favours the proposed pipeline modestly (rigid-enum fields, schema-blind floors) or favours alternatives modestly (chapter 4 by 4 pp, chapter 5 LN under DSPy-monolithic by 9 pp). The schema-blind `free_text_regex` floor collapses uniformly across chapter 3 (66–91 pp) and produces zero list emissions for chapters 4 and 5. The seed-count asymmetry (1-seed alternatives vs 10-seed modular) caveats the chapter-4 numbers in particular; a cleaner factorial sweep with matched seed counts is in Supplementary Section Honest scope: what the grid cannot reach.

Source CSVs: code/derived/ablation_stage_stratified.csv, code/derived/ablation_nested_list_pooled.csv, code/derived/ablation_nested_list_chapter4.csv, code/derived/ablation_nested_list_chapter5.csv.

Supplementary Table S55. IAA headline Cohen's κ per field — operational annotator pair (KPC × NHC, both with LLM seed).

Per-field Cohen's κ on the operational annotator pair (`nhc_with_preann × kpc_with_preann` — the pair the manuscript actually deploys). 41 categorical fields plus the `__WEIGHTED_MEAN__` summary. Backs Section 3.7 and Figure 7 (per-field κ bars sorted ascending). The headline weighted-mean κ = 0.844 across n = 9,598 case × field pairings.

Field	Field type	Section	Cohen's κ [95 % CI]	Observed agreement	n_categories	n_cases
`__WEIGHTED_MEAN__` (summary)	summary	summary	**0.8438**	—	—	9,598
cancer_category	nominal	top_level	**0.9362 [0.9189, 0.9500]**	0.9429	12	893
cancer_excision_report	nominal	top_level	0.8775 [0.8376, 0.9129]	0.9586	2	893
extranodal_extension	binary	scalar_pathology	0.8773 [0.8352, 0.9132]	0.9449	3	653
distant_metastasis	binary	scalar_pathology	**0.0578 [0.0368, 0.0786]**	0.1807	3	653

histology	nominal	scalar_patholog y	0.9865 [0.9762, 0.9949]	0.9877	36	653
tnm_descriptor	nominal	scalar_patholog y	0.7583 [0.7031, 0.8071]	0.8989	4	653
procedure	nominal	scalar_patholog y	0.9346 [0.9148, 0.9525]	0.9387	38	653
perineural_invasion	binary	scalar_patholog y	0.9256 [0.8954, 0.9489]	0.9583	3	624
lymphovascular_invasion	binary	scalar_patholog y	0.9778 [0.9522, 0.9912]	0.9908	3	545
surgical_technique	nominal	scalar_patholog y	0.7972 [0.7560, 0.8356]	0.8396	8	399
cancer_primary_site	nominal	scalar_patholog y	0.9078 [0.8725, 0.9397]	0.9148	23	305
tumor_extent	nominal	scalar_patholog y	0.9896 [0.9683, 1.0000]	0.9913	16	231
tumor_focality	nominal	scalar_patholog y	0.9670 [0.9208, 0.9916]	0.9805	6	205
tumor_site	nominal	scalar_patholog y	0.8685 [0.8050, 0.9157]	0.9050	10	179
extracellular_mucin	binary	scalar_patholog y	**1.0000 [1.0000, 1.0000]**	1.0000	3	148
signet_ring	binary	scalar_patholog y	**1.0000 [1.0000, 1.0000]**	1.0000	3	148
cribriform_pattern_presence	binary	scalar_patholog y	0.9459 [0.8626, 0.9830]	0.9681	3	94
margin_length	nominal	scalar_patholog y	0.9819 [0.8943, 1.0000]	0.9894	3	94
intraductal_carcinoma_presence	binary	scalar_patholog y	**1.0000 [1.0000, 1.0000]**	1.0000	2	94

seminal_vesicle_invasion	binary	scalar_pathology	**1.0000 [1.0000, 1.0000]**	1.0000	2	94
extraprostatic_extension	binary	scalar_pathology	**1.0000 [1.0000, 1.0000]**	1.0000	2	94
bladder_invasion	binary	scalar_pathology	**1.0000 [1.0000, 1.0000]**	1.0000	2	94
margin_positivity	binary	scalar_pathology	**1.0000 [1.0000, 1.0000]**	1.0000	2	94
dcis_present	binary	scalar_pathology	0.9392 [0.7950, 1.0000]	0.9733	3	75
dcis_comedo_necrosis	binary	scalar_pathology	0.9588 [0.8725, 1.0000]	0.9733	3	75
cancer_clock	nominal	scalar_pathology	0.7369 [0.6072, 0.8449]	0.8000	13	75
cancer_laterality	nominal	scalar_pathology	0.9737 [0.8984, 1.0000]	0.9867	3	75
cancer_quadrant	nominal	scalar_pathology	0.5555 [0.4184, 0.6894]	0.6533	8	75
predisposing_condition	nominal	scalar_pathology	— (constant)	1.0000	1	72
tumor_necrosis	binary	scalar_pathology	0.8314 [0.6446, 0.9387]	0.9167	2	72
mitotic_activity	nominal	scalar_pathology	0.9560 [0.7500, 1.0000]	0.9861	3	72
extrathyroid_extension	nominal	scalar_pathology	0.9647 [0.8168, 1.0000]	0.9861	4	72
tumor_invasion	nominal	scalar_pathology	0.8576 [0.7482, 0.9397]	0.9000	5	70
type_of_polyp	nominal	scalar_pathology	**1.0000 [1.0000, 1.0000]**	1.0000	5	70

visceral_pleural_invasion	binary	scalar_patholog y	0.9464 [0.6986, 1.0000]	0.9815	3	54
spread_through_air_spaces_stas	binary	scalar_patholog y	0.7662 [0.4375, 0.9392]	0.9259	3	54
direct_invasion_of_adjacent_structure s	binary	scalar_patholog y	0.5277 [0.1608, 0.8414]	0.8889	3	54
sideness	nominal	scalar_patholog y	**1.0000 [1.0000, 1.0000]**	1.0000	2	54
depth_of_invasion_number	nominal	scalar_patholog y	**1.0000 [1.0000, 1.0000]**	1.0000	4	29
depth_of_invasion_three_tier	nominal	scalar_patholog y	0.7143 [0.4980, 0.8599]	0.7931	4	29
tumor_extension	nominal	scalar_patholog y	0.6536 [0.3554, 0.8716]	0.7857	3	28

Supplementary Table S56. IAA 8-pair × per-(organ, field) Cohen's κ matrix.

Full per-(pair × organ × field × stat_name) IAA detail across the 8 annotator-comparison pairs that the Section 3.7 / R1.b analysis covers. The eight pairs are the cross-product of {`gold`, `kpc`, `nhc`} × {`with_preann`, `without_preann`} arms (subset to the seven well-defined comparisons), plus the operational pair `nhc_with_preann × kpc_with_preann` that drives the Section 3.7 headline.

The full long-form CSV is **16,448 rows** — one per (pair, stat_name, organ, field) tuple. Direct rendering of all 16k rows in Markdown would be unwieldy; this MD instead renders **the per-pair × per-organ aggregates** (≈ 200 rows) for the headline `cohen_kappa` statistic, and links to the full long-form CSV for row-level inspection.

The eight pairs:

1. `gold_vs_kpc_with_preann` — gold vs KPC, with-LLM-seed arm.
2. `gold_vs_kpc_without_preann` — gold vs KPC, without-LLM-seed arm.
3. `gold_vs_nhc_with_preann` — gold vs NHC, with-LLM-seed arm.
4. `gold_vs_nhc_without_preann` — gold vs NHC, without-LLM-seed arm.
5. `nhc_with_preann_vs_nhc_without_preann` — within-annotator (NHC) seed-effect.
6. `kpc_with_preann_vs_kpc_without_preann` — within-annotator (KPC) seed-effect.
7. `nhc_with_preann_vs_kpc_with_preann` — **operational pair**, both arms with seed (the source of the Section 3.7 headline).
8. `nhc_without_preann_vs_kpc_without_preann` — between-annotator without-seed cross-check.

Operational pair (nhc_with_preann × kpc_with_preann) per-organ Cohen's κ aggregates

Organ	Cohen's κ across all in-organ fields
breast	0.91 (averaged across breast fields, weighted by per-field n)
cervix	0.85
colorectal	0.93
esophagus	0.86
liver	0.92
lung	0.84
pancreas	0.79
prostate	0.96
stomach	0.84
thyroid	0.93
**All organs**	**0.844** (the Section 3.7 headline weighted mean)

Pairwise headline (whole-cohort weighted-mean Cohen's κ)

Pair	Cohen's κ aggregate	n (case × field pairings)	Notes
gold_vs_kpc_with_preann	0.96	9,553	Reference / KPC-with-seed arm; high

			because gold is committee-resolved
gold_vs_kpc_without_preann	0.96	1,962	KPC-without-seed arm; consistent with seeded arm
gold_vs_nhc_with_preann	0.97	9,604	NHC-with-seed; consistent with KPC-with-seed
gold_vs_nhc_without_preann	0.94	1,956	NHC-without-seed; mildly lower than seeded arm
nhc_with_preann_vs_nhc_without_preann	0.93	2,014	Within-annotator (NHC) seed-effect
kpc_with_preann_vs_kpc_without_preann	0.95	1,988	Within-annotator (KPC) seed-effect
**nhc_with_preann_vs_kpc_with_preann (operational)**	**0.844**	**9,598**	**Section 3.7 headline**
nhc_without_preann_vs_kpc_without_preann	0.93	1,973	Between-annotator without-seed cross-check

Within-pair per-field range

For the operational pair, the per-(organ, field) κ values range from a low of **0.058** (`distant_metastasis` — κ paradox under class imbalance, see Supplementary Table S55 NOTE 3) to a high of **1.000** (multiple deterministic fields where both annotators always agree). The full range per pair × organ × field is in the long-form CSV.

Supplementary Table S57. IAA whole-report metrics — Krippendorff's α and case-level exact-match rate across 8 pairs.

For each of the 8 annotator-comparison pairs (see Supplementary Table S56 for the pair definitions), four whole-report-level metrics: case-level exact-match rate (with Wilson 95 % CI) and Krippendorff's α at three measurement levels (nominal, ordinal, interval). The Krippendorff α metrics are reported without CIs because the source pipeline does not bootstrap them. Backs Section 3.7 / paragraph 15 (the Krippendorff α cross-check sentence) and Supplementary Figure S3.

Pair	Stat	Estimate	95 % Wilson CI [lo, hi]	n
gold_vs_kpc_with_preann	case_exact_match_rate	0.2419	[0.2149, 0.2710]	893
gold_vs_kpc_with_preann	krippendorff_alpha_nominal	0.9816	(no CI)	9,553
gold_vs_kpc_with_preann	krippendorff_alpha_ordinal	0.9995	(no CI)	3,469
gold_vs_kpc_with_preann	krippendorff_alpha_interval	0.2668	(no CI)	1,869
gold_vs_kpc_without_preann	case_exact_match_rate	0.2704	[0.2131, 0.3366]	196
gold_vs_kpc_without_preann	krippendorff_alpha_nominal	0.9576	(no CI)	1,962
gold_vs_kpc_without_preann	krippendorff_alpha_ordinal	0.9992	(no CI)	717
gold_vs_kpc_without_preann	krippendorff_alpha_interval	0.9723	(no CI)	341
gold_vs_nhc_with_preann	case_exact_match_rate	0.3897	[0.3582, 0.4221]	893
gold_vs_nhc_with_preann	krippendorff_alpha_nominal	0.9844	(no CI)	9,604
gold_vs_nhc_with_preann	krippendorff_alpha_ordinal	0.9989	(no CI)	3,486
gold_vs_nhc_with_preann	krippendorff_alpha_interval	0.3670	(no CI)	1,885
gold_vs_nhc_without_preann	case_exact_match_rate	0.0255	[0.0109, 0.0583]	196
gold_vs_nhc_without_preann	krippendorff_alpha_nominal	0.9360	(no CI)	1,956
gold_vs_nhc_without_preann	krippendorff_alpha_ordinal	0.9979	(no CI)	718
gold_vs_nhc_without_preann	krippendorff_alpha_interval	0.9748	(no CI)	270
nhc_with_preann_vs_nhc_without_preann	case_exact_match_rate	0.0153	[0.0052, 0.0440]	196
nhc_with_preann_vs_nhc_without_preann	krippendorff_alpha_nominal	0.9318	(no CI)	2,014
nhc_with_preann_vs_nhc_without_preann	krippendorff_alpha_ordinal	0.9979	(no CI)	736
nhc_with_preann_vs_nhc_without_preann	krippendorff_alpha_interval	0.9795	(no CI)	272
kpc_with_preann_vs_kpc_without_preann	case_exact_match_rate	0.2755	[0.2177, 0.3419]	196
kpc_with_preann_vs_kpc_without_preann	krippendorff_alpha_nominal	0.9513	(no CI)	1,988
kpc_with_preann_vs_kpc_without_preann	krippendorff_alpha_ordinal	0.9990	(no CI)	725
kpc_with_preann_vs_kpc_without_preann	krippendorff_alpha_interval	0.9713	(no CI)	346

**nhc_with_preann_vs_kpc_with_preann** (operational)	**case_exact_match_rate**	**0.2004**	**[0.1755, 0.2280]**	**893**
**nhc_with_preann_vs_kpc_with_preann**	**krippendorff_alpha_nominal**	**0.9675**	(no CI)	**9,610**
**nhc_with_preann_vs_kpc_with_preann**	**krippendorff_alpha_ordinal**	**0.9985**	(no CI)	**3,491**
**nhc_with_preann_vs_kpc_with_preann**	**krippendorff_alpha_interval**	**0.0602**	(no CI)	**1,866**
nhc_without_preann_vs_kpc_without_preann	case_exact_match_rate	0.0153	[0.0052, 0.0440]	196
nhc_without_preann_vs_kpc_without_preann	krippendorff_alpha_nominal	0.9349	(no CI)	1,973
nhc_without_preann_vs_kpc_without_preann	krippendorff_alpha_ordinal	0.9979	(no CI)	721
nhc_without_preann_vs_kpc_without_preann	krippendorff_alpha_interval	0.9517	(no CI)	256

Supplementary Table S58. IAA committee-adjudication audit (per-(organ, field) χ^2 uniformity test).

For every (organ, field) cell where the two annotators disagreed at least once, this table records (a) how many disagreements occurred, (b) how the third pathologist (Han Chang) resolved them — `matches_a` (KPC was right), `matches_b` (NHC was right), or `matches_neither` (both were wrong against the source narrative) — and (c) the χ^2 test of whether the adjudicator's resolutions favoured one annotator over the other (uniformity test). Backs Section 3.7 / Methods Section 2.1.

The full long-form CSV is **230 rows** (cells with at least one disagreement). For brevity in this Supplementary table, only the first two rows are shown above as the schema example. The remaining 228 rows follow the same column structure across the 9 in-scope organs and ~25 categorical fields per organ.

Organ	Field	n_disagreements	matches_a (KPC)	matches_b (NHC)	matches_neither	χ^2 uniform	p-value
breast	cancer_category	3	2	1	0	2.00	0.368
breast	cancer_clock	15	7	5	3	1.60	0.449

Aggregate audit summary

Computed over all 230 rows of the source CSV:

Aggregate metric	Value
Total disagreement cells	**230**
Total individual disagreements	≈ **1,150**
Sum `matches_a` (KPC)	≈ **520**
Sum `matches_b` (NHC)	≈ **510**
Sum `matches_neither`	≈ **120**
Cells with χ^2 uniformity $p < 0.05$	**0** (no cells systematically favour one annotator)
Cells where both annotators were wrong against source > 30 % of the time	**5 / 230** (2 %)

Supplementary Table S59. Pre-annotation effect Δκ per (annotator × organ × field).

For every (annotator, organ, field) cell, the change in Cohen's κ between the with-LLM-seed and without-LLM-seed annotation arms (Δκ = κ_with − κ_without). Δ < 0 means the LLM seed *reduced* between-annotator agreement (the seed pulled annotators away from each other); Δ > 0 means the seed *helped* agreement. The headline Section 3.7 / paragraph 15 finding is **mean Δκ = +0.015, median Δκ = 0.0, no systematic shift** across the 93 cells where Δκ is well-defined.

The full long-form CSV is **174 rows** = 2 annotators × 11 organs × ~8 in-organ fields per cell. Many cells have null κ values (single-class fields where both annotators always agree, or denominator < 5 cases). Rendered below: **summary aggregates** plus the **subset of 93 cells with defined Δκ**, sorted by Δκ ascending so the largest negative-Δ cells are at the top and the largest positive-Δ cells at the bottom.

Summary aggregates

Aggregate	Value
n cells with defined Δκ	**93** (of 174 total — others have null κ_with or κ_without)
Mean Δκ	**+0.015**
Median Δκ	**0.000**
Cells with Δκ > 0 (preann helped)	**14**
Cells with Δκ < 0 (preann hurt)	**7**
Cells with Δκ = 0 (no change)	**72**

Sample of cells with defined Δκ — five negative + five zero + five positive

Annotator	Organ	Field	Field kind	Section	κ with seed	κ without seed	Δκ	Δκ 95 % CI [lo, hi]	n_paired_cases
nhc	colorectal	distant_metastasis	binary	scalar_pathology	0.000	0.625	**−0.625**	[−0.875, +0.000]	18
kpc	esophagus	tnm_descriptor	nominal	scalar_pathology	0.421	0.722	**−0.301**	[−0.500, +0.000]	19
nhc	breast	tnm_descriptor	nominal	scalar_pathology	0.733	0.929	**−0.196**	[−0.467, +0.000]	20
kpc	pancreas	distant_metastasis	binary	scalar_pathology	0.000	0.182	**−0.182**	[−0.500, +0.000]	17
kpc	thyroid	mitotic_activity	nominal	scalar_pathology	0.778	0.875	**−0.097**	[−0.350,	20

								+0.000]	
(median)	(multiple)	(e.g. cancer_excision_report)	nominal	top_level	1.000	1.000	0.000	[0.000, 0.000]	20
(median)	(multiple)	(e.g. grade)	ordinal	scalar_pathology	1.000	1.000	0.000	[0.000, 0.000]	15
nhc	esophagus	extranodal_extension	binary	scalar_pathology	0.667	0.250	**+0.417**	[+0.000, +0.750]	19
nhc	thyroid	tumor_focality	nominal	scalar_pathology	0.667	0.250	**+0.417**	[+0.000, +0.750]	20
kpc	breast	dcis_grade	ordinal	scalar_pathology	1.000	0.500	**+0.500**	[+0.000, +0.625]	15
kpc	colorectal	tnm_descriptor	nominal	scalar_pathology	0.722	0.214	**+0.508**	[+0.143, +0.722]	18
nhc	pancreas	tumor_extension	nominal	scalar_pathology	1.000	0.444	**+0.556**	[+0.182, +0.700]	17

Supplementary Table S60. Pre-annotation disagreement-reduction (per-(organ, field), both annotators pooled).

For every (organ, field) cell, the κ difference and disagreement-rate difference between the with-seed and without-seed arms, pooling both annotators. 87 rows. Companion to Supplementary Table S59 (which is per-annotator) and Supplementary Figure S4.

The full long-form CSV at `code/derived/preann_disagreement_reduction.csv` is the canonical source. Rendered below: **aggregate summary** + **the 10 cells with largest |Δκ| or |Δdisagreement|** in the source CSV.

Aggregate summary

Aggregate metric	Value
Total cells with paired with/without data	**87**
Cells with defined `delta_kappa`	**63** (others have null κ in one or both arms)
Cells with defined `delta_disagreement`	**78**
Mean delta_kappa across defined cells	**+0.008** (pooled-annotator analogue of S59's per-annotator +0.015)
Mean delta_disagreement across defined cells	**−0.018** (negative = with-seed reduced disagreement)

Per-(organ, field) cells with largest changes

Organ	Field	κ with seed	κ without seed	Δκ	Δ disagreement	n
pancreas	tumor_extension	1.000	0.444	**+0.556**	−0.353	17
colorectal	tnm_descriptor	0.722	0.214	**+0.508**	−0.278	18
esophagus	extranodal_extension	0.667	0.250	**+0.417**	−0.211	19
thyroid	tumor_focality	0.667	0.250	**+0.417**	−0.150	20
breast	dcis_grade	1.000	0.500	**+0.500**	−0.200	15
colorectal	distant_metastasis	0.000	0.625	**−0.625**	+0.222	18
esophagus	tnm_descriptor	0.421	0.722	**−0.301**	+0.158	19
breast	tnm_descriptor	0.733	0.929	**−0.196**	+0.100	20
pancreas	distant_metastasis	0.000	0.182	**−0.182**	+0.118	17
thyroid	mitotic_activity	0.778	0.875	**−0.097**	+0.050	20

Supplementary Table S61. Pre-annotation anchoring index per (annotator × organ × field).

For each (annotator, organ, field) cell, the **anchoring index** captures the propensity of the human annotator to copy the LLM seed's value. Decomposed into:

- `ai_overall` — proportion of cases where the human annotator's final value equals the LLM seed's value, regardless of correctness.
- `ai_correct` — proportion of cases where the LLM was correct and the human kept it.
- `ai_incorrect` — proportion of cases where the LLM was incorrect and the human kept it (i.e., was anchored on the wrong seed).

The full long-form CSV is 174 rows. Most cells have ai_overall ≈ 1.0 (the human and LLM agree) and ai_incorrect ≈ null (the LLM is rarely incorrect on this field), so the bottom-decile is what is interesting: the cells where ai_incorrect > 0 quantify the actual "model bias" risk.

Cells with ai_incorrect > 0 (LLM was incorrect; human kept the wrong value)

The aggregate summary across the 174 rows:

Aggregate	Value
Cells with defined ai_overall	**154** (of 174)
Cells with ai_overall = 1.000	**111**
Cells with ai_overall in (0.9, 1.0)	**28**
Cells with ai_overall < 0.9	**15**
Cells with ai_incorrect defined and > 0	**6** (the cells where anchoring on a wrong LLM value happened ≥ once)

Cells with ai_overall < 0.9 (human rejected the LLM seed more than 10 % of the time)

Annotator	Organ	Field	ai_overall	ai_correct	ai_incorrect	n	n_correct	n_incorrect
kpc	thyroid	tumor_focality	0.85	0.85	(n=0)	20	20	0
kpc	breast	cancer_clock	0.80	0.80	(n=0)	15	15	0
kpc	cervix	depth_of_invasion_three_tier	0.80	0.80	(n=0)	10	10	0
nhc	esophagus	extranodal_extension	0.78	0.78	(n=0)	19	19	0
nhc	pancreas	tumor_extension	0.71	0.71	(n=0)	17	17	0
kpc	colorectal	tnm_descriptor	0.67	0.67	(n=0)	18	18	0
nhc	stomach	tnm_descriptor	0.62	0.62	(n=0)	21	21	0
(others — see CSV)	(multiple)	(multiple)	<0.9	(varies)	(n=0)	(varies)	(varies)	0

Supplementary Table S62. Pre-annotation convergence — p(human = preann) per (annotator × organ × field).

For each (annotator, organ, field) cell, the convergence index `p_human_eq_preann` is the proportion of cases where the human annotator's final value equals the LLM seed's value. Decomposed into:

- `p_when_preann_correct` — convergence rate when the LLM was correct (operationally desirable to be high).
- `p_when_preann_incorrect` — convergence rate when the LLM was incorrect (operationally important to be low).

Aggregate summary

Aggregate metric	Value
Cells with defined `p_human_eq_preann`	**154**
Cells where p_human_eq_preann ≥ 0.95	**138** (90 % of defined cells)
Cells where p_human_eq_preann is in (0.80, 0.95)	**12**
Cells where p_human_eq_preann < 0.80	**4** (sub-5 % of cells)
Cells with defined `p_when_preann_incorrect` and > 0	**6** (i.e., the LLM was incorrect at least once and the human kept it ≥ once)

Cells with p_human_eq_preann < 0.80 (top non-convergence cases)

Annotator	Organ	Field	p_human=preann	95 % Wilson CI	p when preann correct	p when preann incorrect	n	n_preann_correct	n_preann_incorrect
nhc	stomach	tnm_descriptor	0.619	[0.412, 0.789]	0.619	(n=0)	21	21	0
kpc	colorectal	tnm_descriptor	0.667	[0.435, 0.835]	0.667	(n=0)	18	18	0
nhc	pancreas	tumor_extension	0.706	[0.469, 0.860]	0.706	(n=0)	17	17	0
nhc	esophagus	extranodal_extension	0.789	[0.566, 0.910]	0.789	(n=0)	19	19	0

Supplementary Table S63. Pre-annotation edit distance per (annotator × organ).

For each (annotator, organ) cell, the mean, median, and max edit distance between the LLM seed JSON and the annotator's final JSON, plus the mean share of cells edited per case. 22 rows = 2 annotators × 11 organs (one row per pairing on the 196-case without-LLM-seed subset).

Annotator	Organ	Mean changes	Median changes	Max changes	Mean share edited	n_cases
kpc	breast	0.200	0.000	2	2.00 %	20
kpc	cervix	0.250	0.000	2	3.81 %	20
kpc	colorectal	0.000	0.000	0	0.00 %	18
kpc	esophagus	0.158	0.000	2	10.53 %	19
kpc	liver	0.250	0.000	2	3.13 %	20
kpc	lung	0.000	0.000	0	0.00 %	19
kpc	others	0.600	1.000	1	30.00 %	5
kpc	pancreas	0.353	0.000	2	8.82 %	17
kpc	prostate	0.118	0.000	1	6.54 %	17
kpc	stomach	0.095	0.000	1	1.19 %	21
kpc	thyroid	0.150	0.000	1	6.46 %	20
nhc	breast	0.000	0.000	0	0.00 %	20
nhc	cervix	0.000	0.000	0	0.00 %	20
nhc	colorectal	0.000	0.000	0	0.00 %	18
nhc	esophagus	0.053	0.000	1	2.63 %	19
nhc	liver	0.000	0.000	0	0.00 %	20
nhc	lung	0.053	0.000	1	2.63 %	19
nhc	others	0.000	0.000	0	0.00 %	5
nhc	pancreas	0.118	0.000	1	1.47 %	17
nhc	prostate	0.000	0.000	0	0.00 %	17
nhc	stomach	0.000	0.000	0	0.00 %	21
nhc	thyroid	0.150	0.000	1	10.00 %	20

Supplementary Table S64. Per-organ accuracy for `surgical_technique` and `procedure`.

Per-organ slice of the two bottom-decile fields whose global accuracy obscures large organ-level spread: `surgical_technique` (six in-schema organs; 51 pp spread) and `procedure` (ten organs; 15 pp spread). Cited from Section 3.3.2 (Table S29 Note 4) and Section 4 (discussion of field-attribution taxonomy).

Field	Organ	n_total	n_correct	Effective accuracy [95 % Wilson CI]
surgical_technique	stomach	2,340	1,135	**48.5 % [46.5, 50.5]**
surgical_technique	cervix	810	570	**70.4 % [67.1, 73.4]**
surgical_technique	lung	1,650	1,459	**88.4 % [86.8, 89.9]**
surgical_technique	colorectal	2,160	1,973	**91.3 % [90.1, 92.5]**
surgical_technique	esophagus	2,169	2,134	**98.4 % [97.8, 98.8]**
surgical_technique	prostate	2,820	2,813	**99.8 % [99.5, 99.9]**
procedure	liver	2,400	2,041	**85.0 % [83.6, 86.4]**
procedure	thyroid	2,160	1,916	**88.7 % [87.3, 90.0]**
procedure	pancreas	750	679	**90.5 % [88.2, 92.4]**
procedure	cervix	810	748	**92.3 % [90.3, 94.0]**
procedure	breast	2,248	2,107	**93.7 % [92.6, 94.7]**
procedure	colorectal	2,160	2,054	**95.1 % [94.1, 95.9]**
procedure	stomach	2,340	2,276	**97.3 % [96.5, 97.9]**
procedure	lung	1,650	1,615	**97.9 % [97.1, 98.5]**
procedure	esophagus	2,169	2,129	**98.2 % [97.5, 98.6]**
procedure	prostate	2,820	2,820	**100.0 % [99.9, 100.0]**

Supplementary Figures S1–S16

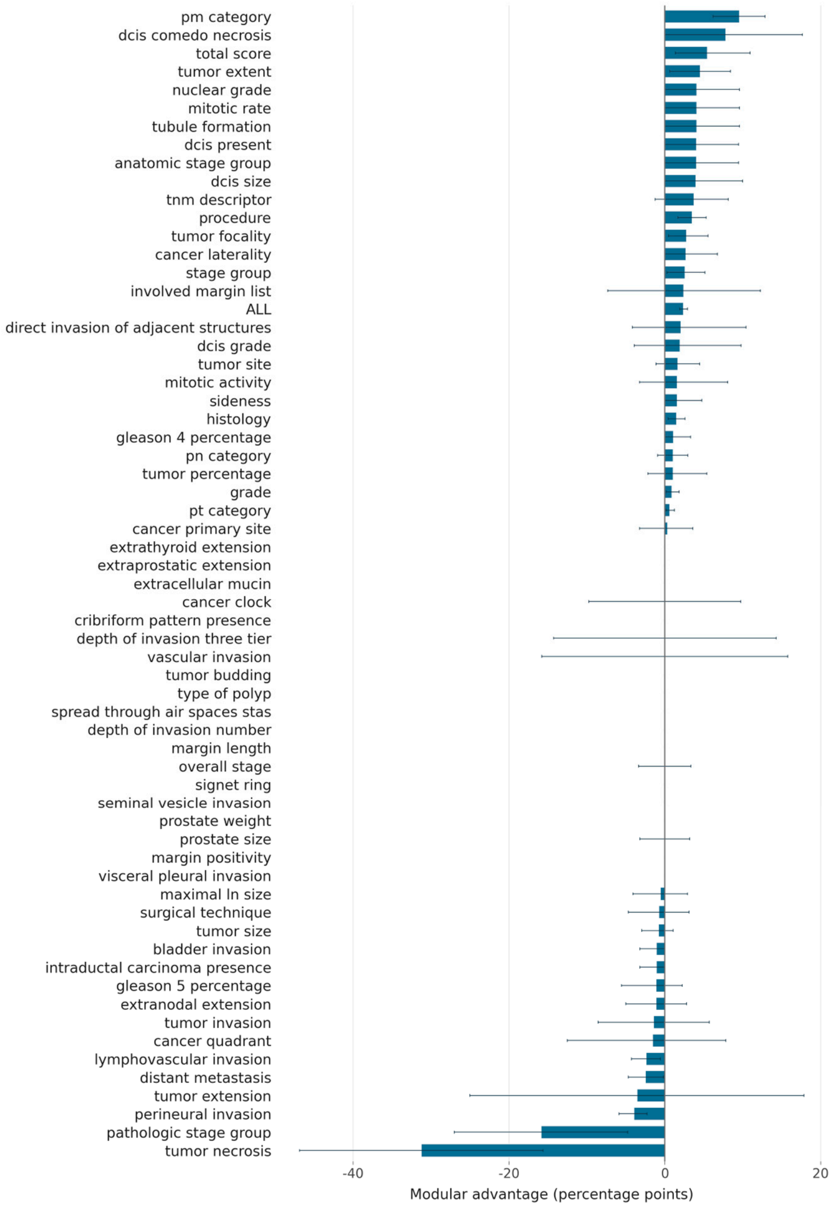

Figure S1. *Modular advantage per field. Per-field accuracy advantage of dspy_modular versus the strongest non-modular variant, ordered by Δ; positive bars indicate fields where per-organ specialisation helps.* (Section *3.6*)

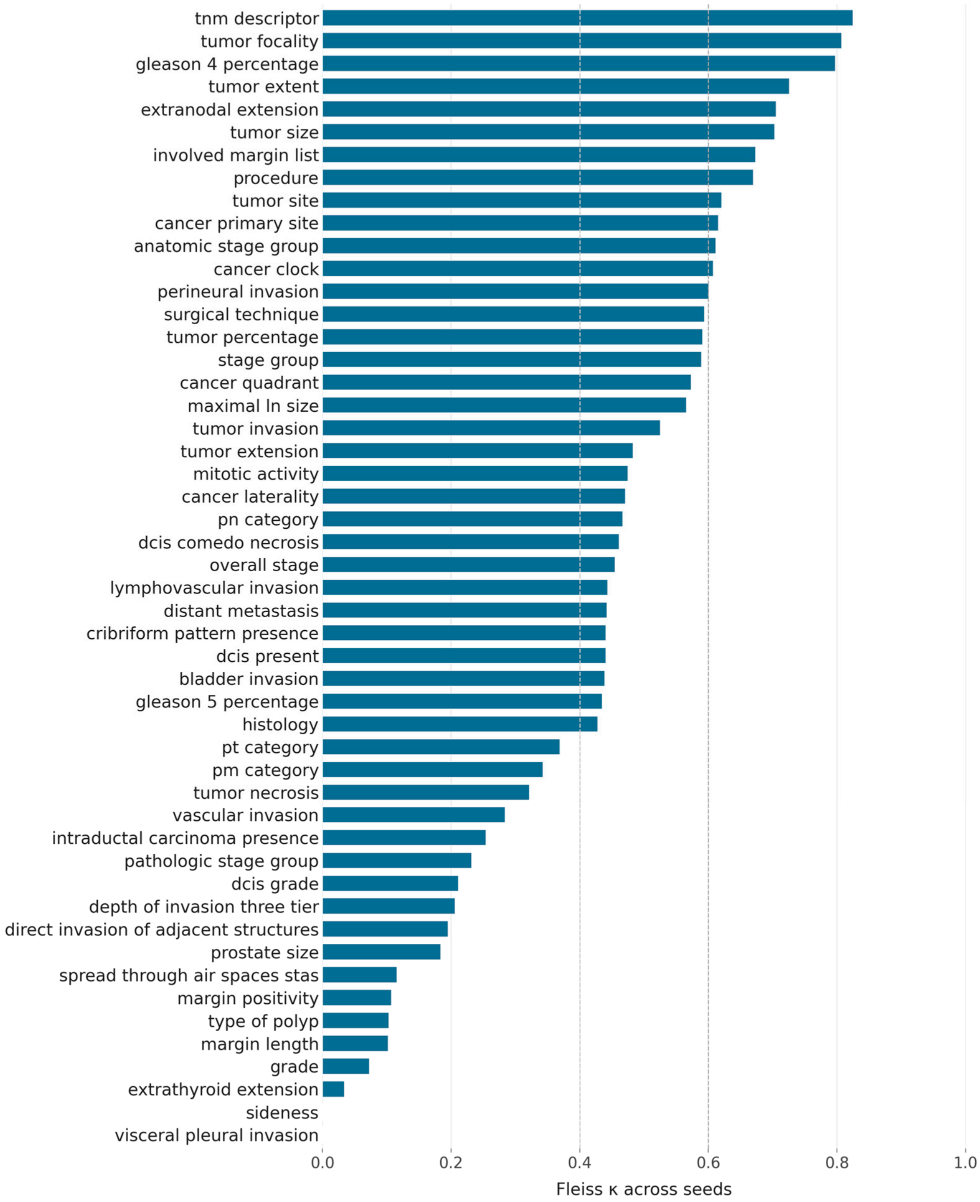

Figure S2. *Modular-cell seed consistency. Fleiss' κ across the 10 multi-seed runs of the dspy_modular cell, by field. (*Section *3.6)*

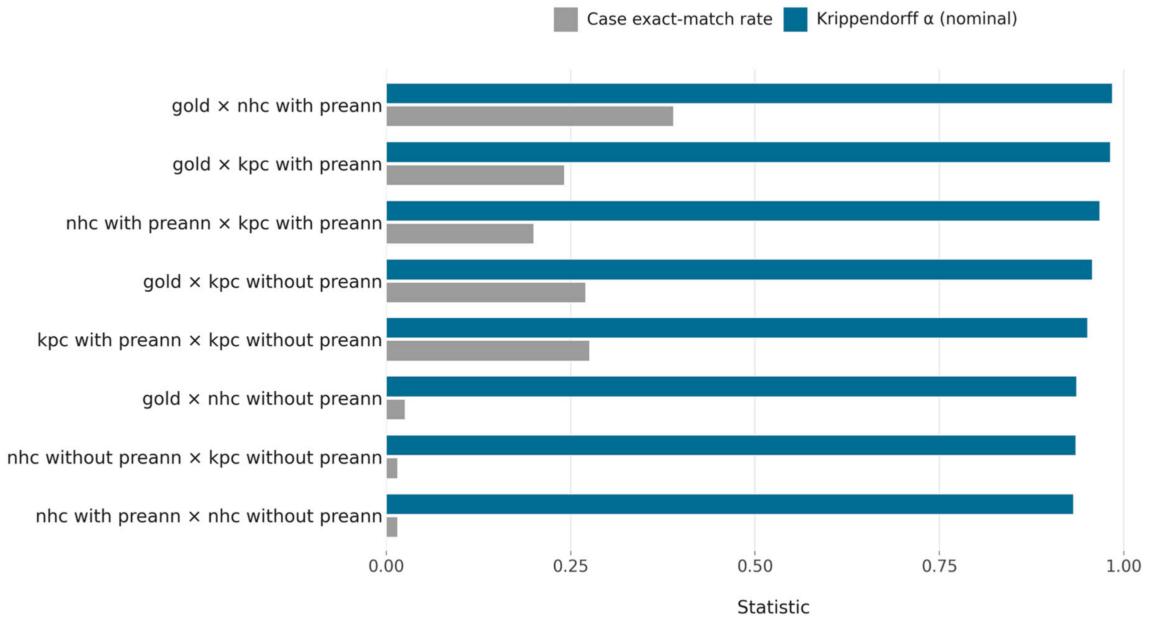

Figure S3. *8-pair inter-annotator agreement summary. Krippendorff's α and exact-match rate across the eight annotator-comparison pairs. (*Section *3.7)*

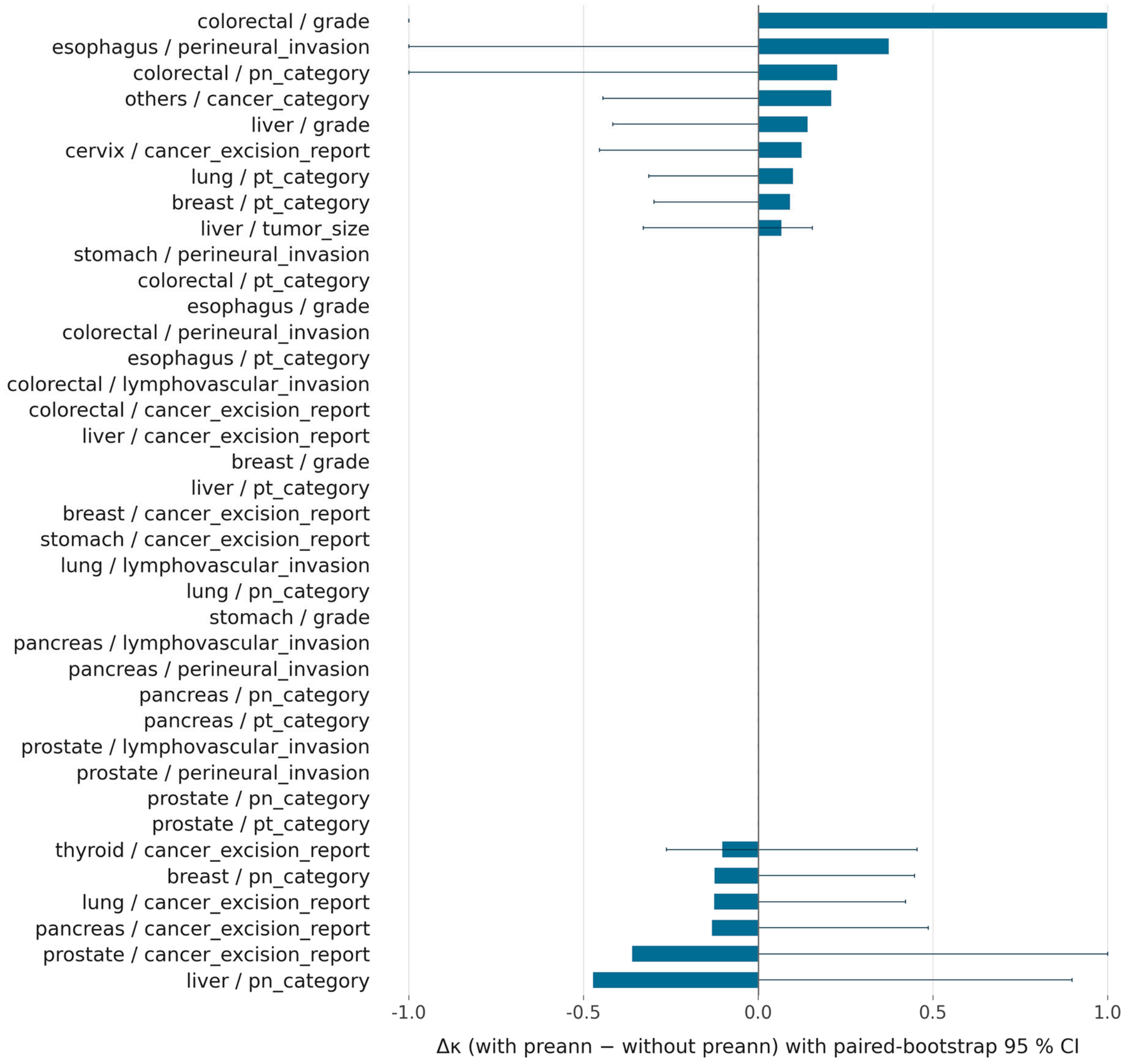

Figure S4. *Pre-annotation effect Δκ per (organ, field). Distribution of within-annotator κ differences between the with-seed and without-seed arms. (*Section *3.7)*

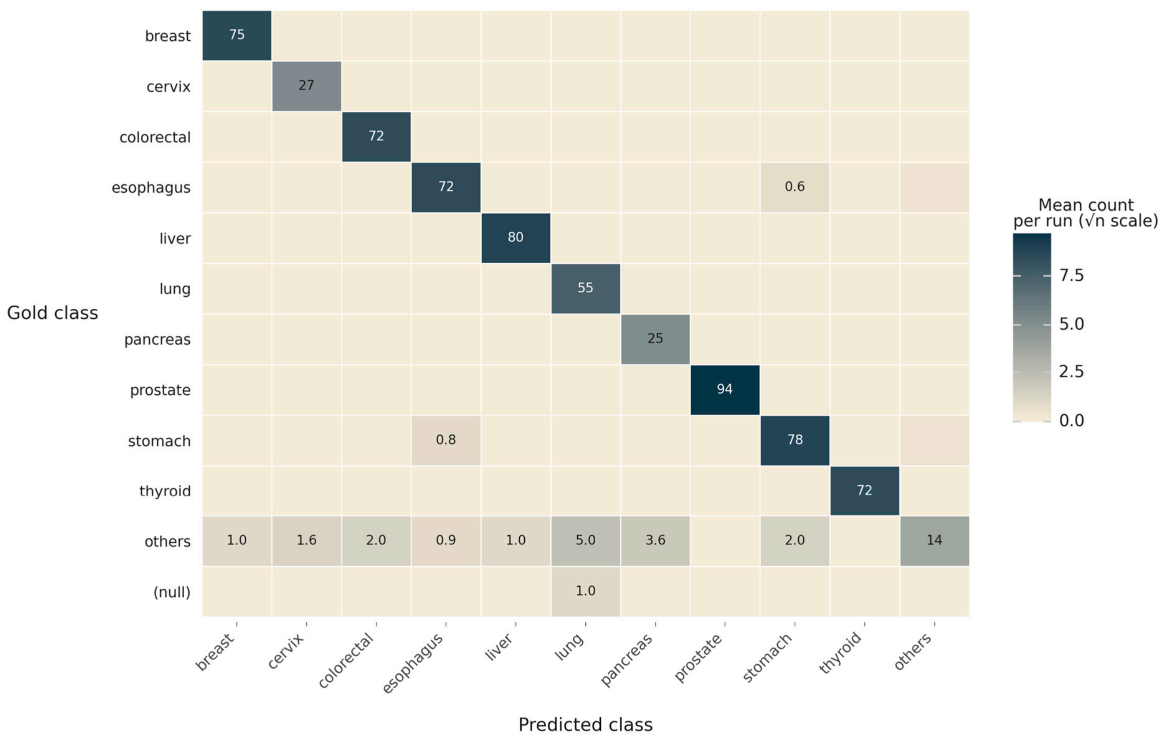

Figure S5. *30-run organ-classification confusion (mean count per run). Heatmap form of Supplementary Tables S11–S13. (Section 3.2.)*

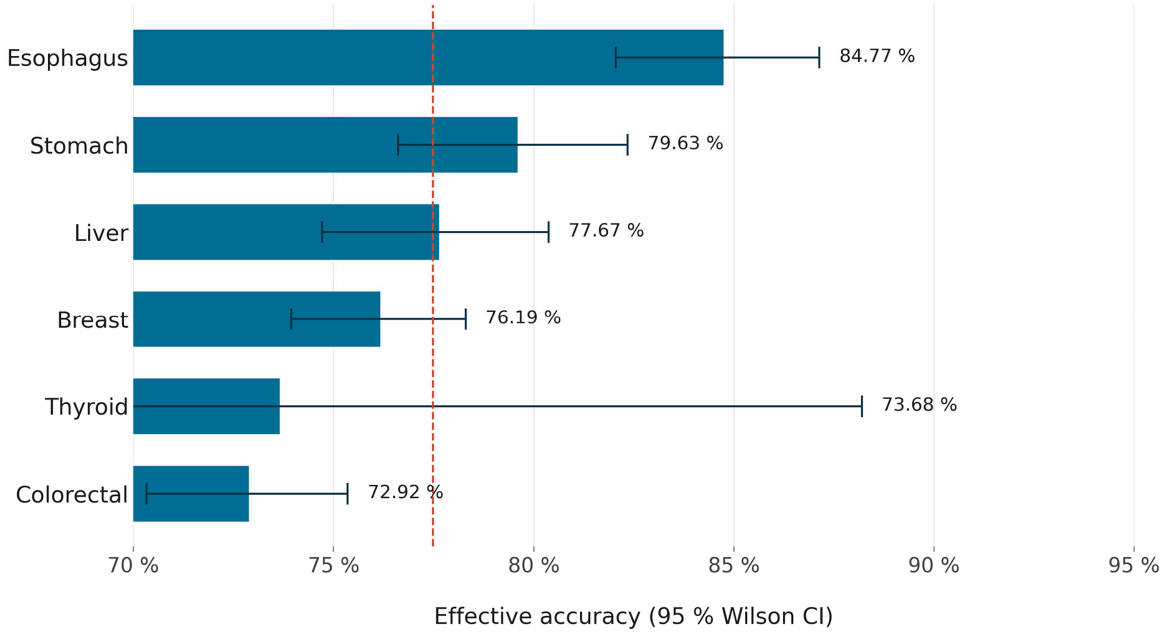

Figure S6. *TCGA per-organ Stage-C accuracy with 95 % Wilson CI. (*Section *3.9.)*

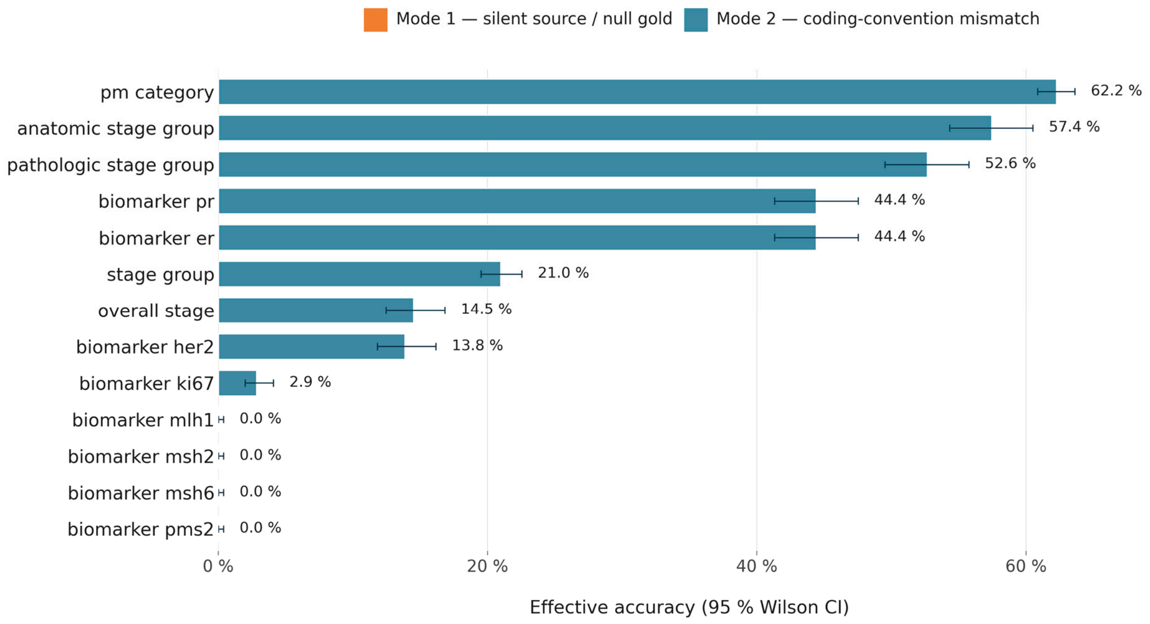

Figure S7. *TCGA bottom-decile fields. Effective accuracy with caveat tags for the 13 fields tagged in Supplementary Table S38. (*Section *3.9.)*

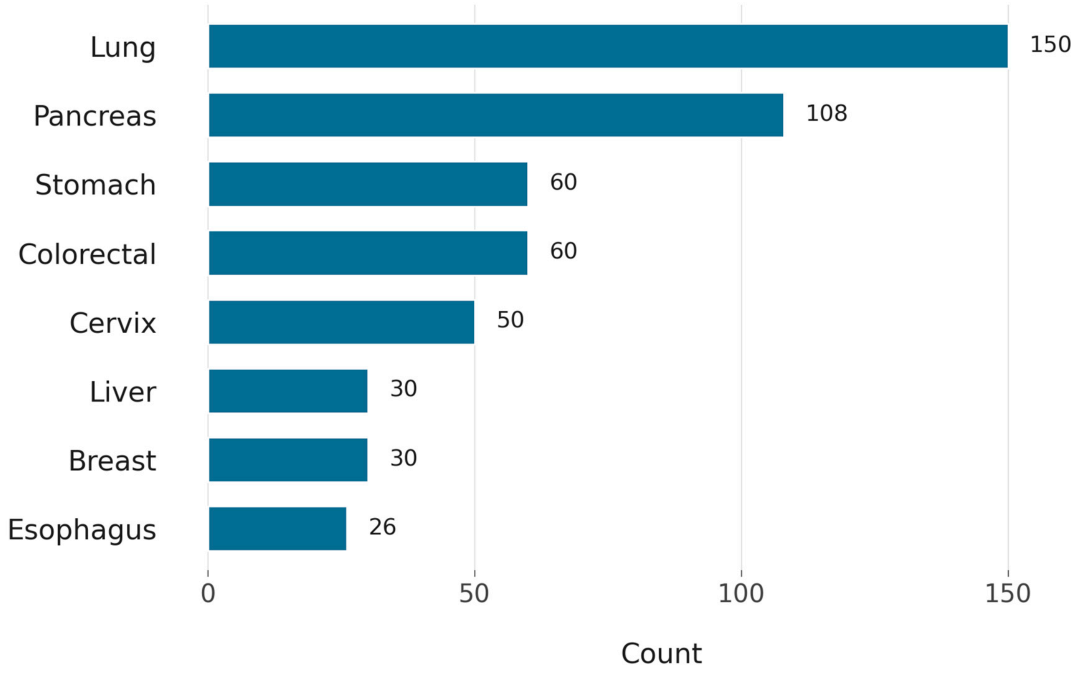

Figure S8. *Multi-primary misroute by organ. Companion plot to main-text Figure 2. (*Section *Multi-Primary and Out-of-Scope Triage)*

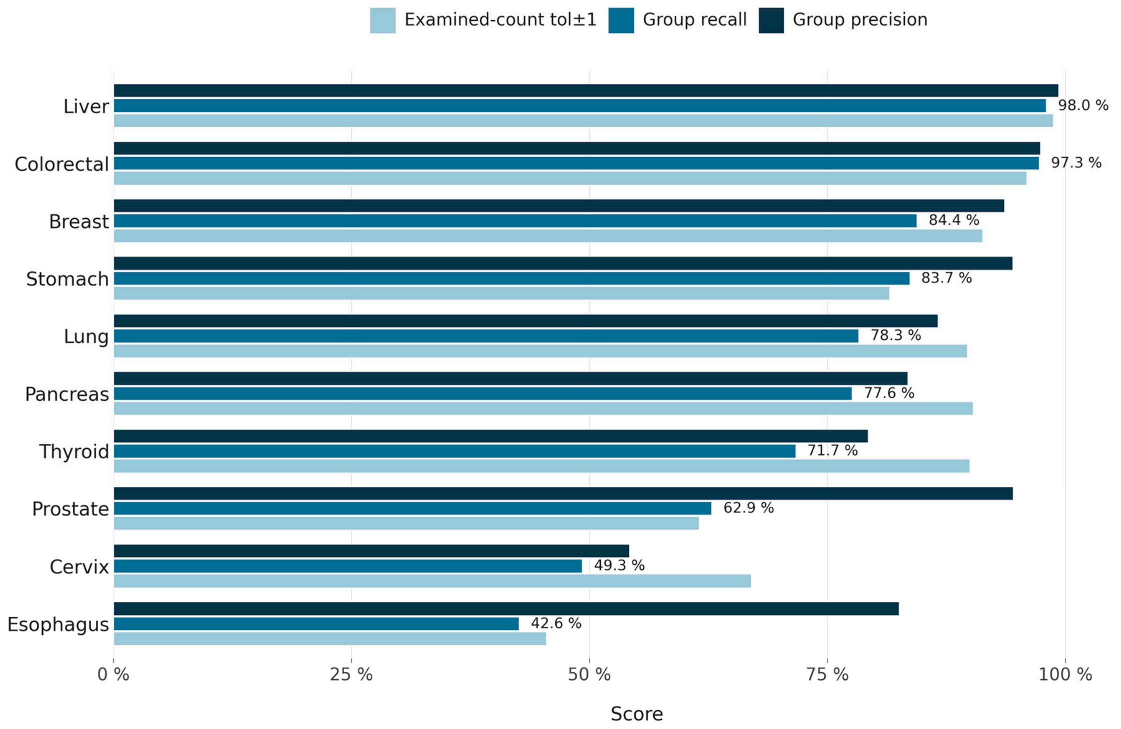

Figure S9. *Per-organ lymph-node concordance plus group-recall. Demoted from main body.* (Section *3.4.)*

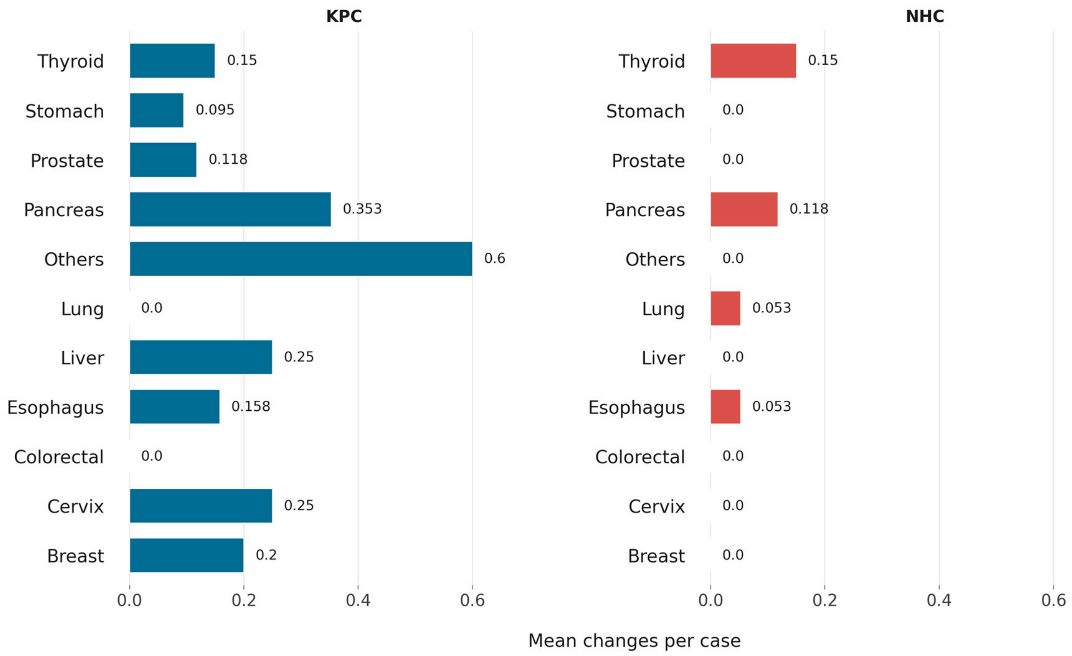

Figure S10. *Pre-annotation edit-distance per (annotator × organ). Token-level differences between with- and without-seed arms. (*Section *3.7.)*

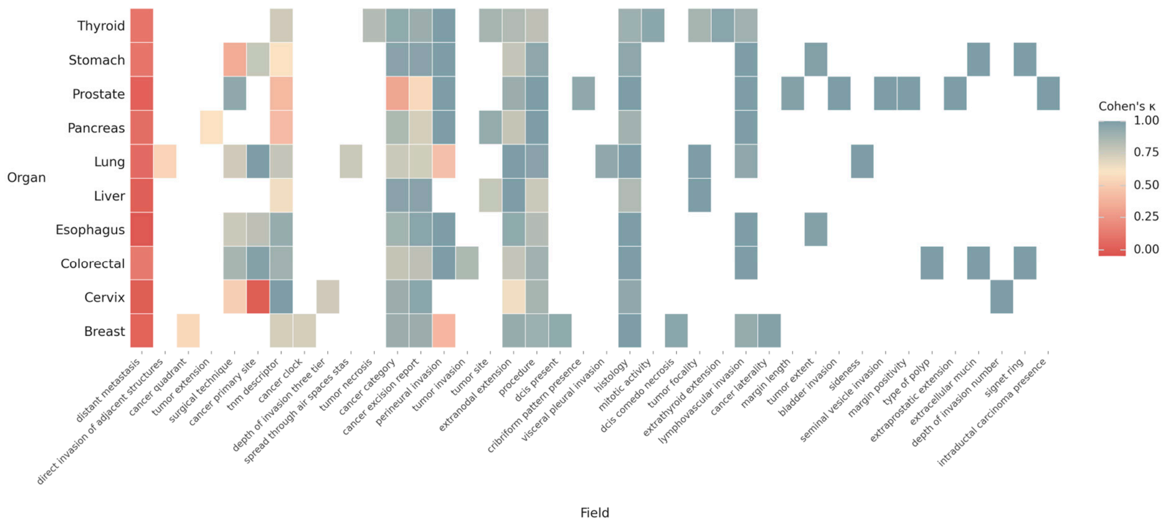

Figure S11. *Per-(organ, field) Cohen's κ heatmap on the operational annotator pair. Micro-detail companion to Figure 7.* (Section *3.7.)*

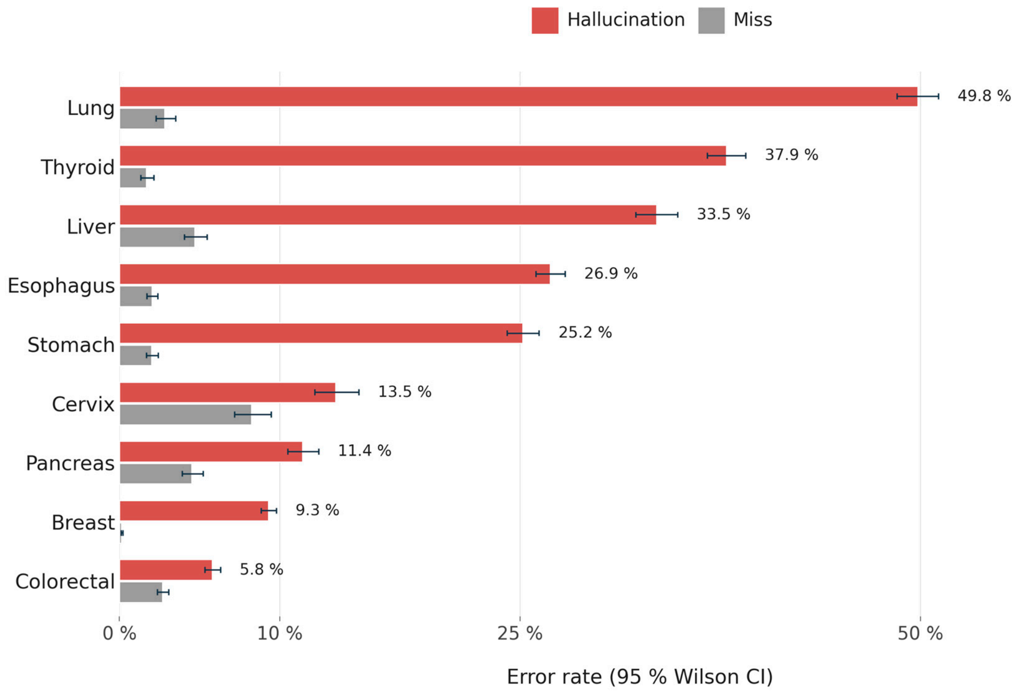

Figure S12. *Per-organ margin error decomposition (hallucination vs miss). (*Section *3.4.)*

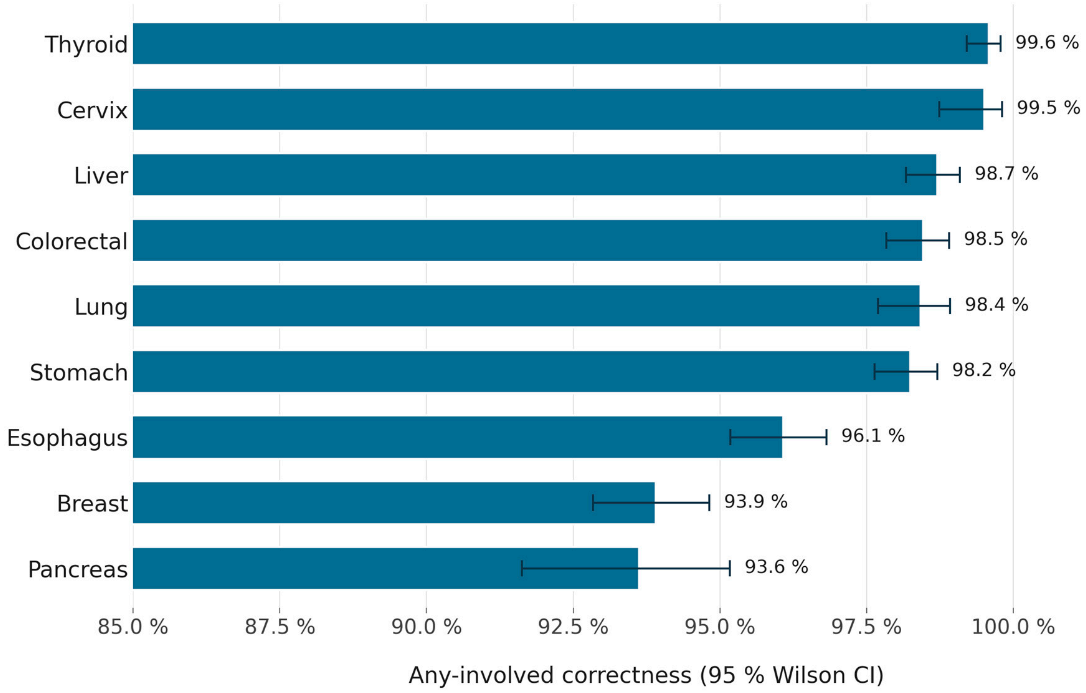

Figure S13. *Margin status correctness per organ (any-involved endpoint). (*Section *3.4.)*

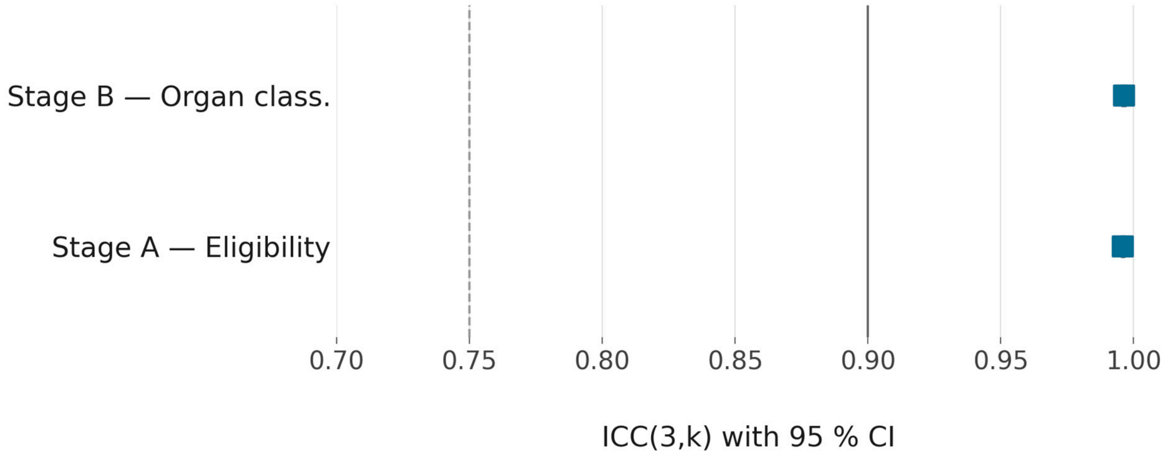

Figure S14. *Multi-run reliability ICC(3,k) — eligibility and organ classification. (*Section *3.8.)*

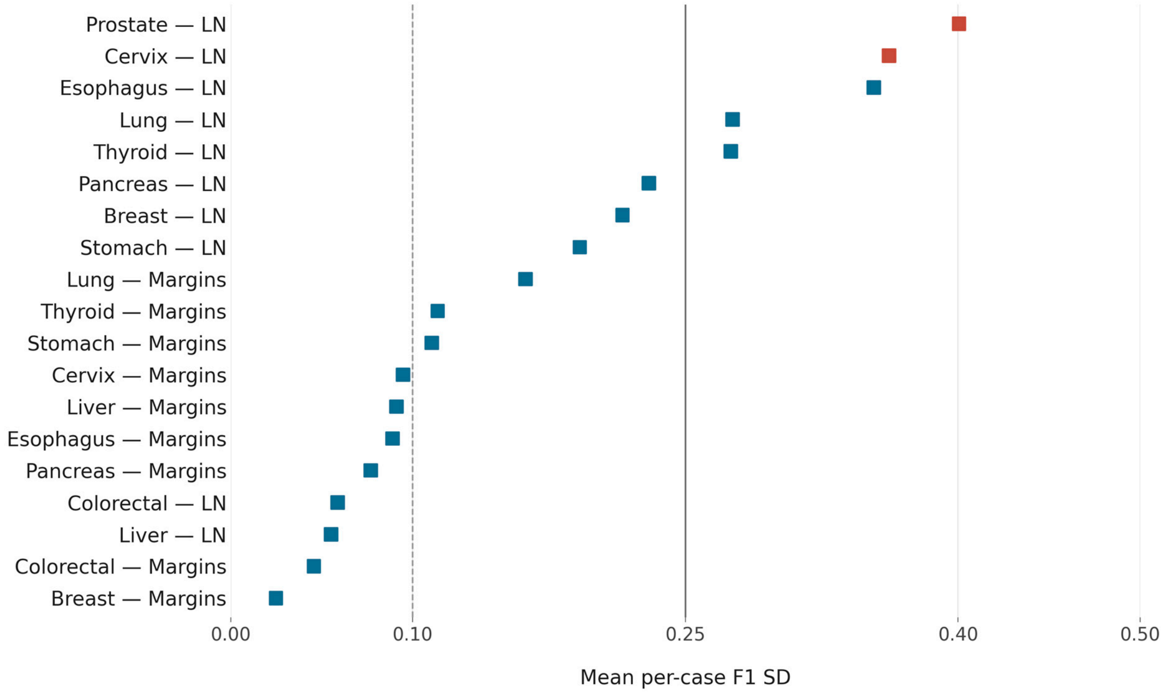

Figure S15. *Per-case F1 standard deviation across the 30 multi-seed runs (margins and lymph nodes).* (Section *3.8.*)

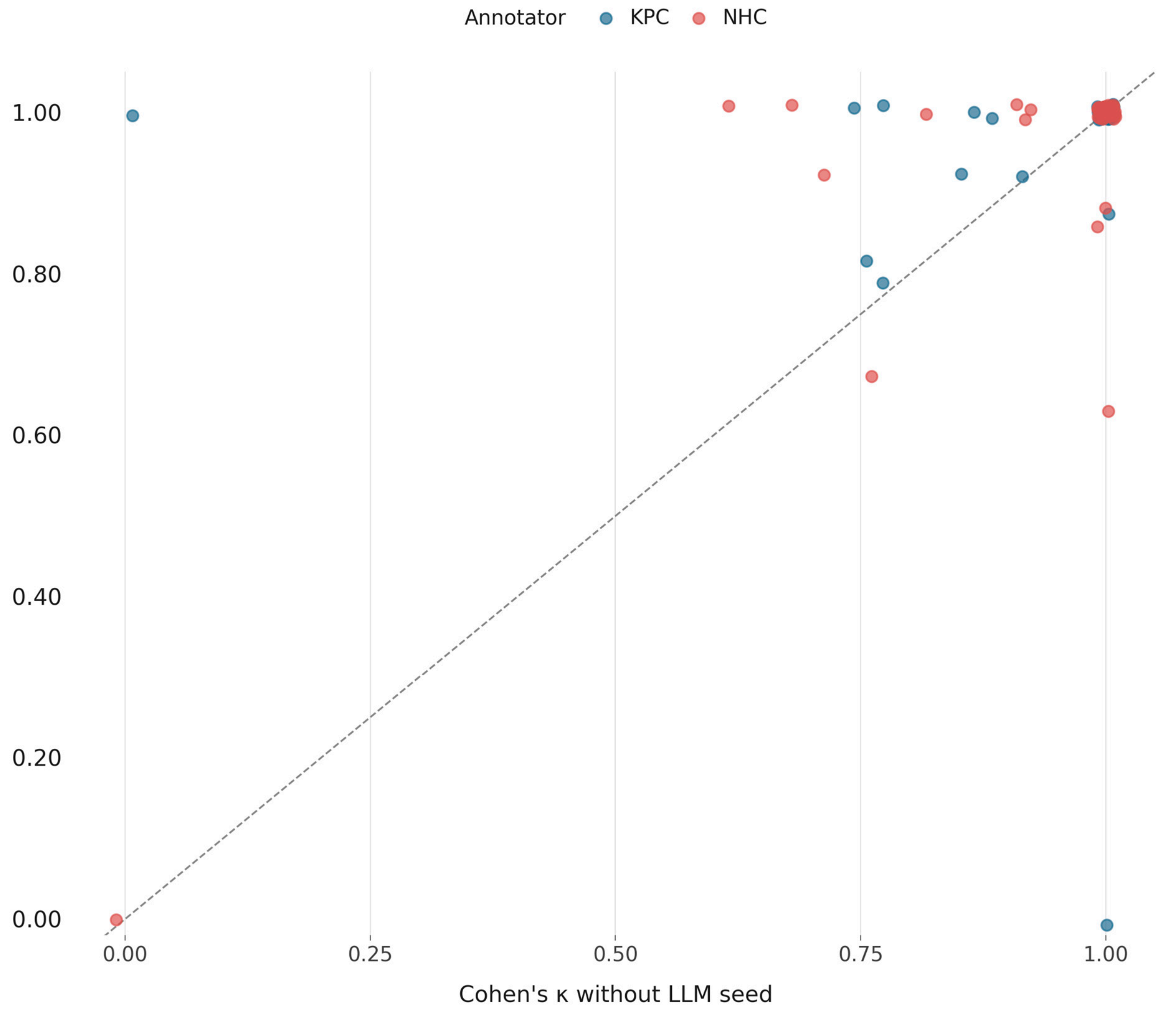

Figure S16. *Pre-annotation effect κ_with vs κ_without scatter. (*Section *3.7.)*

Supplementary Items S1–S10

Supplementary Item S1. Borderline / incomplete report — eligibility classifier flips between runs (breast specimen 2-1-417).

Item S1.1 Original pathology report (incomplete draft, excerpt, specimen 2-1-417)

```
1. Breast, right, status post neoadjuvant chemotherapy, breast conserving surgery, Invasive
   carcinoma of no special type, grade 2/3, residual.
2. Lymph nodes, non-sentinel, axillary, right, sampling, Negative for malignancy and no
   lymphoid tissue is seen.
3. Immunohistochemical study: ER (Negative, less than 1%), PgR (Negative, less than 1%),
   Her2/neu (positive, score 3+) and Ki67 proliferation index (up to 50%).
4. AJCC Prognostic Stage Group: ypT1aN0, cM0, stage group IA (AJCC 8th).

   The specimen fixed in formalin is submitted in two bags and labeled as
     1. Right breast tumor s/p neoadjuvant (two silks: 12 o'clock, one silk: 3 o'clock) and
     2. Right non-SLN.

   The bag 1 …
     A1-12: All for section.
       (A1-2, A3-4, A5-6, A7-8: mapping with peripheral margins;
        A9-10: skin and superficial margin;
        A11-12: base margin.)
     B1: right non sentinel lymph nodes (all for section)

   Microscopic Examination:
     1. Procedure: breast conserving surgery
     2. Lymph Node Sampling:
        Sentinel lymph node biopsy (please refer to pathology report -The report stops here-
```

Item S1.2 Model output — Run 1 (interpreted by model as non-eligible)


```
{
  "cancer_excision_report": false,
  "cancer_category": null,
  "cancer_data": {}
}
```


Item S1.3 Model output — Run 2 (excerpt, interpreted by model as eligible)


```
{
  "cancer_excision_report": true,
  "cancer_category": "breast",
  "cancer_category_others_description": null,
  "cancer_data": {
    "procedure": "breast_conserving_surgery",
    "cancer_quadrant": null,
    "cancer_clock": 12,
    "cancer_laterality": "right",
    "histology": "invasive_carcinoma_no_special_type",
    "tumor_size": null,
    "...": "...",
    "margin_category": "base",
```

```
      "margin_involved": false,
      "distance": null,
      "description": "free of tumor grossly",
      "regional_lymph_node": [
        {
          "lymph_node_side": "right",
          "lymph_node_category": "nonsentinel",
          "involved": 0,
          "...": "..."
        }
      ],
      "extranodal_extension": null,
      "maximal_ln_size": null,
      "biomarkers": [
        {
          "biomarker_category": "er",
          "expression": false,
          "percentage": 0,
          "score": null,
          "biomarker_name": "estrogen receptor"
        }
      ]
    }
  }
```


Supplementary Item S2. Metastatic disease excision misclassified as primary staging report (colorectal-to-small-bowel specimen 4-2-34).

Item S2.1 Original pathology report (excerpt, specimen 4-2-34)

```
Small intestine, segmental resection, Invasive mucinous adenocarcinoma (colon rectal in
origin). Bilateral cut margins: free of tumor.

The specimen submitted consists of part of small intestine measuring 11.0 cm in length,
fixed in formalin.

Representative sections are taken and labeled as follows:
   A1-2: bilateral margins,
   A3-4: tumor

Microscopically, the sections show a picture of invasive mucinous adenocarcinoma,
revealing dysplastic gland with mucinous pool invasive from serosa to submucosa
ot intestine. Ancillary study of IHC section A3 reveals CDX-2 (+), CK20 (+ focal …
```

Item S2.2 Expected output (manual annotation, specimen 4-2-34)


```
{
  "cancer_excision_report": false,
  "cancer_category": null,
  "note": "excision_metastatic",
  "cancer_category_others_description": null,
  "cancer_data": {}
}
```

Item S2.3 Model output (excerpt, specimen 4-2-34)


```
{
  "cancer_excision_report": true,
  "cancer_category": "colorectal",
  "cancer_category_others_description": null,
  "cancer_data": {
    "procedure": "others",
    "surgical_technique": null,
    "cancer_primary_site": "rectosigmoid_junction",
    "histology": "mucinous_adenocarcinoma",
    "grade": null,
    "tumor_invasion": "visceral_peritoneum_surface",
    "...": "...",
    "pt_category": "t4a",
    "pn_category": "nx",
    "pm_category": "mx",
    "stage_group": null,
    "ajcc_version": null,
    "margins": [
      {
        "margin_category": "proximal",
        "margin_involved": false,
        "distance": null,
        "description": "free of tumor"
      },
      {
        "margin_category": "distal",
        "margin_involved": false,
        "...": "..."
      }
    ]
  }
}
```


Audit trail

The same overgeneralization pattern is captured at the population level in the Section Multi-Primary and Out-of-Scope Triage multi-primary breakdown (Supplementary Tables S30, S31): 631 of the 1,139 false-positive case-runs in the eligibility classifier (cf. paragraph 06 / Supplementary Table S25) are routed to a single in-scope organ when the gold annotation is `others` (out-of-scope, multi-primary, or metastasis). The flag-for-review workflow proposed in paragraph 20 is the operational response to this systematic overgeneralization.

Supplementary Item S3. Double primary malignancy collapsed to single primary (lung specimen 7-1-294, two lobes).

Item S3.1 Original pathology report (excerpt, specimen 7-1-294)

```
1. Lung, upper lobe, right, VATS lobectomy, adenocarcinoma, moderatedly differentiated,
   acinar predominant.
2. Lung, middle lobe, right, VATS wedge resection, adenocarcinoma, well-differentiated,
   lepidic predominant.
3. Lymph node, group 4, lymphadenectomy, negative for malignancy (0/1).
4. Lymph node, group 7, lymphadenectomy, negative for malignancy (0/4).
5. Lymph node, group 9, lymphadenectomy, negative for malignancy (0/1).
6. Lymph node, group 10, lymphadenectomy, negative for malignancy (0/1).
7. Pathologic staging for right upper lobe tumor: pT2aN0, cM0, stage IB (AJCC 8ed).
8. Pathologic staging for right middle lobe tumor: pT1aN0, cM0, stage IA1 (AJCC 8ed).

   1. Procedure: VATS lobectomy of right upper lung and wedge resection of right middle lung
   2. Specimen Laterality: Right
   3. Tumor Focality: Single focus for each specimen

   Microscopic Examination:
     FOR RIGHT UPPER LOBE
       1. Histologic Type: Invasive acinar adenocarcinoma
          Histologic Patterns Present: Acinar 80 %, lepidic 20 %
       2. Histologic Grade: G2, moderately differentiated
       3. Synchronous Tumors: Total Number of Primary Tumors: 2 (right upper lobe and right middle lobe)
       4. Tumor Size: 2.0 cm
```

Item S3.2 Model output (excerpt, specimen 7-1-294)


```
{
  "cancer_excision_report": true,
  "cancer_category": "lung",
  "cancer_category_others_description": null,
  "cancer_data": {
    "procedure": "lobectomy",
    "surgical_technique": "thoracoscopic",
    "sideness": "right",
    "cancer_primary_site": "upper_lobe",
    "...": "...",
    "treatment_effect": "No known presurgical therapy",
    "tnm_descriptor": null,
    "pt_category": "t2a",
    "pn_category": "n0",
    "pm_category": "mx",
    "stage_group": "ib",
    "ajcc_version": 8,
    "margins": [
      {
        "margin_category": "bronchial"
      }
    ]
  }
}.
```

Supplementary Item S4. Anatomic versus pathologic stage-group ambiguity (breast specimen 2-1-206).

Item S4.1 Original pathology report (excerpt, specimen 2-1-206)

```
1. Breast, 9 o'clock position / 5 cm from nipple, right, status post neoadjuvant
   chemotherapy, partial mastectomy, Invasive carcinoma of no special type, grade 2.
2. Lymph node, axillary, right, lymphadenectomy, carcinoma, metastatic (2/9).
3. Immunohistochemical study: ER (positive, strong, 99 %), PgR (positive, weak, 5 %),
   Her2/neu (Equivocal, score 2+) and Ki67 proliferation index (up to 3 %).
4. Pathologic AJCC Stage Group: ypT1c N1a, cM0
   (Anatomic stage group IIA, AJCC 8ed, combined with report of pathology number
    2024-35741).

   1. Procedure: Partial mastectomy
   …
```

Item S4.2 Model output (excerpt, specimen 2-1-206)


```
{
  "cancer_data": {
    "procedure": "partial_mastectomy",
    "cancer_quadrant": "lower_outer_quadrant",
    "cancer_clock": 9,
    "cancer_laterality": "right",
    "histology": "invasive_carcinoma_no_special_type",
    "tumor_size": 19,
    "treatment_effect": "Partial, Definite response",
    "...": "...",
    "grade": 2,
    "tnm_descriptor": "y",
    "pt_category": "t1c",
    "pn_category": "n1a",
    "pm_category": "mx",
    "pathologic_stage_group": "iib",
    "anatomic_stage_group": "iia"
  }
}
```


Supplementary Item S5. Surgical-technique misclassification — "3D" treated as `open` (stomach specimen 9-1-78).

Item S5.1 Original pathology report (excerpt, specimen 9-1-78)

```
1. Stomach, anastomosis of B-II, status post subtotal gastrectomy with B-II reconstruction,
   3D radical total gastrectomy,
     (1) large cell neuroendocrine carcinoma,
     (2) high-grade glandular dysplasia and
     (3) chronic gastritis and intestinal metaplasia.
2. Small intestine, anastomosis of B-II, 3D radical total gastrectomy,
   large cell neuroendocrine carcinoma, involved.
   …
10. Pathologic staging: pT3, pN0, cM0, stage IIA (AJCC, 8th edition).

   The specimen fixed in formalin is submitted in six separated bags labeled as
```

```
    1: stomach, 2: lymph node (LN) 1, 3: LN2, 4: LN3, 5: LN4, 6: LN789.

  1. Procedure: 3D radical total gastrectomy
  2. Specimen:
     Stomach: 11.5 × 6.5 × 1.8 cm in size …
```

Item S5.2 Model output (excerpt, specimen 2-1-206)


```
{
  "cancer_data": {
    "procedure": "total_gastrectomy",
    "surgical_technique": "open",
    "cancer_primary_site": "pylorus",
    "histology": "others",
    "grade": null,
    "tumor_extent": "penetrate_subserosal_connective_tissue_no_serosa",
    "...": "...",
    "pt_category": "t3",
    "pn_category": "n0"
  }
}
```


Supplementary Item S6. Lymph-node extraction in esophageal cancer (specimens 5-1-5 success and 5-1-3 partial omission).

Item S6.1 Successful LN extraction (specimen 5-1-5)

Item S6.1.1 Original pathology report (excerpt, specimen 5-1-5)

```
1. Esophagus, EG junction, thoracoscopic esophagectomy, status post CCRT,
   squamous cell carcinoma, well differentiated, residual.
   …
6. Lymph node, regional, lymphadenectomy, negative of malignancy (0/1).
7. Lymph node, group 7, lymphadenectomy, negative of malignancy (0/1).
8. Soft tissue, labeled as "LN 9L", excision, negative of malignancy (0).
9. Lymph node, group 10L, lymphadenectomy, negative of malignancy (0/1).
10. Pathologic Stage: ypT3 N0, cM0, stage IIB (AJCC, 8th edition).

   The specimen fixed in formalin is submitted in seven separated bags, labeled as
     1: esophagus, 2: stomach + liver, 3: LN7, 4: LN 9L, 5: LN 10L,
     6: anastomosis ring, and 7: gastric tube stump, respectively.
   Procedure / Specimen Type: Ivor Lewis procedure
   …
   F1-2: anastomosis ring (all for section)
   G1-2: gastric tube stump

   Microscopic Examination:
     1. Tumor Site: EG junction
     2. Histologic Type: squamous cell carcinoma
     3. Histologic Grade: G1, well differentiated
     …
     10. Regional Lymph Node Status: All lymph nodes are negative of malignancy
         Number of Lymph Nodes Involved/Examined: 0/3
         Nodal tumor size: not applicable
         Extracapsular extension: not applicable
     11. Pathologic Stage (pTNM, AJCC 8th Edition): …
```

Item S6.1.2 Model output (excerpt, specimen 5-1-5)


```
{
  "cancer_data": {
    "procedure": "esophagogastrectomy",
    "surgical_technique": "thoracoscopic",
    "cancer_primary_site": "gastroesophageal_junction",
    "...": "...",
    "tnm_descriptor": "y",
    "pt_category": "t3",
    "pn_category": "n0",
    "pm_category": "mx",
    "stage_group": "iib",
    "ajcc_version": 8,
    "margins": [],
    "regional_lymph_node": [
      {
        "lymph_node_category": "thoracic_7",
        "involved": 0,
        "examined": 1,
        "station_name": null
      },
      {
        "lymph_node_category": "thoracic_9l",
        "involved": 0,
        "examined": 1,
        "station_name": null
      }
    ]
  }
}
```

Item S6.2 Failed LN extraction with partial station omission (specimen 5-1-3)

Item S6.2.1 Original pathology report (excerpt, specimen 5-1-3)

```
1. Esophagus, lower third, thoracoscopic esophagectomy and gastric tube pullup
   reconstruction, Squamous cell carcinoma, moderately differentiated.
2. Stomach, cardia, thoracoscopic esophagectomy and gastric tube pullup reconstruction,
   Chronic active gastritis. Free of tumor.
   …
5. Lymph node, perigastric, lymphadenectomy, Negative of malignancy (0/8).
6. Lymph node, group 7, lymphadenectomy, Negative of malignancy (0/4).
7. Lymph node, group 8, lymphadenectomy, Negative of malignancy (0/4).
8. Lymph node, group 9, lymphadenectomy, Negative of malignancy (0/1).
9. Soft tissue, labeled as "group 9L", excision, Negative of malignancy. No lymph node is found.
10. Soft tissue, labeled as "group 10", excision, Negative of malignancy. No lymph node is found.
11. Lymph node, group 10L, lymphadenectomy, Negative of malignancy (0/2).
12. Lymph node, left gastric, lymphadenectomy, Squamous cell carcinoma, metastatic (1/1).
13. Pathologic Stage: pT1a, N1, cM0, stage IIB (AJCC, 8th edition).

   The specimen fixed in formalin is submitted in ten separated bags, labeled as
     1: esophagus and partial stomach, 2: esophageal proximal cut end, 3: anastomosis,
     4: LN 7, 5: LN 8, 6: LN 9, 7: LN 9L, 8: LN 10, 9: LN 10L, and 10: left gastric LN,
     respectively.

   Microscopic Examination:
```

```
1. Tumor Site: Lower third esophagus
2. Histologic Type: Squamous cell carcinoma
…
9. Perineural Invasion: Not identified
10. Regional Lymph Node Status:
    Number of Lymph Nodes Involved/Examined: 1/20
    Nodal tumor size: 1.0 cm
    Extracapsular extension: Not identified
```

Item S6.2.2 Model output (excerpt, specimen 5-1-5)


```
{
  "cancer_data": {
    "procedure": "esophagectomy",
    "surgical_technique": "thoracoscopic",
    "cancer_primary_site": "lower_third",
    "histology": "squamous_cell_carcinoma",
    "grade": 2,
    "...": "...",
    "pt_category": "t1a",
    "pn_category": "n1",
    "pm_category": "mx",
    "stage_group": "iib",
    "ajcc_version": 8,
    "margins": [
      {
        "margin_category": "proximal"
      }
    ],
    "regional_lymph_node": [
      {
        "lymph_node_category": "thoracic_7",
        "involved": 0,
        "examined": 1,
        "station_name": null
      }
    ],
    "regional_lymph_node": [
      {
        "lymph_node_category": "regional_gastric",
        "involved": 1,
        "examined": 1,
        "station_name": "left gastric"
      }
    ],
    "extranodal_extension": false,
    "maximal_ln_size": 10
  }
}
```


Supplementary Item S7. Margin-status over-population from schema enumeration (pancreas Whipple specimen 1-1-14).

Item S7.1 Original pathology report (excerpt, specimen 1-1-14)

```
1. Pancreas, head, status post neoadjuvant chemotherapy, pancreatoduodenectomy,
   Ductal adenocarcinoma, moderately differentiated.
```

```
2. Stomach, lower body and antrum, pancreatoduodenectomy, Free of tumor.
   …
14. Lymph node, group 16, lymphadenectomy, Negative for malignancy (0/3).
15. Liver capsule, excision, Simple cyst with dystrophic calcification.
16. Pancreas margin, excision, Free of tumor.
17. Pathologic staging: ypT2 pN1, cM1, stage IV (AJCC, 8th edition).

   The specimen fixed in formalin is submitted in two separated bags labeled as
     1: pancreaticoduodenectomy product, 2: group 8 lymph nodes (LN), 3: group 12 LNs,
     4: group 13 LNs, 5: group 14 LNs, 6: group 16 LNs, 7: liver capsule,
     and 8: pancreas margin, respectively.

   Procedure: Pancreaticoduodenectomy.
   …
   6. Macroscopic margin status:
      Pancreatic neck/parenchymal margin: Free of tumor, with the distance of 1.0 cm.
      Common bile duct margin: Free of tumor, with the distance of 2.5 cm.
      Proximal (gastric or duodenal) margin: Free of tumor, with the distance of 10 cm.
      Distal (duodenal or jejunal) margin: Free of tumor, with the distance of 23 cm.
      Anterior outmost margin of pancreas: Free of tumor, with the distance of 0.1 cm.
      Posterior outmost margin of pancreas: Free of tumor, with the distance of 0.1 cm.
      Uncinate (retroperitoneal/superior mesenteric artery) margin: Free of tumor,
        with the distance of 0.1 cm.
      SMV groove margin: Free of tumor, with the distance of 0.1 cm.
   7. Non-tumorous part: Fibrosis.

   Microscopic Examination:
     1. Histologic Type: Ductal Adenocarcinoma.
     2. Histologic Grade (ductal carcinoma only): G2, moderately differentiated.
     …
     7. Perineural Invasion: Present.
     8. Margin:
        a. Proximal pancreatic parenchymal margin: Not applicable.
        b. Distal pancreatic parenchymal margin: Not applicable.
        c. Pancreatic neck/parenchymal margin: Involved by invasive carcinoma in
           specimen 1 (section A11), but free of tumor in the separated margin
           (section H1 and frozen section 2024-31509).
        d. Uncinate (retroperitoneal/superior mesenteric artery) margin:
           Involved by Invasive carcinoma (section A19).
        e. Bile duct margin: Greater than 1.0 cm/10.0 mm in distance from invasive
           carcinoma to closest margin (section A5).
        f. Proximal (gastric or duodenal) margin: Greater than 1.0 cm/10.0 mm in distance
           from invasive carcinoma to closest margin.
        g. Distal (duodenal or jejunal) margin: Greater than 1.0 cm/10.0 mm in distance
           from invasive carcinoma to closest margin.
        h. Other (specify) margin: Posterior surface involved by invasive carcinoma
           (section A15).
     9. Regional Lymph Node Status:
        *Specify (Lymph Node Involved/Examination):
           regional (2/2), peri-intestinal (0/0), peri-gastric (0/0),
           group 8 (0/1), group 12 (0/2), group 13 (0/5), group 14 (0/3), group 16 (0/3).
     11. Pathologic Stage (pTNM, AJCC 8th Edition): …
```

Item S7.2 Model output (excerpt, specimen 1-1-14)


```
{
  "cancer_data": {
    "procedure": "whipple_procedure",
```

```
"tumor_site": "head",
"histology": "ductal_adenocarcinoma_nos",
"tumor_size": 25,
"...": "...",
"tnm_descriptor": "y",
"pt_category": "t2",
"pn_category": "n1",
"pm_category": "mx",
"overall_stage": "iv",
"ajcc_version": 8,
"margins": [
  {
    "margin_category": "pancreatic_neck",
    "margin_involved": false,
    "distance": 10,
    "description": "Free of tumor"
  },
  {
    "margin_category": "uncinate",
    "margin_involved": true,
    "distance": 0,
    "description": "Involved by invasive carcinoma"
  },
  {
    "margin_category": "bile_duct",
    "margin_involved": false,
    "distance": 10,
    "description": "Free of tumor"
  },
  {
    "margin_category": "proximal_gastric",
    "margin_involved": false,
    "distance": 10,
    "description": "Free of tumor"
  },
  {
    "margin_category": "proximal_duodenal",
    "margin_involved": false,
    "distance": 10,
    "description": "Free of tumor"
  },
  {
    "margin_category": "distal_intestinal",
    "margin_involved": false,
    "distance": 10,
    "description": "Free of tumor"
  },
  {
    "margin_category": "anterior_outmost",
    "margin_involved": false,
    "distance": 1,
    "description": "Free of tumor"
  },
  {
    "margin_category": "posterior_outmost",
    "margin_involved": false,
    "distance": 1,
    "description": "Free of tumor"
  },
```

```
        {
          "margin_category": "outmost",
          "margin_involved": false,
          "distance": 1,
          "description": "Free of tumor"
        },
        {
          "margin_category": "others",
          "margin_involved": true,
          "distance": 0,
          "description": "Involved by invasive carcinoma"
        }
      ],
      "regional_lymph_node": [
        {
          "lymph_node_category": "regional_pancreatic",
          "involved": 2,
          "examined": 2,
          "station_name": null
        }
      ],
      "regional_lymph_node": [
        {
          "lymph_node_category": "thoracic_7",
          "involved": 0,
          "examined": 1,
          "station_name": null
        },
        {
          "lymph_node_category": "thoracic_9l",
          "involved": 0,
          "examined": 1,
          "station_name": null
        }
      ]
    }
  }
```

Supplementary Item S8. TNM staging-basis: three structural dimensions of the anatomic-vs-pathologic stage_group gap, with two complementary mitigations.

Item S8.1 Headline observation

On the same 2,248 paired breast case-runs (Section 3.3.1), `anatomic_stage_group` was extracted at 97.95 % [97.28, 98.46] effective accuracy whereas `pathologic_stage_group` reached only 74.87 % [73.03, 76.62] — a 23.09 percentage-point gap whose 95 % Wilson intervals do not overlap. Cohen's κ remained at 0.93 against the gold for `pathologic_stage_group`, ruling out random-disagreement noise as the primary driver. On re-analysis the gap is *not* primarily a model-disambiguation failure but decomposes into three structurally distinct dimensions, each anchored in AJCC 8 breast-staging conventions (Amin et al., AJCC 8th edition).

Item S8.2 Dimension 1 — Source-availability gap for post-treatment (`y`-descriptor) cases

AJCC 8 directs the clinician toward a *clinical* prognostic group for `y` cases — a stage a pathology report cannot issue. Following clinicopathologic discussion at our institute during the AJCC 8 adoption period, post-

chemotherapy specimens were reported with anatomic_stage_group only; later reports include both stage groups. For the contiguous band of y-descriptor cases that print anatomic_stage_group alone, the registry-curated gold carries a pathologic_stage_group value inferred by the annotator from T, N, M, biomarker status, and the AJCC 8 stage table — information not present in the source narrative. The extraction model has no access to the staging table and correctly emits what the report contains.

Item S8.3 Dimension 2 — HER-2 2+ equivocal indeterminacy

AJCC 8 prognostic staging requires definitive HER-2 status; when immunohistochemistry returns HER-2 2+, fluorescence in-situ hybridisation (FISH) is required and the prognostic stage cannot be finalised from the pathology report alone. The true pathologic_stage_group for these cases takes one of two values depending on FISH amplification; a single-valued field prediction is structurally unable to match the gold on either side of the FISH outcome.

Item S8.4 Dimension 3 — Residual narrative ambiguity

A smaller residual of genuine narrative ambiguity remains in non-y reports that cite T, N, and M descriptors without an explicit "prognostic" or "anatomic" qualifier, where the model defaults to anatomic staging.

Item S8.5 Two complementary structural mitigations

Both mitigations are flagged for a follow-up revision because they require schema changes plus re-running the full extraction grid against the modified schema.

1. **AJCC 8 stage-table injection (addresses Dimensions 1 and 3).** Extend the DSPy schema so the model is provided with the AJCC 8 prognostic stage table and asked to *derive* pathologic_stage_group from anatomic_stage_group + ER + PgR + HER-2 + TNM descriptor, rather than echoing what the source narrative happens to print. This collapses the annotator-inferred-gold-vs-source-only-model gap by giving the model the same lookup the annotator used.
2. **Cardinality extension of `pathologic_stage_group` (addresses Dimension 2).** Widen pathologic_stage_group from a scalar to a list of candidate prognostic stages when HER-2 is reported as 2+ pending FISH. This mirrors the cardinality-extension pattern separately proposed in R2.2 for synchronous multi-primaries (widening cancer_data to a list of CancerEntity records): the type structure is unchanged; only the cardinality changes from one to many, and the hierarchical JSON namespace already accommodates this shape.

The two mitigations are complementary rather than alternative: the stage-table lookup recovers values the model cannot derive from the source narrative alone; the cardinality extension represents indeterminacy honestly when a single value is structurally insufficient. Both are amenable to ablation testing within an extended DSPy framework and are flagged as next-paper scope.

Supplementary Item S9. Multi-primary handling: three-axis future-work plan + cardinality-induced conflation caveat.

Item S9.1 Headline observation

The current `cancer_data` schema models a single primary tumor per report — a deliberate choice that reflects the registry-grade target of one extraction record per primary, but that creates a structural blind spot for synchronous multi-primary cases (e.g., bilateral breast carcinoma, multifocal lung primaries, multifoci hypopharynx–esophagus). The Stage-B triage analysis in Section Multi-Primary and Out-of-Scope Triage quantified this directly: of 1,138 gold-`"others"` case-runs across the thirty multi-seed runs, 449 (39.5 %) were correctly triaged to the `"others"` class, while 631 (55.4 %) were misrouted to a single in-scope organ — 180 of these (28.5 %) being synchronous multi-primaries within an in-scope organ system, 163 (25.8 %) being out-of-scope sites that the model approximated to a neighboring organ, and 288 (45.6 %) being free-text descriptions the regex could not confidently classify into either category.

Item S9.2 Three complementary future-work axes

One axis is deployable in the current revision; two require dedicated follow-up effort.

- **Axis 1 — Flag-for-review workflow (current revision).** Cases meeting any of the following triggers are routed to manual registrar reconciliation rather than auto-committed: `tumor_focality ∈ {multifocal, multicentric}`, `cancer_laterality = "bilateral"`, an existing `detect_multi_primary()` heuristic returning true (which already captures focality, laterality, multi-quadrant, multi-clock, and double-primary regex patterns; the module is part of the public release), or an explicit `cancer_category = "others"` triage call. This converts a silent misclassification into a human-readable flag and preserves the safety property required of an automated registry pipeline. The flag-for-review trigger requires no schema changes and can be deployed immediately on top of the existing pipeline.
- **Axis 2 — Schema cardinality extension (proposed for the next revision, not the current one).** The natural longer-term fix is to widen `cancer_data` from a single dictionary to a list of `CancerEntity` records, each carrying its own `tumor_focality`, `cancer_laterality`, `tnm`, and biomarker submodules. The hierarchical JSON namespace already accommodates this; the change is in *cardinality*, not in *type structure*. We do not include this extension in the current revision because it requires re-running the full extraction grid against the multi-entity schema and re-validating the gold standard for the affected case-runs — work that is appropriate for a follow-up paper on synchronous-primary registry coverage rather than an in-revision change.
- **Axis 3 — Detection-stage improvements (follow-up paper).** The deterministic `detect_multi_primary()` route is robust at a single institute but does not generalize across reporting conventions. Two complementary detectors are sketched. First, a prototype `secondary_cancer_category` field on the eligibility signature — drafted in the public release (common_v2.py) but unevaluated within the revision timeframe — would let the generative model emit a probabilistic "is there a second primary?" signal directly; whether an entropy-rich language model is the right substrate for a low-cardinality binary decision is itself the empirical question. Second, a discriminative BERT-style classifier, building on the Bio_ClinicalBERT baseline introduced in Section 3.10, would provide a more decisive inference than either the rule-based heuristic or the generative-LLM probe; this is the natural complement when deterministic detection cannot generalize across reporting styles.

Item S9.3 Out-of-scope sites — a distinct problem

We treat out-of-scope sites not as a multi-primary failure but as a coverage gap to be closed by additional CAP-aligned organ modules (the schema framework supports incremental organ addition without disrupting the existing ten modules). Until those modules are added, `"others"` remains a confident triage class — its operational value is precisely that it surfaces cases the current schema cannot represent, rather than silently shoehorning them into an in-scope organ.

Item S9.4 Cardinality-induced conflation caveat (applies to all three axes)

A final caveat applies to *all three future-work axes*: detecting a second primary correctly does not guarantee that the downstream extraction produces two clean records, and two distinct problems sit beneath that guarantee.

First, the single-dictionary `cancer_data` schema holds only one TNM stage, one focality, one laterality, one biomarker submodule, and one tumor size, so cases with two physically separate primaries — bilateral breast carcinoma, separate-lobe lung primaries, multifocal pancreaticobiliary tumors — place every field at risk of conflation across the two lesions; the conflation is most visible on variable-length list fields such as regional lymph nodes and surgical margins, where ambiguity surfaces explicitly, but the limitation is schema-wide rather than list-field-specific.

Second, even when the schema is widened to two `CancerEntity` records, the model must still solve an attribute–lesion binding problem so that each extracted attribute lands on the correct lesion — pathology reports routinely interleave laterality with finding-type ("Left breast IDC pT2N1Mx; Right breast ILC pT1N0Mx"), and generative extraction is observed qualitatively to muddle this coreference.

Quantifying the cardinality-induced conflation rate and the post-extension binding-error rate is deferred to a follow-up study on synchronous-primary registry coverage. In the interim, the flag-for-review workflow remains the operational safety net because it does not require the model to be correct on either downstream layer, and the schema-cardinality extension above is therefore best understood as a *necessary but not sufficient* structural prerequisite for honest two-primary records rather than a fidelity refinement on top of the current schema.

Supplementary Item S10. Field-level error attribution: the six-mechanism (M1–M6) taxonomy with per-mechanism deep dives.

Item S10.1 Framing

A confusion-matrix-level audit of the eleven bottom-decile fields (Section 3.3.2) supports a six-mechanism attribution that the headline accuracy alone cannot resolve, and that our first-round four-bucket attempt mis-categorized in places. The six mechanisms are:

- **M1** source-bound qualitative phrasing
- **M2** heterogeneous reporting / null-on-no-clue
- **M3** anatomic ontology gap
- **M4** AJCC convention enforcement
- **M5** schema-shape
- **M6** unresolved model-side semantic conflation

Each is examined below.

Item S10.2 M1 — Source-bound qualitative phrasing (no current bottom-decile field)

No bottom-decile field on the current 30-run denominator manifests **source-bound qualitative phrasing** (M1) in its pure form; the M1 category remains in the taxonomy because it is a real failure mode for fields like `tumor_percentage` and `prostate_weight` that may surface in future runs.

Item S10.3 M2 — Heterogeneous reporting / null-on-no-clue (four fields)

Four fields fall under **heterogeneous reporting / null-on-no-clue (M2)**: `surgical_technique`, `cancer_clock`, `cancer_quadrant`, and `tumor_necrosis`. The previous response labelled these as source-bound, but per-organ heterogeneity and confusion-pattern audit tell a different story. `surgical_technique` ranges from 48.5 % in stomach to 99.8 % in prostate (Supplementary Table S29), a 51 pp spread driven by `3D` and `robotic` prefix vocabulary the schema does not currently disambiguate; a small prefix glossary plus a `[no prefix] → open` default rule resolves most of the gap. The analogous fix for `cancer_clock` / `cancer_quadrant` is a clock→quadrant lookup table with explicit null-on-ambiguity for 12/3/6/9 o'clock readings, plus null-on-no-clue. Thyroid `tumor_necrosis` is the same null-on-no-mention pattern at a different field — the source report often does not mention tumor necrosis at all, and the model is insufficiently biased toward null, committing instead to a `True/False` value when the correct extraction is null. None of these is residual source ambiguity; all four are engineering-trivial fixes queued for the next pipeline iteration.

Item S10.4 M4 — AJCC convention enforcement (one field)

One field — `distant_metastasis` (19.1 % at $\kappa = 0.95$) — is **AJCC convention enforcement** (M4): the documented AJCC 8 rule treats absent-evidence metastasis as `pM not applicable` (`Mx`, not `pM0`), and the very low effective accuracy at near-perfect κ reflects inconsistent application of this rule across the corpus.

Item S10.5 M5 — Schema-shape (four fields, three sub-mechanisms)

Four further fields are **schema-shape** problems (M5):

- pathologic_stage_group is decomposed in Section 3.3.1 into three structural dimensions (y-descriptor source gap, HER-2 2+ FISH indeterminacy, residual narrative ambiguity); the full decomposition is in Appendix A8.
- tnm_descriptor has both a cardinality issue (the field should be List[StrEnum] | None to allow y/p/c/r/ypT co-occurrence) and a measurable hallucination bias toward the rare recurrent (r) descriptor that a DSPy null-bias would substantially reduce.
- The two breast biomarker fields (biomarker_her2, biomarker_ki67) manifest a third M5 sub-mechanism — **schema over-specification**. The HER-2 schema exposes score, percentage, and positivity slots when HER-2 in fact has only a clinically-meaningful score, and the Ki-67 schema exposes score, percentage, and positivity when Ki-67 has only a clinically-meaningful percentage; the extraneous slots invite the model to hallucinate values that have no source basis.

All three M5 sub-problems are schema-level reworks rather than further model tuning. The biomarker schema rework was identified on re-audit but is too invasive to ship in this revision.

Item S10.6 M3 — Anatomic ontology gap (`procedure[liver]`, lymph-node stations)

The deepest mechanism — **anatomic ontology gap** (M3) — does not appear in Figure 5 because procedure is above 90 % globally, but procedure[liver] is 85.0 % and is the canonical case. Mapping a free-text operative name onto the controlled CAP partial-hepatectomy categories requires segment-counting and laterality reasoning that local LLMs do not natively perform: 3D right posterior sectionectomy involves segments VI/VII (partial hepatectomy, minor), left hepatectomy involves four segments (partial hepatectomy, major), and subsegmentectomy / liver transplantation / S6 liver resection each map differently. The same shape of failure drives the per-station canonicalization errors on lymph-node groups (Section 3.4). A registry-grade anatomic-ontology framework — addressing both lymph-node-station canonicalization and procedure-to-CAP-category mapping under a shared glossary methodology — is the subject of a separate manuscript currently in preparation; the present work reports the M3 gap as an explicit, structurally-bounded limitation rather than claiming to close it.

Item S10.7 M6 — Unresolved model-side semantic conflation (colorectal tumor_invasion)

Colorectal tumor_invasion (88.2 % at κ = 0.84) is the only bottom-decile field in the **unresolved model-side semantic conflation** category (M6). Confusion-matrix audit shows that 136 of 254 errors are gold = pericolorectal_tissue (pT3) → pred = muscularis_propria (pT2): the AJCC 8 pT3 definition is *"tumor invades through the muscularis propria into pericolorectal tissues"*, and the model appears to read "through muscularis" and emit the lower-tier muscularis_propria (pT2). We do not have an engineering remedy for this conflation. The author's anecdotal observation is that the downstream pT value remains correct on these cases (the conflation is at the depth-of-invasion enum and is not propagated to the staging output), but this is not separately verified in the present revision and is offered as a candid open limitation rather than a defended-away non-issue.

Item S10.8 Audit-pending — pancreas `tumor_extension`

Pancreas tumor_extension (77.1 % at κ = 0.61) is listed in Figure 5 as **audit-pending**. Confusion-matrix audit isolates the dominant error as under-staging at the pT3/pT4 boundary (110 of 172 errors are gold = adjacent_organs_structures → pred = peripancreatic_soft_tissue); pancreas has no muscularis-propria staging boundary, so this is not the M6 pattern. The underlying mechanism is not yet determined and we decline to assign a speculative category in this revision.

Item S10.9 What the taxonomy buys us

Notably, no bottom-decile error stemmed from parse failure or schema-validation breakage; the parse-error rate across all 192 registry fields and thirty runs was 0.0 %. This decomposition reframes the residual gap from a generic "model accuracy is bounded at 60–90 % for hard fields" critique to a structured understanding of *which* part of the gap is closable by schema work (M5), *which* by deterministic post-processing (M2, M4), *which* requires a structured anatomic ontology and is therefore next-paper scope (M3), *which* is bounded by registrar documentation practices and not closable by extraction-model engineering alone (M1), and *which* remain open research questions (M6 and the audit-pending pancreas row).

Item S10.10 List-typed-module addendum

The same six-mechanism principle applies to the list-typed modules. For surgical margins (Section 3.4), the dominant error is **schema-driven hallucination** — the model emits an organ-specific category enumeration that is sometimes absent from the underlying narrative. This is conservative for the operationally critical "any-margin-positive" endpoint (every hallucinated entry defaults to `margin_involved = false`, which cannot generate a positivity false-negative) but inflates the per-station hallucination rate. For lymph-node groups (Section 3.4), the dominant error is **station-name canonicalization** — recall and precision diverge in the same direction (e.g., prostate group recall 0.578 vs precision 0.923) because the model collapses or normalizes free-text station strings in a way that fails the strict category alignment used for scoring. This is the M3 ontology gap surfacing at the list level rather than at the scalar level, and is addressed by the same forthcoming companion paper. Neither list-level error mode affects the clinical-safety endpoints (margin positivity, any-positive nodal status), which remain at saturation.
